# Supplementary material for: Guadecitabine plus ipilimumab in unresectable melanoma: five-year follow-up and integrated multi-omic analysis in the phase 1b NIBIT-M4 trial
Source: Nat Commun. 2023 Sep 22;14:5914. doi: 10.1038/s41467-023-40994-4 (PMC10516894; doi:10.1038/s41467-023-40994-4)
Supplement: Supplementary file 1 — Supplementary Information [file 41467_2023_40994_MOESM1_ESM.pdf]

Patient	Dose	BOR	WES				RNASeq			RRBS-Seq			NanoString		
			Normal	W0	W4	W12	W0	W4	W12	W0	W4	W12	W0	W4	W12
1	30	NR	✓	✓	✓	✓	✓	✓	✓	✓	✓	✓	✓	✓	
2	30	NR	✓	✓	✓	✓	✓	✓	✓	✓	✓	✓	✓	✓	✓
3	30	R	✓	✓	✓	✓	✓	✓	✓	✓	✓	✓	✓	✓	✓
4	45	R	✓	✓	✓	✓	✓	✓	✓	✓	✓	✓	✓	✓	✓
5	45	R	✓	✓	✓	✓	✓	✓	✓	✓	✓	✓	✓	✓	
6	45	NR	✓	✓	✓		✓	✓		✓	✓		✓	✓	✓
7	45	NR	✓	✓	✓	✓	✓	✓	✓	✓	✓	✓	✓	✓	✓
8	45	NR	✓	✓	✓	✓	✓	✓	✓	✓	✓	✓	✓	✓	✓
9	45	NR		✓	✓	✓	✓	✓	✓	✓	✓	✓	✓	✓	✓
10	60	R		✓	✓	✓	✓	✓	✓	✓	✓	✓	✓	✓	✓
11	60	R		✓	✓	✓	✓	✓	✓	✓	✓	✓	✓	✓	✓
12	60	NR		✓	✓	✓	✓	✓	✓	✓	✓	✓	✓	✓	✓
13	60	R		✓	✓	✓	✓	✓	✓	✓	✓	✓	✓	✓	✓
14	60	NR		✓	✓	✓	✓	✓	✓	✓	✓	✓	✓	✓	✓

Supplementary Figure 1. *NIBIT-M4 cohort.*

Overview of sample profiling and clinical information for N=14 patients of NIBIT-M4 trial.

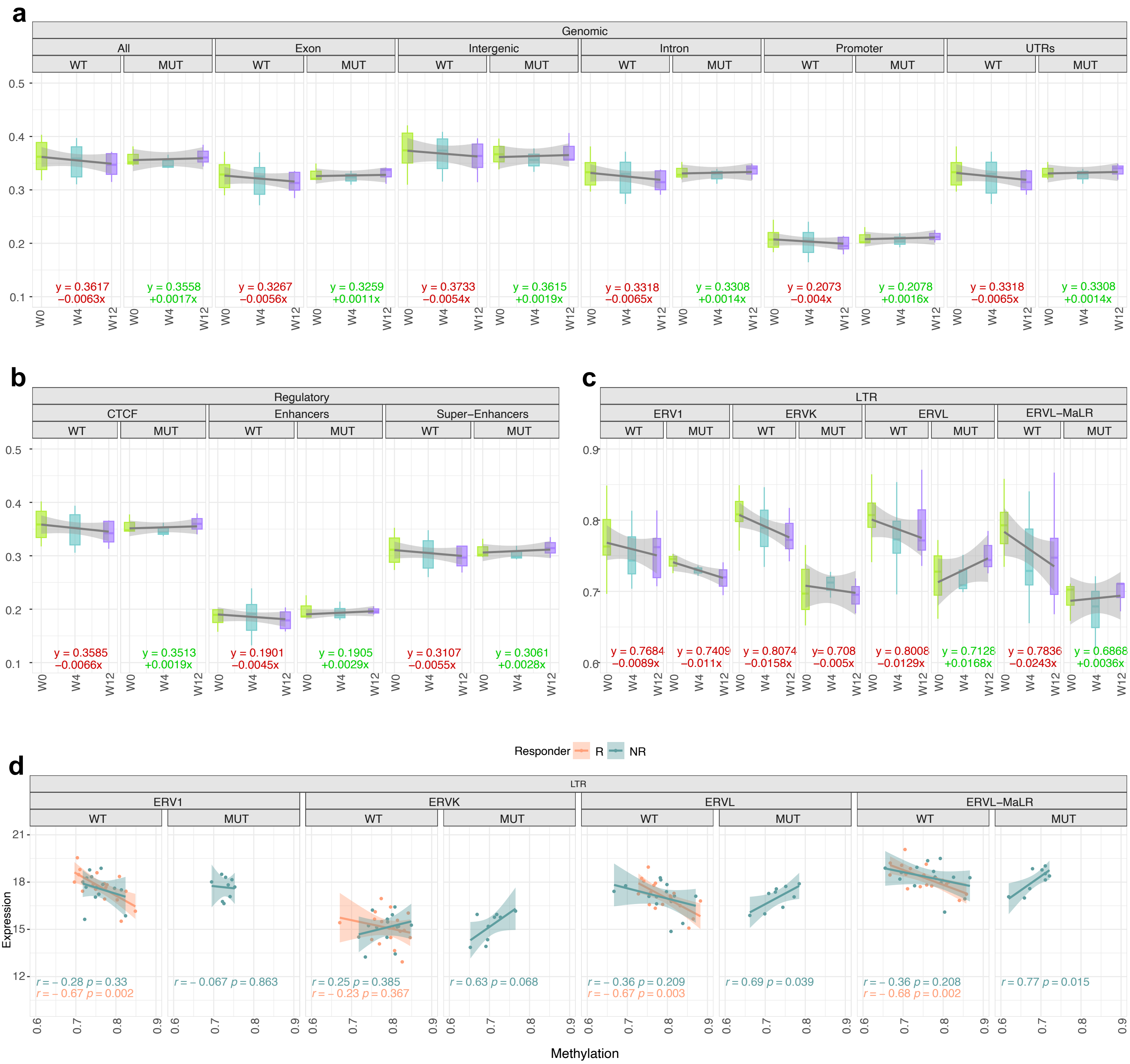

Supplementary Figure 2. *Evaluation of the methylation pattern for lesions harboring mutation in chromatin organization* (*DNMT1 or SETD2*)**.**

a Global Methylation pattern as function of the time of biopsy in genomics regions for *DNMT1* and *SETD2* mutant and wild-type lesions. The increasing (green) or decreasing (red) trend was evaluated based on the inclination of the robust linear regression line between the three time points (mutant patients n=3, wild-type patients n=11). **b** Regulatory regions (mutant patients n = 3, wild-type patients n = 11). **c** Classes of long terminal repeat (LTR) (mutant patients n = 3, wild-type patients n = 11). **d** Correlation between expression and methylation of LTR in mutant and wild-type cases (Spearman's correlation coefficient (*r*) and associated *p*-values from two-tailed correlation test). Lines represent robust least squares linear regression. In **a**, **b**, **c**, and **d**, bands represent confidence intervals (± 0.95) around a linear model fitted by robust regression using an M estimator. Source data are provided as a Source Data file.

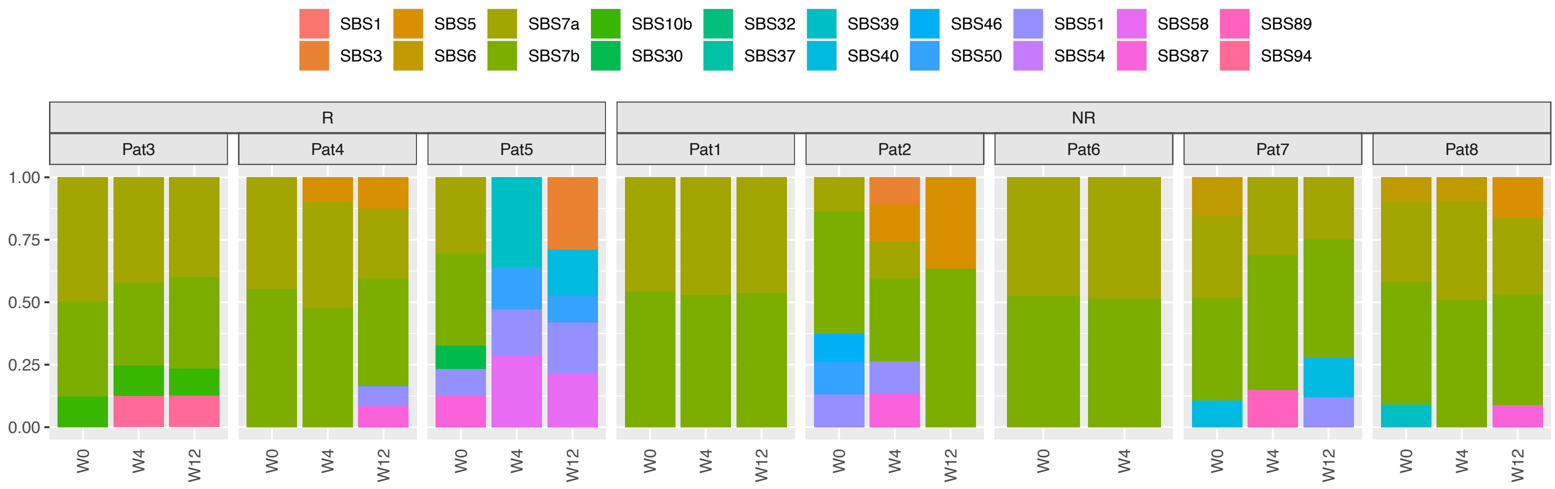

Supplementary Figure 3. *Mutational signature analysis for patients with available matched normal*. Deconvolution of the mutational patterns on COSMIC collection of mutational signatures v3.2. Source data are provided as a Source Data file.

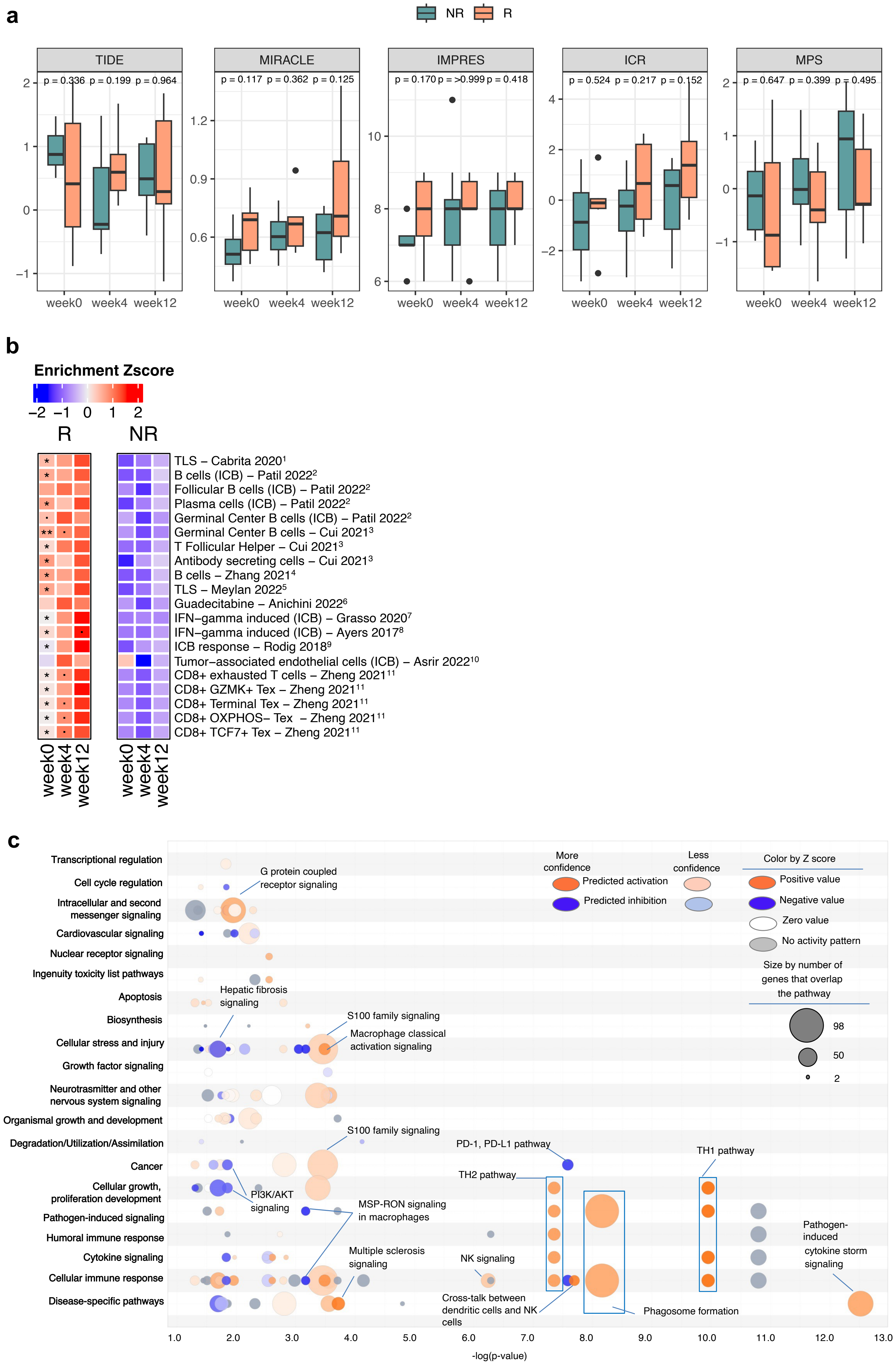

a
NR
R
TIDE
p = 0.336 p = 0.199 p = 0.964
MIRACLE
p = 0.117 p = 0.362 p = 0.125
IMPRES
p = 0.170 p = >0.999 p = 0.418
ICR
p = 0.524 p = 0.217 p = 0.152
MPS
p = 0.647 p = 0.399 p = 0.495
week0 week4 week12
b
Enrichment Zscore
−2 −1 0 1 2
R
NR
TLS – Cabrita 2020[1]
B cells (ICB) – Patil 2022[2]
Follicular B cells (ICB) – Patil 2022[2]
Plasma cells (ICB) – Patil 2022[2]
Germinal Center B cells (ICB) – Patil 2022[2]
Germinal Center B cells – Cui 2021[3]
T Follicular Helper – Cui 2021[3]
Antibody secreting cells – Cui 2021[3]
B cells – Zhang 2021[4]
TLS – Meylan 2022[5]
Guadecitabine – Anichini 2022[6]
IFN–gamma induced (ICB) – Grasso 2020[7]
IFN–gamma induced (ICB) – Ayers 2017[8]
ICB response – Rodig 2018[9]
Tumor–associated endothelial cells (ICB) – Asrir 2022[10]
CD8+ exhausted T cells – Zheng 2021[11]
CD8+ GZMK+ Tex – Zheng 2021[11]
CD8+ Terminal Tex – Zheng 2021[11]
CD8+ OXPHOS– Tex – Zheng 2021[11]
CD8+ TCF7+ Tex – Zheng 2021[11]
week0 week4 week12
week0 week4 week12
c
Transcriptional regulation
Cell cycle regulation
Intracellular and second messenger signaling
Cardiovascular signaling
Nuclear receptor signaling
Ingenuity toxicity list pathways
Apoptosis
Biosynthesis
Cellular stress and injury
Growth factor signaling
Neurotrasmitter and other nervous system signaling
Organismal growth and development
Degradation/Utilization/Assimilation
Cancer
Cellular growth, proliferation development
Pathogen-induced signaling
Humoral immune response
Cytokine signaling
Cellular immune response
Disease-specific pathways
G protein coupled receptor signaling
Hepatic fibrosis signaling
S100 family signaling
Macrophage classical activation signaling
S100 family signaling
PD-1, PD-L1 pathway
TH1 pathway
TH2 pathway
PI3K/AKT signaling
MSP-RON signaling in macrophages
Multiple sclerosis signaling
NK signaling
Cross-talk between dendritic cells and NK cells
Phagosome formation
Pathogen-induced cytokine storm signaling
More confidence
Less confidence
Predicted activation
Predicted inhibition
Color by Z score
Positive value
Negative value
Zero value
No activity pattern
Size by number of genes that overlap the pathway
98
50
2
1.0 2.0 3.0 4.0 5.0 6.0 7.0 8.0 9.0 10.0 11.0 12.0 13.0
-log(p-value)

Supplementary Figure 4. *Biomarkers and functional characterization of NIBIT-M4 trial.*

a ICI response prediction scores between R (n = 18) and NR (n = 23) samples at all time points (*p*-value from two-sided Student's t-Test). Box plots show the median as center, the lower and upper hinges that correspond to the 25th and the 75th percentile, and whiskers that extend to the smallest and largest value no more than 1,5*IQR. Values that stray more than 1.5*IQR upwards or downwards from the whiskers are considered potential outliers and represented with dots. **b** Heatmap of enrichment z-scores (from NANOSTRING gene expression assay) computed for selected pathways between R (n = 18) and NR (n = 22) samples grouped for treatment timepoints. Symbols within squares represent significance levels for each treatment timepoint (*p*-value from two-sided Student's t-Test: *: p <0.05, **: p < 0.01, ***: p < 0.001)). **c** Dot plot of enriched canonical pathways from Ingenuity Pathway Analysis (IPA) using significant differentially expressed genes between R (n = 18) vs NR (n = 23) samples at all time points (FDR < 0.1, from glmLRT as implemented in EdgeR[12]) as input. x axis represents the -log(*p*-value) from right-tailed Fisher exact test. Source data are provided as a Source Data file.

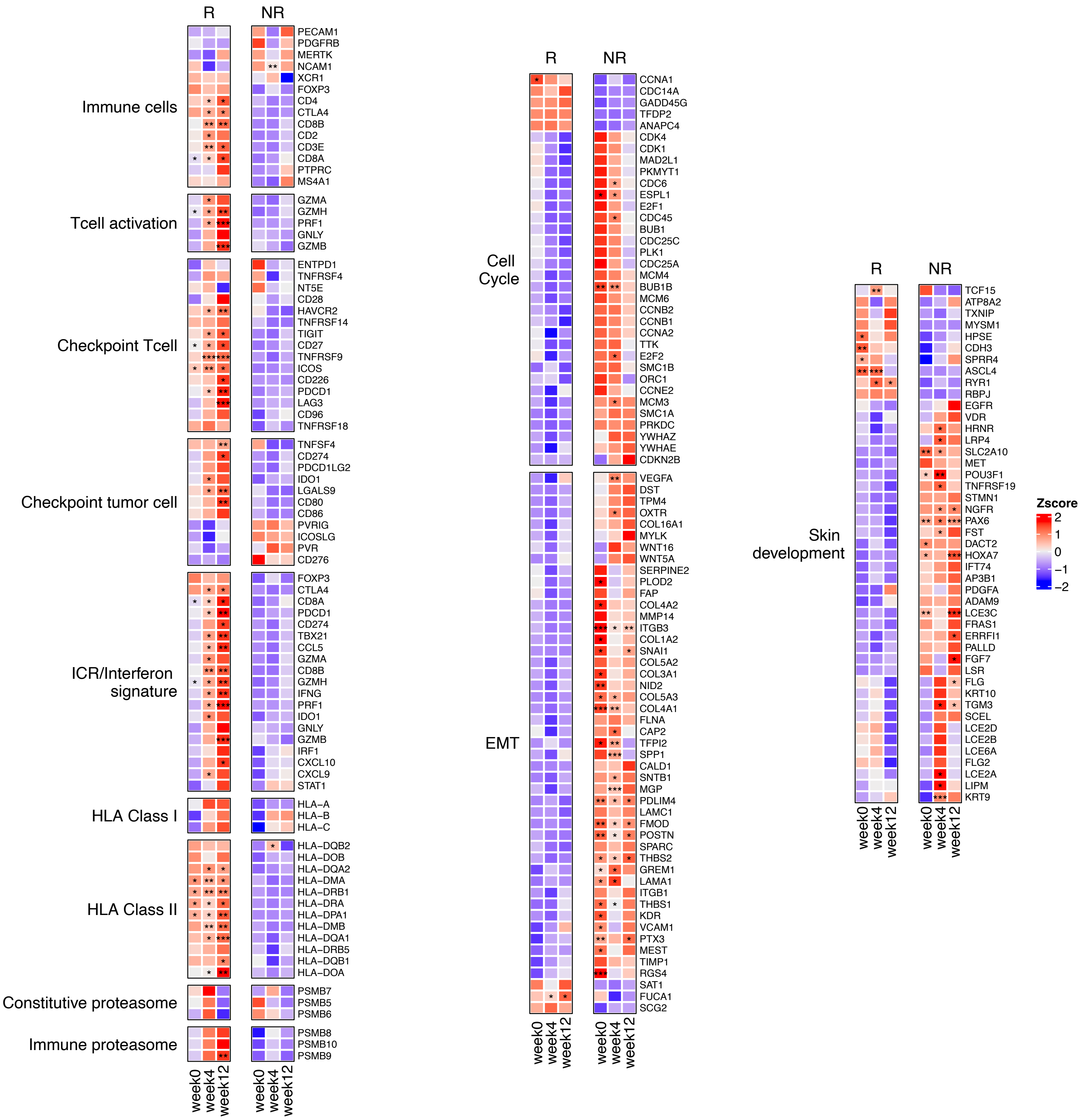

Supplementary Figure 5. *Evaluation of specific signatures in NIBIT-M4 trial*.

Heatmap of expression z-scores (from RNASeq profiling) computed for genes belonging to selected pathways between R (n = 6) and NR (n = 8) patient groups. The statistical significance of differential expression between R vs. NR was evaluated at each treatment timepoint (week 0, week 4, and week12), separately. Symbols within squares represent the *p*-value from two-sided Student's t-Test for each pairwise comparison (*: p <0.05, **: p < 0.01, ***: p < 0.001).

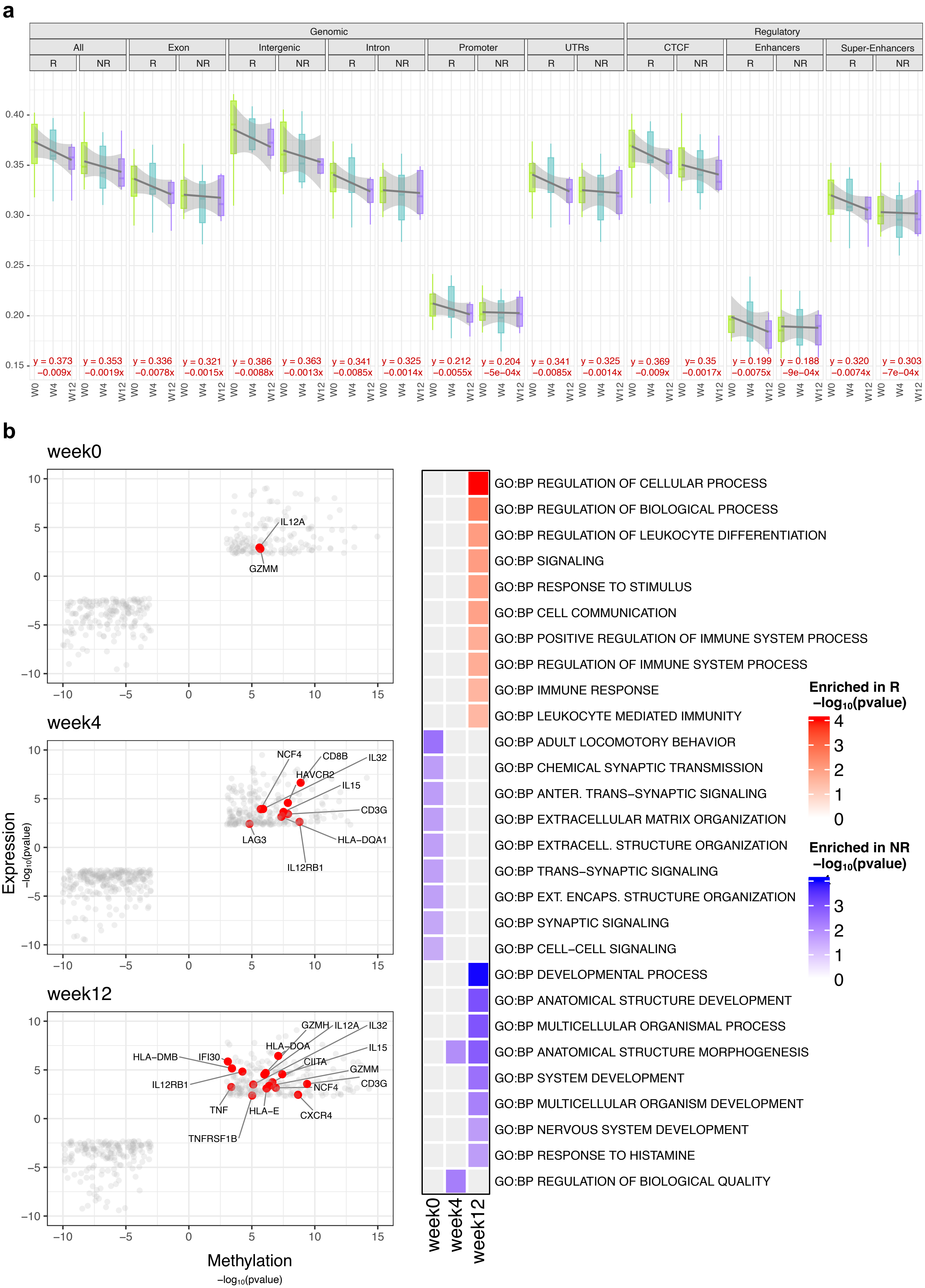
a
Genomic
Regulatory
All
Exon
Intergenic
Intron
Promoter
UTRs
CTCF
Enhancers
Super-Enhancers
R
NR
0.40
0.35
0.30
0.25
0.20
0.15
y = 0.373 −0.009x
y = 0.353 −0.0019x
y = 0.336 −0.0078x
y = 0.321 −0.0015x
y = 0.386 −0.0088x
y = 0.363 −0.0013x
y = 0.341 −0.0085x
y = 0.325 −0.0014x
y = 0.212 −0.0055x
y = 0.204 −5e−04x
y = 0.341 −0.0085x
y = 0.325 −0.0014x
y = 0.369 −0.009x
y= 0.35 −0.0017x
y = 0.199 −0.0075x
y = 0.188 −9e−04x
y = 0.320 −0.0074x
y = 0.303 −7e−04x
W0
W4
W12
b
week0
week4
week12
Expression
−log10(pvalue)
Methylation
−log10(pvalue)
IL12A
GZMM
NCF4
CD8B
IL32
HAVCR2
IL15
CD3G
LAG3
HLA−DQA1
IL12RB1
GZMH
HLA−DOA
HLA−DMB
IFI30
CIITA
NCF4
CXCR4
TNF
HLA−E
TNFRSF1B
GO:BP REGULATION OF CELLULAR PROCESS
GO:BP REGULATION OF BIOLOGICAL PROCESS
GO:BP REGULATION OF LEUKOCYTE DIFFERENTIATION
GO:BP SIGNALING
GO:BP RESPONSE TO STIMULUS
GO:BP CELL COMMUNICATION
GO:BP POSITIVE REGULATION OF IMMUNE SYSTEM PROCESS
GO:BP REGULATION OF IMMUNE SYSTEM PROCESS
GO:BP IMMUNE RESPONSE
GO:BP LEUKOCYTE MEDIATED IMMUNITY
GO:BP ADULT LOCOMOTORY BEHAVIOR
GO:BP CHEMICAL SYNAPTIC TRANSMISSION
GO:BP ANTER. TRANS−SYNAPTIC SIGNALING
GO:BP EXTRACELLULAR MATRIX ORGANIZATION
GO:BP EXTRACELL. STRUCTURE ORGANIZATION
GO:BP TRANS−SYNAPTIC SIGNALING
GO:BP EXT. ENCAPS. STRUCTURE ORGANIZATION
GO:BP SYNAPTIC SIGNALING
GO:BP CELL−CELL SIGNALING
GO:BP DEVELOPMENTAL PROCESS
GO:BP ANATOMICAL STRUCTURE DEVELOPMENT
GO:BP MULTICELLULAR ORGANISMAL PROCESS
GO:BP ANATOMICAL STRUCTURE MORPHOGENESIS
GO:BP SYSTEM DEVELOPMENT
GO:BP MULTICELLULAR ORGANISM DEVELOPMENT
GO:BP NERVOUS SYSTEM DEVELOPMENT
GO:BP RESPONSE TO HISTAMINE
GO:BP REGULATION OF BIOLOGICAL QUALITY
Enriched in R
−log10(pvalue)
Enriched in NR
−log10(pvalue)

Supplementary Figure 6. *Evaluation of the methylation pattern for R and NR lesions.*

a Global Methylation pattern as function of the time of biopsy in genomics and regulatory regions for R (n = 18) and NR (n = 23) lesions. The increasing (green) or decreasing (red) trend was evaluated based on the inclination of the robust linear regression line between the three time points. Bands represent confidence intervals (± 0.95) around a linear model fitted by robust regression using an M estimator. **b** Starburst plot of hyper-methylated and down-regulated genes (lower-left quadrant), and hypo-methylated and up-regulated genes (upper-right quadrant) between R (n = 6) and NR (n = 8) patients at different weeks of treatment (left). x and y axis represent the –log(*p*-value) multiplied by the sign of the difference from the differential methylation (from linear model, as implemented in the limma package[13]) and expression analyses (from glmLRT, as implemented in EdgeR[12]), respectively (left). Heatmap of enrichment scores as -log10(*p*-value) from GO:BP over-representation analysis (as implemented in SMITE package[14]) for hypo-methylated and up-regulated genes between R (n = 6) and NR (n = 8) patients at different weeks of treatment (right). Source data are provided as a Source Data file.

a

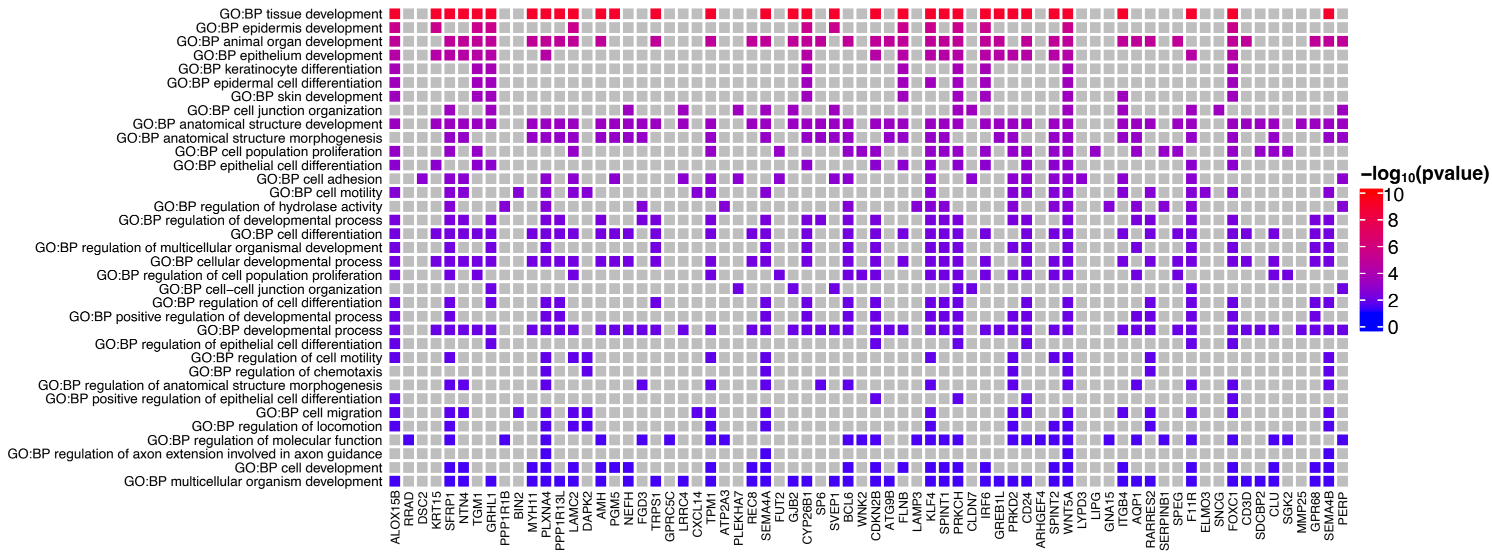

b

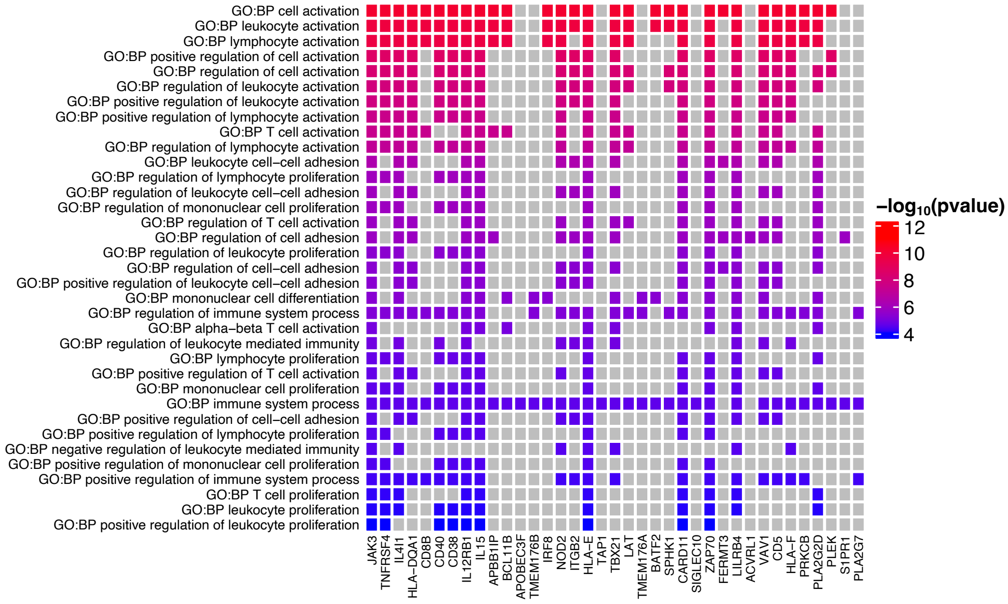

Supplementary Figure 7. *Longitudinal Functional Analysis in NIBIT-M4 trial.*

a Heatmap displaying the most significant GO:BP terms enriched (from over-representation analysis, as implemented in SMITE package[14]) by the top 100 genes upregulated and hypomethylated at week12 vs week0 in NR (n = 8) patients. **b** Same as in **a** for R (n = 6) patients. Boxes are colored according to the adjusted *p*-values of the enrichment. Terms with an adjusted *p*-value (SCS multiple testing correction implemented in gProfiler[15]) greater than 0.05 were filtered out. Only genes mapping to selected GO:BP terms were displayed.

a

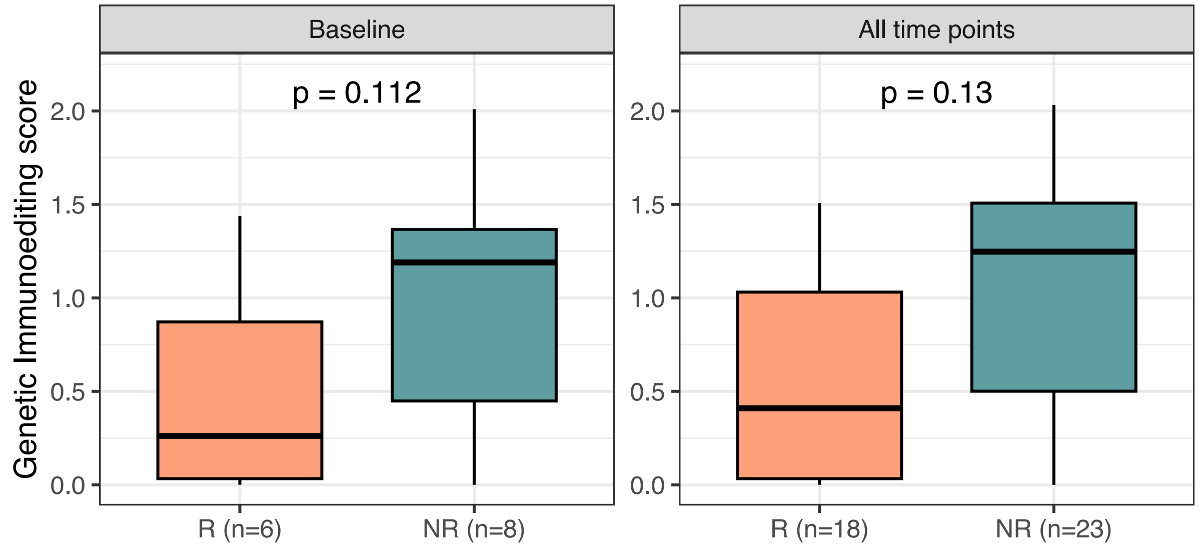

b

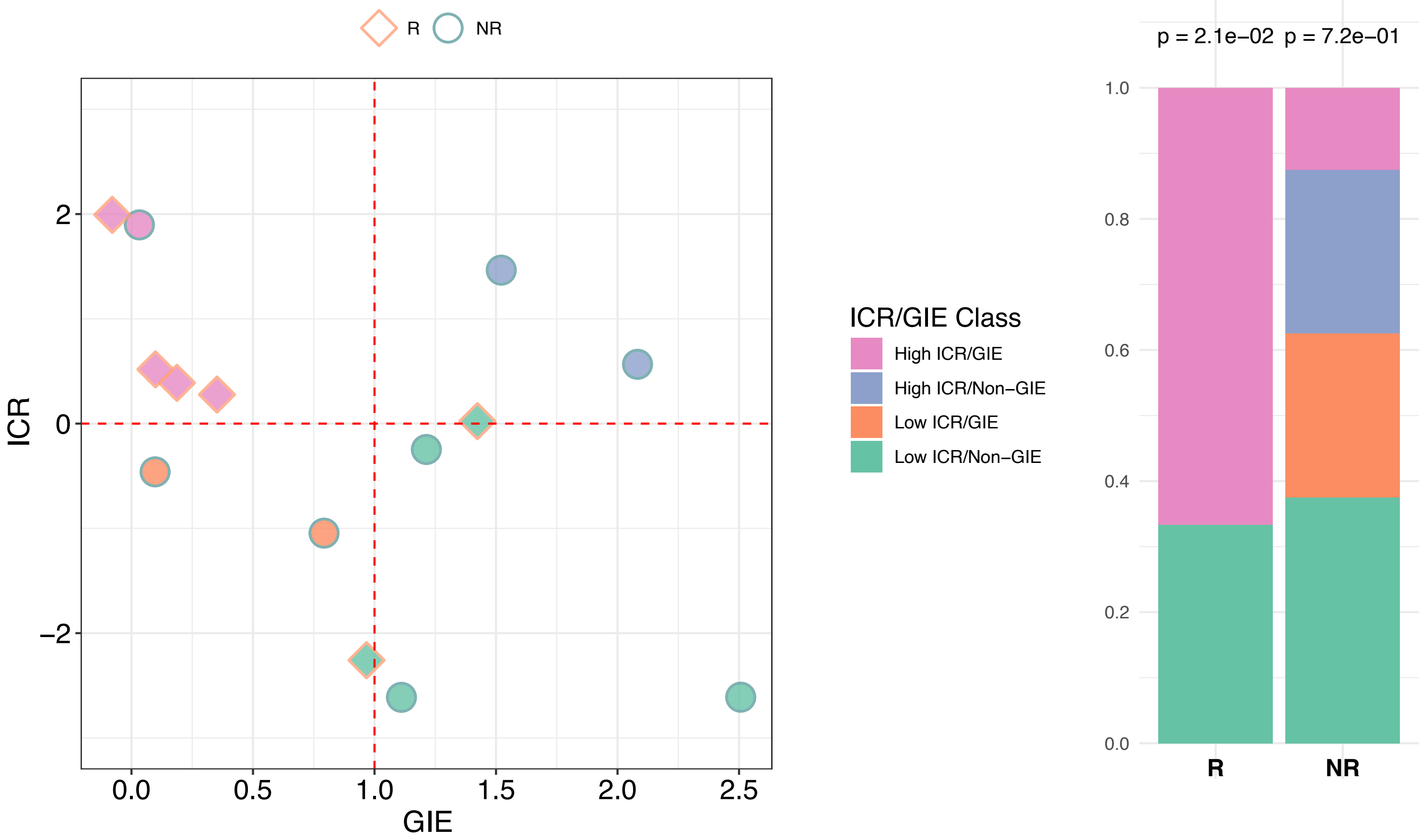

Supplementary Figure 8. *GIE at baseline of treatment in NIBIT-M4 trial.*

a Boxplot of Genetic Immunoediting (GIE) score at baseline (left, *p*-value of two-sided Student's t-Test) and all time points (right, *p*-value of two-way Mixed-model ANOVA, within-subjects factor: time of treatments; between-subjects factor: response) between R and NR patients. **b** Scatterplot of ICR score by GIE for R (n = 6) and NR (n = 8) patients at baseline (left) and their proportion (right) after classification as ICR/GIE classes (*p*-value from two-sided Pearson's chi-squared test statistic). Source data are provided as a Source Data file.

a

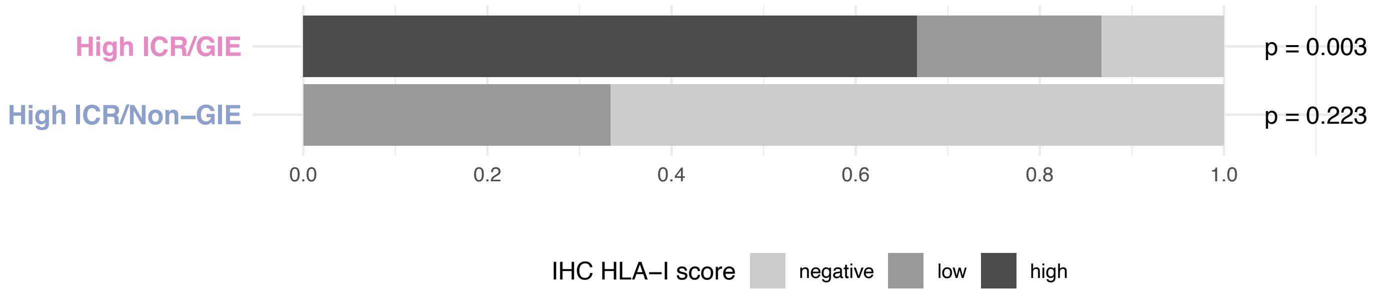

High ICR/GIE
High ICR/Non−GIE
p = 0.003
p = 0.223
0.0
0.2
0.4
0.6
0.8
1.0
IHC HLA−I score
negative
low
high

b

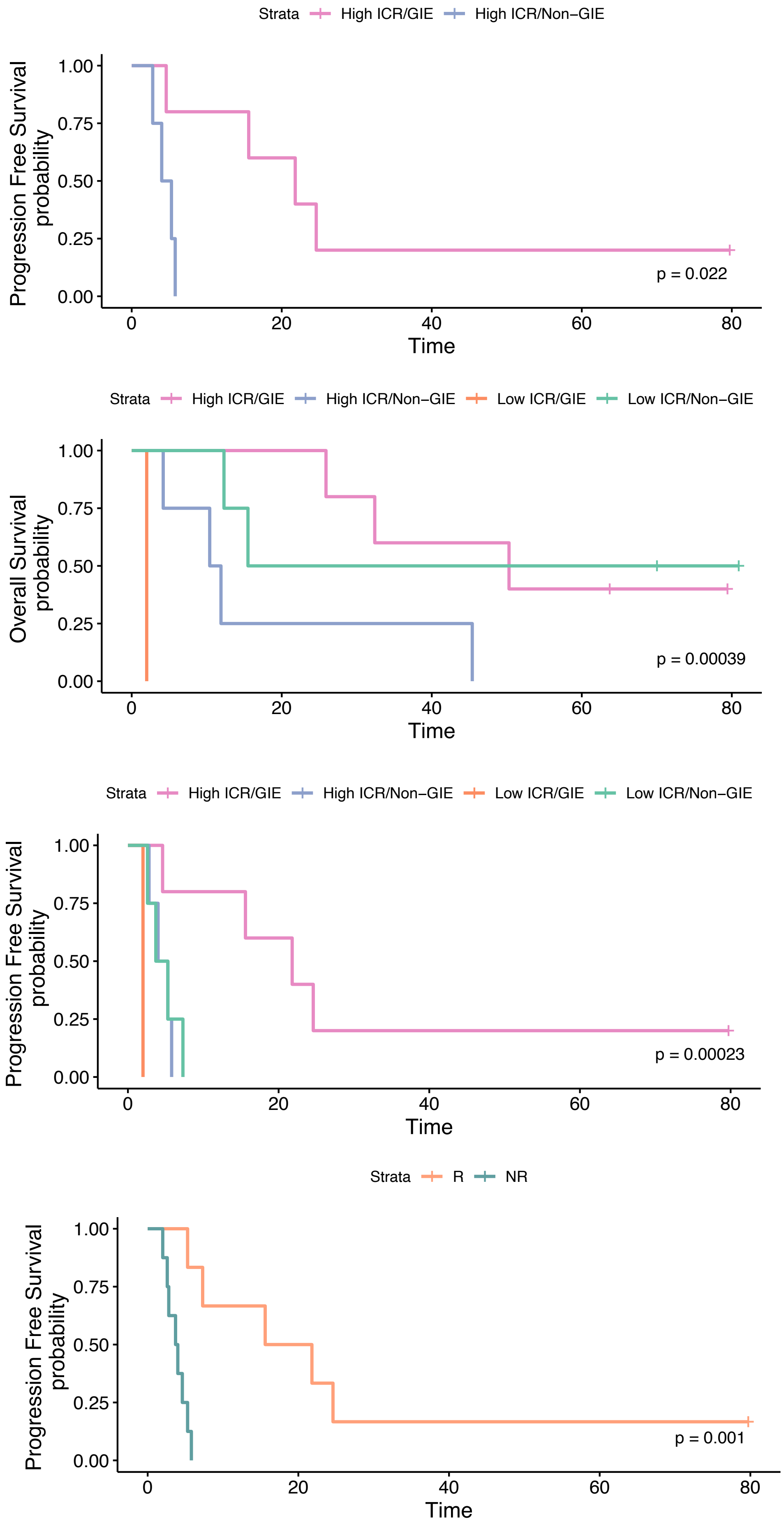

Strata
High ICR/GIE
High ICR/Non−GIE
Progression Free Survival probability
1.00
0.75
0.50
0.25
0.00
p = 0.022
0
20
40
60
80
Time
Strata
High ICR/GIE
High ICR/Non−GIE
Low ICR/GIE
Low ICR/Non−GIE
Overall Survival probability
p = 0.00039
Time
Strata
High ICR/GIE
High ICR/Non−GIE
Low ICR/GIE
Low ICR/Non−GIE
Progression Free Survival probability
p = 0.00023
Time
Strata
R
NR
Progression Free Survival probability
p = 0.001
Time

Supplementary Figure 9. *IHC and clinical outcome for High ICR/GIE and High ICR/Non-GIE classified lesions.*

a Barplot of frequencies of HLA-I score (calculated by multiplying the score for staining intensity with the percentage of positive cells from IHC staining and then grouped into negative (=0), low (< median) and high (>=median)) for samples classified as High ICR/GIE (n = 5) or High ICR/Non-GIE (n = 1) at week12 (*p*-value from two-sided Pearson's chi-squared test statistic). **b** Kaplan Meier for PFS by patients classified as High ICR/GIE (n = 5) or High ICR/Non-GIE (n = 4) at week12 (first row of the panel). Kaplan Meier for OS and PFS by patients classified into ICR/GIE subtypes (High ICR/GIE n = 5, High ICR/Non-GIE n = 4, Low ICR/GIE n = 1, Low ICR/Non-GIE n = 4) at week12 (second and third rows of the panel). Kaplan Meier for PFS of R (n = 6) or NR (n = 8) patients (last row of panel). Time is indicated in months and censor points are indicated by vertical lines. Overall *p*-values are calculated by log-rank test. Source data are provided as a Source Data file.

a

Patient_ID	Timing of biopsy	Clinical response	ICR	GIE	CD8$^+$ density, tumor core (cells/mm^2)	Tumor HLA Class I Grade (0-3)	Agreement of clinical response with ICR/GIE/CD8/HLA profile	Possible interpretation
3	Week 0	R	HIGH ICR	GIE	1460	3	YES	Response associated with High ICR/GIE classification, high level of CD8$^+$ infiltrate and retained tumor HLA class I expression
3	Week 4	R	HIGH ICR	GIE	639	3		
3	Week 12	R	HIGH ICR	GIE	1257	3		
4	Week 0	R	HIGH ICR	GIE	487	3	YES	Response associated with High ICR/GIE classification, high level of CD8$^+$ infiltrate and retained tumor HLA class I expression
4	Week 4	R	LOW ICR	NON-GIE	106	3		
4	Week 12	R	HIGH ICR	GIE	912	3		
5	Week 0	R	HIGH ICR	GIE	1476	0	YES	Response associated with High ICR/GIE classification, high level of CD8$^+$ infiltrate, in spite of defective HLA class I expression
5	Week 4	R	HIGH ICR	GIE	1139	2		
5	Week 12	R	HIGH ICR	GIE	1015	1		
10	Week 0	R	HIGH ICR	GIE	263	3	YES	Response associated with High ICR/GIE classification, high level of CD8$^+$ infiltrate and retained tumor HLA class I expression
10	Week 4	R	HIGH ICR	GIE	1087	3		
10	Week 12	R	HIGH ICR	GIE	493	2		
11	Week 0	R	LOW ICR	NON-GIE	29	1	NO	Response not associated with the observed ICR/GIE/CD8/HLA profile, but explained by clinical behaviour of tumor biopsies and target lesions in a patient with SD response (see panel B)
11	Week 4	R	LOW ICR	GIE	82	2		
11	Week 12	R	LOW ICR	NON-GIE	680	NA		
1	Week 0	NR	HIGH ICR	GIE	564	1	YES	Lack of response associated with low level of CD8$^+$ infiltrate and defective tumor HLA class I expression in spite of High ICR/GIE classification
1	Week 4	NR	HIGH ICR	GIE	153	0		
1	Week 12	NR	HIGH ICR	GIE	171	2		
2	Week 0	NR	LOW ICR	NON-GIE	65	0	YES	Lack of response associated with Low ICR/Non GIE classification, low level of CD8$^+$ infiltrate and defective tumor HLA class I expression
2	Week 4	NR	LOW ICR	NON-GIE	142	1		
2	Week 12	NR	LOW ICR	NON-GIE	194	0		
6	Week 0	NR	LOW ICR	GIE	52	1	YES	Lack of response associated with Low ICR/GIE classification, low level of CD8$^+$ infiltrate and defective tumor HLA class I expression
6	Week 4	NR	LOW ICR	GIE	86	2		
7	Week 0	NR	LOW ICR	GIE	9	3	YES	Lack of response associated with Low ICR/ Non-GIE classification, low level of CD8$^+$ infiltrate, in spite of retained tumor HLA class I expression
7	Week 4	NR	HIGH ICR	GIE	NA	3		
7	Week 12	NR	LOW ICR	NON-GIE	107	3		
8	Week 0	NR	LOW ICR	NON-GIE	15	1	YES	Lack of response associated with Low ICR/ Non-GIE classification, low level of CD8$^+$ infiltrate, in spite of retained tumor HLA class I expression
8	Week 4	NR	LOW ICR	NON-GIE	107	3		
8	Week 12	NR	LOW ICR	NON-GIE	201	3		
9	Week 0	NR	HIGH ICR	NON-GIE	550	1	YES	Lack of response associated with High ICR/ Non-GIE classification, and loss of HLA class I on tumor, in spite of high level of CD8$^+$ infiltrate
9	Week 4	NR	HIGH ICR	NON-GIE	252	0		
9	Week 12	NR	HIGH ICR	NON-GIE	922	0		

b

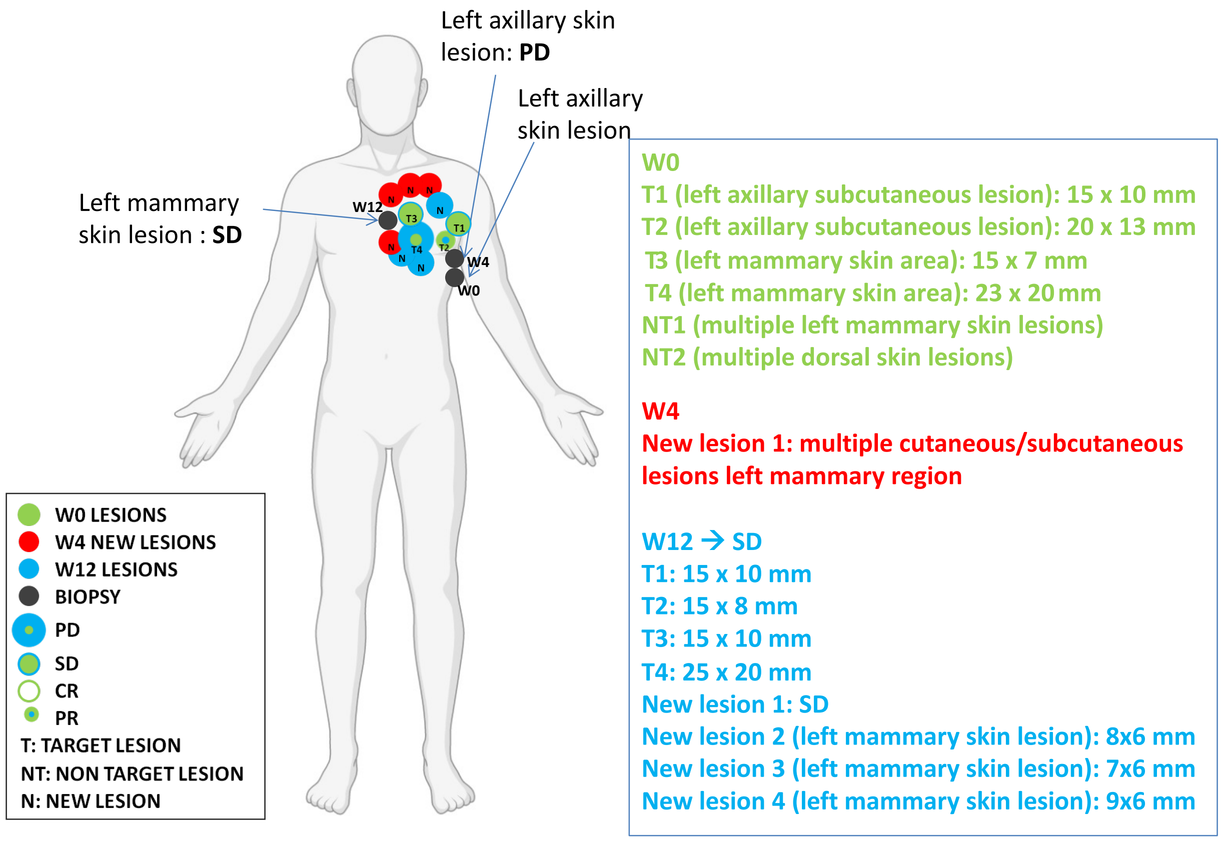

Supplementary Figure 10. *GIE characterization of individual biopsies.*

a Characterization of baseline (week 0), week 4 and week 12 tumor lesions for ICR and GIE scores, for CD8+ density in the tumor core and for level of expression of HLA Class I by IHC. **b** Localization and evolution of neoplastic lesions in patient #11. Figure created with BioRender.com.

References

1. Cabrita, R. et al. Tertiary lymphoid structures improve immunotherapy and survival in melanoma. Nature 577, 561–565 (2020).
2. Patil, N. S. et al. Intratumoral plasma cells predict outcomes to PD-L1 blockade in non-small cell lung cancer. Cancer Cell 40, 289-300.e4 (2022).
3. Cui, C. et al. Neoantigen-driven B cell and CD4 T follicular helper cell collaboration promotes anti-tumor CD8 T cell responses. Cell 184, 6101-6118.e13 (2021).
4. Zhang, Y. et al. Single-cell analyses reveal key immune cell subsets associated with response to PD-L1 blockade in triple-negative breast cancer. Cancer Cell 39, 1578-1593.e8 (2021).
5. Meylan, M. et al. Tertiary lymphoid structures generate and propagate anti-tumor antibody-producing plasma cells in renal cell cancer. Immunity 55, 527-541.e5 (2022).
6. Anichini, A. et al. Landscape of immune-related signatures induced by targeting of different epigenetic regulators in melanoma: implications for immunotherapy. J. Exp. Clin. Cancer Res. 41, 325 (2022).
7. Grasso, C. S. et al. Conserved Interferon-γ Signaling Drives Clinical Response to Immune Checkpoint Blockade Therapy in Melanoma. Cancer Cell (2020) doi:10.1016/j.ccell.2020.11.015.
8. Ayers, M. et al. IFN-γ-related mRNA profile predicts clinical response to PD-1 blockade. J. Clin. Invest. 127, 2930–2940 (2017).
9. Rodig, S. J. et al. MHC proteins confer differential sensitivity to CTLA-4 and PD-1 blockade in untreated metastatic melanoma. Sci. Transl. Med. 10, (2018).
10. Asrir, A. et al. Tumor-associated high endothelial venules mediate lymphocyte entry into tumors and predict response to PD-1 plus CTLA-4 combination immunotherapy. Cancer Cell 40, 318-334.e9 (2022).
11. Zheng, L. et al. Pan-cancer single-cell landscape of tumor-infiltrating T cells. Science 374, abe6474 (2021).
12. Robinson, M. D., McCarthy, D. J. & Smyth, G. K. edgeR: a Bioconductor package for differential expression analysis of digital gene expression data. *Bioinformatics* **26**, 139–140 (2010).
13. Ritchie, M. E. *et al.* limma powers differential expression analyses for RNA sequencing and microarray studies. *Nucleic Acids Res.* **43**, e47 (2015).
14. Wijetunga, N. A. et al. SMITE: an R/Bioconductor package that identifies network modules by integrating genomic and epigenomic information. *BMC Bioinformatics* **18**, 41 (2017).
15. Reimand, J., Kull, M., Peterson, H., Hansen, J. & Vilo, J. g:Profiler--a web-based toolset for functional profiling of gene lists from large-scale experiments. Nucleic Acids Res. 35, W193-200 (2007).

SUPPLEMENTARY NOTE
Study Protocol

Page: v.2.0
Protocol Number: NIBIT-M4
EUDRACT Number: 2015-001329-17
Date: Oct 14, 2016

Clinical Protocol

A phase 1b, open-label, dose escalation study investigating different doses of SGI-110 in combination with ipilimumab in unresectable or metastatic melanoma patients

Sponsor

Fondazione Network Italiano per la Bioimmunoterapia dei Tumori (NIBIT)
President Fondazione NIBIT
Michele Maio, MD
c/o Studio Buzzo Bernardi
Via Goffredo Mameli 3/1
16122 Genova
ITALY
Tel. +39 010 8398491/92
Tel: +39 0577-586335
Fax +39 010 8398490
Fax: +39 0577-586303

Principal Investigator Fondazione NIBIT

Anna Maria Di Giacomo, MD
Medical Oncology and Immunotherapy
Azienda Ospedaliera Universitaria Senese
Viale Bracci, 14
53100 Siena
ITALY
Tel: +39 0577-586069
Fax: +39 0577-586303

Laboratory Coordinator Fondazione NIBIT

Maresa Altomonte, MD
Medical Oncology and Immunotherapy
Azienda Ospedaliera Universitaria Senese
Viale Bracci, 14
53100 Siena
ITALY
Tel: +39 0577-586302
Fax: +39 0577-586303

Study Coordinator the Fondazione NIBIT

Giovanni Amato, PhD
Medical Oncology and Immunotherapy
Azienda Ospedaliera Universitaria Senese
Viale Bracci, 14
53100 Siena
ITALY
Tel: +39 0577-586326
Fax: +39 0577-586303

TABLE OF CONTENTS

TABLE OF CONTENTS....2
SYNOPSIS 5
1 INTRODUCTION AND STUDY RATIONALE....10
1.1 Research Hypothesis....10
1.2 Introduction....10
1.3 Investigational Products....10
1.3.1 SGI-110....10
1.3.2 SGI-110 in hematologic malignancies....12
1.3.3 Immunomodulatory activity of SGI-110 in hematologic malignancies....13
1.3.4 Immunomodulatory activity of SGI-110 in solid malignancies....14
1.3.5 Summary of Safety....14
1.3.6 Ipilimumab in Malignant Melanoma....16
1.4 Study Rationale....20
1.4.1 Rationale for Combination Therapy with SGI-110 and Ipilimumab....21
1.5 Overall Risk/Benefit Assessment....22
2 STUDY OBJECTIVES....23
2.1 Primary Objective....23
2.2 Secondary Objectives....23
2.3 Exploratory Objectives....23
3 ETHICAL CONSIDERATIONS....23
3.1 Good Clinical Practice....23
3.2 Institutional Review Board/Independent Ethics Committee....24
3.3 Informed Consent....24
4 INVESTIGATIONAL PLAN....24
4.1 Study Design and Duration....24
4.1.1 Study Phases....25
4.1.2 Tumor Assessments (TA)....27
4.1.3 Safety Assessment....27
4.1.4 Study Schema....28
4.1.5 Rationale for starting dose and for dose escalation schema....28
4.1.6 Duration of Study....29
4.2 Study Population....29
4.2.1 Inclusion Criteria....29
4.2.2 Exclusion Criteria....31
4.2.3 Discontinuation of Subjects from Treatment....32
5 TREATMENTS....33
5.1 Study Treatment....33
5.1.1 Investigational Product....33
5.1.2 Identification....34
5.1.3 Packaging and Labeling....34
5.1.4 Handling and Dispensing....34
5.2 Method of Assigning Subjects to a Treatment....36

5.2.1 Dosage and Administration 36
5.2.2 Dose Escalation 37
5.3 Dose-limiting Toxicity....... 37
5.3.1 Dose Modifications 38
5.3.2 Discontinuation Criteria 39
5.4 Concomitant Treatments....... 43
5.4.1 Antibiotics 43
5.4.2 Hematopoietic Growth Factors....... 44
5.4.3 Prohibited and/or Restricted Treatments....... 44
5.4.4 Other Restrictions and Precautions....... 44
5.5 Treatment Compliance....... 45
6 STUDY ASSESSMENTS AND PROCEDURES....... 46
6.1 Flow Chart/Time and Events Schedule 46
6.2 Study Materials....... 49
6.3 Safety Assessments....... 49
6.3.1 Medical History, Physical Exam, Physical Measurements....... 49
6.3.2 Vital Signs 50
6.3.3 Pregnancy Testing....... 50
6.3.4 ECOG Status 50
6.3.5 Adverse Events Monitoring....... 50
6.3.6 Concomitant Medications 50
6.3.7 Laboratory Test Assessments 51
6.3.8 Tumor Tissue biopsy 52
6.4 Efficacy Assessments 52
6.4.1 Primary Efficacy Assessment 52
6.4.2 Secondary Efficacy Assessment....... 52
6.4.3 Exploratory Assessment 52
6.5 Pharmacokinetic Assessments 57
6.6 Translational Research....... 57
7 ADVERSE EVENTS....... 58
7.1 Definitions 58
7.1.1 Serious Adverse Events 58
7.1.2 Non serious Adverse Events 59
7.2 Dose-limiting Toxicities 59
7.3 Assignment of Adverse Event Intensity and Relationship to Study Drug....... 60
7.4 Collection and Reporting....... 60
7.4.1 Serious Adverse Events 60
7.4.2 Handling of Expedited Safety Reports 61
7.4.3 Non serious Adverse Events 62
7.5 Laboratory Test Abnormalities....... 62
7.6 Overdose....... 63
7.7 Pregnancy 63
7.7.1 Requirements for Pregnancy Testing....... 63
7.7.2 Reporting of Pregnancy 63
7.8 Other Safety Considerations....... 64
8 STATISTICAL CONSIDERATIONS 64

8.1 Sample Size Determination 64
8.2 Populations for Analyses 65
8.3 Endpoint Definitions....66
8.3.1 Primary Endpoint....66
8.3.2 Secondary Endpoints....66
8.3.3 Exploratory Endpoints 67
8.4 Analyses....67
8.4.1 Demographics and Baseline Characteristics....67
8.4.2 Safety Analyses....67
8.4.3 Efficacy Analyses 67
8.4.4 Translational Research 68
8.5 Interim Analyses....68
9 ADMINISTRATIVE SECTION 68
9.1 Compliance....68
9.1.1 Compliance with the Protocol and Protocol Revisions 68
9.1.2 Monitoring 69
9.1.3 Investigational Site Training....69
9.2 Records Retention....70
9.2.1 Case Report Forms 70
9.2.2 Study Drug Records 71
9.3 Destruction of Study Drug....71
9.3.1 Destruction of Study Drug 71
9.4 Publications....72
ADDITIONAL ETHICAL CONSIDERATIONS 73
APPENDIX 1....73
1 INFORMED CONSENT PROCEDURES 73
1.1 Subjects Unable to Give Written Informed Consent 73
1.1.1 Minors (Not Applicable) 73
1.1.2 Subjects Experiencing Acute Events or Emergencies 74
1.1.3 Mentally Impaired or Incapacitated Subjects....74
1.1.4 Other Circumstances....74
1.1.5 Illiterate Subjects 74
1.2 Update of Informed Consent 75
LIST OF ABBREVIATIONS....77
REFERENCES 80
APPENDIX A....83
APPENDIX B 85

SYNOPSIS

Clinical Protocol NIBIT-M4

Title of Study: A phase 1b study of SGI-110 in combination with ipilimumab in unresectable or metastatic melanoma patients

Estimated Number of Study Centers and Countries/Regions: 1 NIBIT Center in Italy

Study Phase: Ib

Research Hypothesis: The research hypothesis is that SGI-110 will be able to be safely administered in combination with ipilimumab in previously treated or untreated unresectable or metastatic melanoma patients. At the same time, preliminary signals of biological and clinical activity will be collected.

Primary Objective: To assess, Maximum Tolerated Dose (MTD) and safety of SGI-110 in combination with ipilimumab in 21 day cycles in melanoma patients.

Secondary Objectives:

To evaluate the immune-related (ir) -Disease Control Rate (ir-DCR), immune-related (ir) -Objective Response Rate (ir-ORR), immune-related (ir) -Time to Response (ir-TTR) and immune-related (ir) -Duration of Response (ir-DOR).

To evaluate median immune-related (ir) Progression Free Survival (ir-PFS), median Overall Survival (OS), and survival rate at 1 and 2-years.

Exploratory Objectives:

To investigate immune-biologic correlates to treatment with SGI-110 in combination with ipilimumab (**see Appendix A**)

Characterize the pharmacokinetic profile of SGI-110 and decitabine. For a detailed workplan of pharmacoinetic analyses (**see Appendix B**)

Study Design: This is a Phase 1, dose-escalation study aims to evaluate the safety, tolerability, and Maximum Tolerated Dose (MTD) of SGI-110 in combination with ipilimumab in unresectable or metastatic melanoma patients.

All patients will undergo eligibility assessments within 28 days prior to starting study treatment. Serial safety and tumor response assessments will be performed during the study, and patients will continue

taking study treatment until documented disease progression, intolerable toxicity, withdrawal of consent, discontinuation at the discretion of the investigator or premature termination of the study.

Phase 1b, Dose Escalation: 3+3 dose escalation design.

Dose escalation will be dependent on the available toxicity information (including adverse events that are not DLTs), PK, and efficacy information, as well as the recommendations from the scientific committee

The dose escalation of SGI-110 will follow a 3+3 design. Cohorts of 3-6 patients will receive a combination therapy of Ipilimumab 3mg/kg d 1 q21 and SGI-110 according to protocol. Each patient will receive SGI-110 s.c. for 5 consecutive days (D1-5) during a 3-week cycle at the one of following doses:

- Dose Level -1: 15 mg/m^2 day
- Dose Level 0: 30 mg/m^2 day
- Dose Level +1: 45 mg/m^2 day
- Dose Level +2: 60 mg/m^2 dayIn the absence of dose-limiting toxicities (DLTs), the starting dose of SGI-110 of 30 mg/m^2 day (Dose Level 0) will be escalated to 45 mg/m^2 day on Days 1-5 (Dose Level +1). In the absence of DLT in level +1, the dose of SG1-110 of 45 mg/m^2 day will be escalated to 60 mg/m^2 day on Days 1-5 (Level +2). In the presence of 1 DLT in 3 subjects of the starting dose cohort (Dose Level 0), the cohort will enroll 3 more subjects at the same dose level. In the event that 2 or more DLTs were encountered at the starting dose cohort (Level 0), the next lower dose level of 15 mg/m^2 (Dose Level -1) will be evaluated.

The same for Dose Level +1: in the presence of 1 DLT in 3 subjects of the Dose Level +1, the cohort will enroll 3 more subjects at the same dose level. In the event that 2 or more DLTs were encountered at the Dose Level +1 cohort, the next lower dose level of 30 mg/m^2 (Dose Level 0) will be evaluated.

The MTD will be the highest SGI-110 Dose Level (15-60 mg/m^2 day on Days 1-5) in combination with ipilimumab at which no more than 1 of 6 experiences a DLT. Once the MTD is established, additional subjects will be treated at that dose up to a total of 19 subjects in the study. If no irDCR is observed the study may be terminated.

Duration of Study: We assume a planned uniform accrual period of 24 months and a follow-up period of 24 months.

Patients will be treated until Progressive Disease (PD), or confirmed PD, excessive toxicity, or patient refusal. All subjects discontinuing treatment will be followed with efficacy assessments until confirmed

PD and will be followed for survival until 2 years post Last Patient First Visit. The total study duration will be approximately 48 months.

Number of Subjects per Group: this is a Phase 1b, 3+3 dose escalation, study. Sample size will range from 6 to 19 patients.

Study Population: Subjects (men and women) ≥ 18 years old presenting with previously treated or untreated unresectable Stage III or Stage IV melanoma with measurable lesions by CT or MRI per WHO criteria, that can be amenable to biopsy. An ECOG Performance Status of 0 or 1 and adequate bone marrow, renal and liver functions are required at study entry.

Investigational Product(s), Dose and Mode of Administration, Duration of Treatment with Investigational Product(s):

SGI-110 is considered as Investigational Medicinal Product (IMP) and will be supplied by Astex and dispensed by Fondazione NIBIT /CRO. Ipilimumab will be utilized in the study according to the approved dose and schedule of administration for metastatic melanoma.

Dose and Schedule:

Phase 1b, Dose Escalation: 3+3 dose escalation.

SGI-110: start at 30 mg/m^2 s.c.day (Dose Level +1) on W0, 3, 6, 9 Day 1 - 5 q21 days. Dose Level -1): 15 mg/m^2 day; Dose Level +1: 45 mg/m^2 day; Dose Level +2: 60 mg/m^2 day.

Ipilimumab: 3 mg/Kg i.v. over 90 minutes on W1, 4, 7 and 10 for a total of 4 cycles.

During dose escalation, subjects will be allocated to 1 of 3 dose levels of SGI-110 in combination with ipilimumab 3 mg/Kg according to a classical, dose-escalation 3 + 3 design (see Figure 1). Treatment within the 4 SGI-110 Dose Levels will proceed in sequence provided there are no DLTs (see Section 5.3). Details of this evaluation and the thresholds for toxicity and dose escalation are described fully in Sections 5.3.

Duration of treatment:

Treatment with SGI-110 in combination with ipilimumab will be given for 4 doses, or until stopped due to excessive toxicity or patient’s refusal.

Tumor Assessment:

Tumor assessment with radiographic imaging (e.g., MRI of brain, CT of chest, abdomen, pelvis, other soft tissues) and digital photographs of skin lesions will be performed for all subjects at Screening, W12,

18, 24 and then every 12 weeks, for all non-progressing subjects. At each evaluation investigators will assess the overall response (complete response (CR), partial response (PR), stable disease (SD) or Progressive Disease (PD) as per ir-RC.

Safety Assessment:

The analysis of safety will be based on the frequency of the adverse events and their severity for all treated patients. Worst toxicity grades *per* patient will be tabulated for adverse events and laboratory measurements using the NCI CTCAE v.4.0.

The analysis of safety will be based on the frequency of the adverse events and their severity for all treated patients. Worst toxicity grades per patient will be tabulated for adverse events and laboratory measurements using the NCI CTCAE v.4.0. The risk/benefit relationship will be assessed.

Health-related quality of life:

Quality of life will be assessed as measured by the European Organization for Research and Treatment of Cancer Quality of Life questionnaire (EORTC):

- EORTC QLQ C-30 Questionnaire Version 3

- EORTC QLQ BN-20

Statistical Methods, Sample Size Considerations and Statistical Analyses:

The phase 1 of SGI-110 in combination with ipilimumab is designed as a dose-escalation study. The primary end-point will be the, Maximum Tolerated Dose (MTD) and safety of the combination of SGI-110 plus ipilimumab.

Six patients will be treated at the first dose level for one cycle.

In the absence of dose-limiting toxicities (DLTs), the starting dose of SGI-110 of 30 mg/m^2 day (Dose Level 0) will be escalated to 45 mg/m^2 day (Dose Level +1) on Days 1-5. In the absence of DLT in Dose Level +1, the dose of SG1-110 of 45 mg/m^2 day (Dose Level +1) will be escalated to 60 mg/m^2 day (Dose Level +2)) on Days 1-5 (Level +2). In the presence of 1 DLT in 3 subjects of the single dose cohort, the cohort will enroll 3 more subjects at the same dose level. In the event that 2 or more DLTs were encountered at the single dose cohort, the previous lower dose level will be evaluated. The MTD will be the highest SGI-110 Dose Level (15-60 mg/m^2 day on Days 1-5) in combination with ipilimumab at which no more than 1 of 6 patients experiences a DLT. Once the MTD is established, additional subjects will be treated at that dose up to a total of 19 subjects in the study. If no irDCR is observed the study may be terminated.

DLT is defined as any of the following events occurring during the first treatment cycle and clearly related to study treatment: grade 4 neutropenia (absolute granulocyte count < 0.5 x 109 /L, ≥ 5 days), febrile neutropenia grade ≥ 3 (absolute granulocyte count < 1.0 x 109 /L and fever ≥ 38.5° C), platelet count < 25.000/L or thrombocytopenic bleeding, AST or ALT grade ≥ 3 for 7 days, any grade 3 or 4 non-hematologic toxicity (excluding alopecia, non pre-medicated nausea and vomiting), grade 3 or 4 clinically significant nausea, vomiting, or diarrhoea in the presence of maximal supportive care, a required interruption of treatment > 2 weeks due to toxicity. If the toxicity fails to resolve to ≤ grade 2 with 14 days off treatment, the patient will be removed from the study.

No formal sample size calculation was done. Sample size will range from 6 to 19 patients.

Toxicity will be reported according to National Cancer Institute-Common Toxicity Criteria (NCI CTC) version 4.0 (2004) criteria. The safety analysis population will include all subjects who receive at least one dose of drug. Subjects will be assigned to treatment groups as treated. In tables showing the overall incidence of AEs, subjects who experienced the same event on more than one occasion are counted only once in the calculation of the event frequency.

All safety parameters will be summarized and presented in tables based on this safety population.

Demographic and baseline characteristics will be summarized for all randomized subjects using descriptive statistics. Reporting of safety, extent of exposure, concomitant medications and discontinuation of study therapy will be based on all treated subjects. Worst toxicity grades per subject will be tabulated for AEs and on-study laboratory measurements by using the National Cancer Institute (NCI) Common Terminology Criteria for Adverse Events (CTCAE) version 4.0.

Translational studies:

For a detailed work plan of translational studies see Appendix A.

1 INTRODUCTION AND STUDY RATIONALE

1.1 Research Hypothesis

The research hypothesis is that SGI-110 will be able to be safely administered in combination with ipilimumab in previously treated or untreated unresectable or metastatic melanoma patients. At the same time, preliminary signals of biological and clinical activity will be collected.

1.2 Introduction

Epigenetic alterations play a pivotal role in cancer development and progression. Pharmacologic reversion of such alterations is feasible, and second generation “epigenetic drugs” are in development and have demonstrated to possess significant immunomodulatory properties. This knowledge, together with the availability of new and highly effective immuno-therapeutic agents including immune check-point(s) blocking monoclonal antibodies, allows us to plan for highly innovative proof-of-principle combination studies that will likely open the path to more effective anti-cancer therapies.

Targeting immune check-point(s) with immunomodulatory mAb is a novel and rapidly evolving strategy to treat cancer, that is rapidly spreading to different tumor histologies. The prototype approach of this therapeutic modality relies on the inhibition of negative signals delivered by CTLA-4 expressed on T lymphocytes. CTLA-4 blockade has profoundly changed the therapeutic landscape of metastatic melanoma (MM), significantly improving the survival of MM patients; however, objective clinical responses are limited, and only a minority of patients achieves long-term disease control.[1] Therefore, several combination approaches are being explored to improve the efficacy of CTLA-4 blockade.[2] Along this line, based on the preclinical evidence we gained on the broad immunomodulatory activity of SGI-110, the exploratory phase 1 combination study NIBIT-M4 has been designed to provide proof-of-concept evidence to the immunologic and clinical efficacy of CTLA-4 blockade combined with DHA. Progressing Stage III or Stage IV MM patients, amenable to serial tumor biopsies will be enrolled in the study.

1.3 Investigational Products

1.3.1 SGI-110

SGI-110 (2'-deoxy-5-azacytidylyl-(3'→5')-2'-deoxyguanosine sodium salt) is a dinucleotide of decitabine and deoxyguanosine linked with a natural phosphodiester linkage. It is a potent inhibitor of DNA methylation (Griffiths, Choy et al 2013). The FDA- and EMA-approved drug, decitabine, is the active

metabolite of SGI-110. Decitabine for [IV] injection (Dacogen®) is marketed in the United States (US) for the treatment of patients with myelodysplastic syndromes (MDS) (Dacogen® Prescribing Information), and in the European Union for the treatment of elderly patients with acute myelogenous leukemia (AML) not fit for induction chemotherapy.

Decitabine is approved in more than 35 countries for MDS. Unlike decitabine, SGI-110 is resistant to deamination by cytidine deaminases. SGI-110 is cleaved by intra- and extracellular phosphodiesterases/phosphorylases releasing decitabine and deoxyguanosine. This in vivo cleavage results in gradual release of decitabine both extra and intracellularly, lengthens decitabine half-life, and hence prolongs effective exposure to decitabine. Since decitabine activity is S-phase dependent, more prolonged exposure should result in more S-phase cancer cells exposed to decitabine. Correspondingly, in vitro and in vivo studies have shown that SGI- 110 has potent antitumorigenic activity. Hence, improved decitabine pharmacokinetics (PK) after subcutaneous (SC) SGI-110 administration, relative to intravenous (IV) decitabine, may translate into better activity and/or safety of SGI-110.

SGI-110 has been developed for SC administration in a non-aqueous formulation to ensure stability. SGI-110 (and decitabine) reverses aberrant DNA hypermethylation by inhibiting DNA methyl transferase (DNMT) enzymes. Hypermethylation of cytosine-phosphate-guanine (CpG) rich regions (CpG islands) is a physiologic mechanism of permanent gene inactivation that is usurped by leukemic cells, which use it to silence tumor suppressor genes and related proteins[3]. Decitabine is a cytidine analog that profoundly inhibits DNA methylation in vitro, resulting in re-expression of previously silenced genes.[4] Decitabine appears to have a dual action on neoplastic cells. By incorporating into DNA and forming covalent bonds with DNMT, decitabine effectively depletes the cells of methylating enzymes.[4] This enzyme deficiency renders the cell unable to maintain DNA methylation after cellular replication, resulting in effective hypomethylation. However, the requirement for cell division to achieve hypomethylation and the short half-life of decitabine suggest that prolonged exposure is preferable for achieving a differentiation effect[4]. At high doses, covalently trapped DNMT acts as a bulky DNA adduct and results in cytotoxicity rather than hypomethylation.

SGI-110 is being evaluated in subjects with hematological malignancies (MDS and AML) and solid tumors (ovarian cancer, hepatocellular carcinoma [HCC]). Based on preliminary results, long interspersed nucleotide element-1 (LINE-1) demethylation was demonstrated in AML/MDS subjects treated with daily and weekly regimens. Clinical responses in subjects with AML and MDS have been observed in the ongoing Phase 1-2 study (SGI-110-01).

1.3.2 *SGI-110 in hematologic malignancies*

The promising results obtained during the pre-clinical characterization of SGI-110 prompted initiation of the first-in-human phase1/2 study in December 2010 in patients with intermediate or high risk myelodysplastic syndrome (MDS) or acute myelogenous leukemia (AML). The study (NCT01261312) was designed to have 2 segments: a dose escalation to establish the MTD and the Biologically Effective Dose (BED) based on LINE-1 demethylation relative to baseline in blood DNA. The BED was defined as the minimum dose that achieves maximal LINE-1 demethylation from 3 successive cohorts. LINE-1 demethylation provides a reliable pharmacodynamic marker and is a surrogate of global hypomethylation by hypomethylating agents (HMAs).[5] The phase 1 study explored multiple regimens. First, the study randomly assigned subjects to 28-day regimens of SGI-110 of (1) once daily for the first 5 days (Daily ×5) or (2) once weekly for 3 weeks with a rest week. Subsequently, a third 28-day regimen was studied: twice weekly SGI 110 for 3 weeks. Overall 93 patients were enrolled in seven cohorts and preliminary results were presented at ASH in December 2012.[6] The PK profile demonstrated efficient conversion of SGI-110 to decitabine as predicted from the pre-clinical data. At an SGI-110 daily dose range of 60-90 mg/m^2, the observed 5-AZA-CdR AUCs reached or exceeded the therapeutic range seen with IV 5-AZA-CdR (20 mg/m^2) with a lower Cmax, but a longer effective half-life and exposure window. Dose-related increases in LINE-1 demethylation after SGI-110 dosing was observed in the majority of subjects treated with the Daily x5 regimen between 18 and 60 mg/m^2 daily doses. Maximum average demethylation (~25%) plateaued after 60 mg/m^2, with daily doses (90-125 mg/m^2) showing no further increase. Therefore the BED for the Daily x5 regimen was established at 60 mg/m^2. This compares favourably to historical controls of patients treated with 5-AZA-CdR IV at a dose of 20 mg/m^2 Daily x5.[7] Average maximal demethylation in the Once Weekly regimen was much less potent in all cohorts compared with the Daily x5 regimen. Daily ×5 MTD was reached for MDS subjects at 90 mg/m^2/day but was not reached for AML subjects up to 125 mg/m^2. Clinical responses were observed in this heavily pre-treated patient AML and MDS population, including patients who had failed prior HMA therapies. Subsequently, a third 28-day regimen was studied: twice weekly SGI 110 for 3 weeks. The average maximum LINE-1 demethylation after 60 or 90 mg/m^2 twice weekly reached a plateau of ~18% from baseline at Day 15 and the recovery was not entirely completed by Day 28.[8] The daily regimen was selected for the dose expansion segment as it achieved the most potent LINE-1 demethylation compared to the Once Weekly and Twice Weekly regimens.

Phase 2 dose expansion was opened as a multicenter, open-label, randomized dose-response comparison of 60 *vs* 90 mg/m^2 SGI-110 SQ Daily ×5. Subjects were stratified by disease type (treatment naïve elderly AML, relapsed/refractory AML, treatment-naïve MDS, and relapsed/refractory MDS). Thirty to 50

subjects were planned for each disease cohort. The 60 mg/m^2 Daily×5 dose was chosen because it represented the BED, while the 90 mg/m^2 dose was chosen as an attempt to explore benefit from a higher dose that was still well tolerated in both MDS and AML subjects. The preliminary data for the treatment naïve AML patients were presented at ASH in December 2013. LINE-1 methylation data before and after treatment were available in 48 patients (94.1%). Average maximum LINE-1 demethylation was similar for the 60 mg/m^2 and 90 mg/m^2 dose groups (18.6% for 60 mg/m^2 and 21.4% for 90 mg/m^2). OCR for both doses combined was achieved in 28 of 51 subjects (17 CR, 11 CRi, no CRp) or 55%. There was no observed correlation between extent of LINE-1 demethylation and clinical response.[9,10]

A protocol amendment was introduced to treat an additional cohort of relapsed/refractory AML patients with a 10-day regimen at the 60 mg/m^2 dose level (Days 1-5 and 8-12 every 28 days). Preliminary data presented at ASCO 2013[11] showed that LINE-1 demethylation was more profound and sustained in subjects receiving the 10-day regimen than in those receiving the Daily ×5 regimen, based on relapsed/refractory AML subjects who received 60 mg/m^2/day SGI-110.

1.3.3 Immunomodulatory activity of SGI-110 in hematologic malignancies

The immunomodulatory activity of SGI-110 has been investigated in AML and MDS patients enrolled in the dose escalation study segment of the first-in-human phase1/2 study NCT01261312.[12,13,14] Bisulfite-modified blood DNA was analyzed by quantitative methylation-specific PCR for pre and post-treatment methylation on the promoters of CTA genes, NY-ESO-1 and MAGE-A1. The extent of demethylation of these genes was higher in patients treated with the Daily x5 schedule than in the weekly x3 schedule. A dose-dependent increase in post-treatment demethylation was observed in all the cohorts up to 125 mg/m^2; the most potent average gene promoter demethylation (14% for NY-ESO-1 and 16.3% for MAGE-A1) was observed in patients treated at 125 mg/m^2 Daily x5. Duration of gene promoter demethylation for NY-ESO-1 and MAGE-A1 was very similar to the duration observed for LINE-1 with a nadir on day 8 and an almost complete re-methylation by day 28. SGI-110-induced gene promoter demethylation resulted overall in the up-regulation or *de novo* induction of NY-ESO-1, MAGE-A1 and MAGE-A3 expression. Total RNA from blood of 23 patients treated with 36- 125 mg/m^2 SGI-110 Daily x5 was available for analysis; *de novo* induction or up-regulation of NY-ESO-1, MAGE-A1 or MAGE-A3 expression was observed respectively in 14, 7 and 11 patients. These changes in CTA expression are directly associated to a dose-dependent reduction of CTA promoter methylation, supporting a direct role of pharmacologic DNA demethylation in the observed modulation of the tumor immune profile.[13, 14]

1.3.4 Immunomodulatory activity of SGI-110 in solid malignancies

Based on its improved properties as compared to 5-AZA-CdR, the immunomodulatory properties of SGI-110 were investigated also in human melanoma xenografts. Three doses of SGI-110, administered in two distinct SQ regimens (i.e., Daily x5 and once weekly x3) were utilized to identify the optimal schedule that efficiently modified the immune profile of cancer cells with the lowest systemic toxicity. RT-PCR, quantitative RT-PCR and flow cytometry analyses demonstrated that SGI-110 induced or up-regulated the expression of different CTA in melanoma xenografts, this effect was found to be sustained by promoter methylation of CTA genes. A dose-dependent up-regulation of HLA class I antigens and of co-stimulatory molecules was also detected on neoplastic cells from SGI-treated xenografts.[14,15].

These new findings obtained with SGI-110 strongly enforced the notion that DHA represent a broad category of immunomodulatory compounds to be explored in novel combination regimens with up-coming immunotherapeutic agents. Thus, subsequent *in vivo* studies investigated the anti-tumor effect(s) of SGI-110 combined with the murine monoclonal antibody (mAb) 9H10 directed to cytotoxic T lymphocyte antigen-4 (CTLA-4) on mammary carcinoma cells TS/A grafted into BALB/c mice. SGI-110 was administered to mice at 3mg/kg Daily x5, alone or in combination with 100 µg of mAb 9H10, in a concomitant or subsequent schedule. Unlike the concomitant regimen, a significant anti-tumor effect was achieved in mice treated with SGI-110 followed by mAb 9H10, with a tumor growth inhibition of 84.4% compared to control animals. Notably, SGI-110-based therapeutic regimens induced the expression of the murine CTA P1A and Mage-a family in tumor tissues. Supporting the contribution of immune effector mechanism(s) in the anti-tumor activity of the combination regimen obtained in immunocompetent mice, the addition of mAb 9H10 did not improve the anti-tumor effect of SGI-110 administered alone in immunocompromised athymic nude and SCID-Beige mice. [14,16]

These comprehensive evidence generated with SGI-110 *in vivo* demonstrated that besides having a direct activity on tumor growth as a single agent, SGI-110 was able to "sensitize" neoplastic cells to the anti-tumor activity of CTLA-4 blockade, providing a sound scientific rationale to develop new immunotherapeutic approaches combining SGI-110 with therapeutic mAb to immune check-points.

1.3.5 Summary of Safety

Overall, most Grade 3 or higher AEs occurred in the Blood and Lymphatic System Disorders SOC. In Studies SGI-110-01, SGI-110-03, and SGI-110-02 the incidence of Grade 3 or higher AEs in the Blood and Lymphatic System SOC was 76% (272/357 subjects), 87.2% (41/47 subjects), and 68% (46/68 subjects), respectively.

1.3.5.1 Monotherapy

Overall in Study SGI-110-01 (SGI-110 monotherapy in treatment-naïve and relapsed/refractory AML/MDS subjects [N=357]), the AEs with highest incidence were injection site AEs (48.2%), febrile neutropenia (47.1%), diarrhea (38.7%), thrombocytopenia (37.3%), anemia (36.1%), fatigue (35.6%), and nausea (34.2%). The related AEs with the highest incidence in this study were injection site AEs (47.1%), thrombocytopenia (28.3%), anemia (26.1%), and neutropenia (25.2%). Bone marrow suppression appears to be a consequence of the underlying disease (AML/MDS) combined with a myelosuppressive effect of SGI-110 treatment. In Study SGI-110-01 (N=357 AML/MDS subjects) other notable reported AEs included febrile neutropenia, pneumonia, and mucositis/stomatitis.[17]

In SGI-110-03 (SGI-110 monotherapy in subjects [N=47] with advanced HCC), the AEs with the highest incidence (Stage 1 and Stage 2 combined) were neutropenia (78.7%), injection site AEs (48.9%), thrombocytopenia (40.4%), fatigue (38.3%), and leukopenia (36.2%) (Clinical Database: Table 14.6.4 and Table 14.6.14). The related AEs with the highest incidence in this study included neutropenia (74.5%), injection site AEs (48.9%), leukopenia (36.2%). In this study the SGI-110 was administered in 28 day cycles as a single agent and the safe dose was found to be 45 mg/m^2.

SGI-110 has been explored in a variety of doses and schedules. Little data exists for cycles less than 28 days; however, as ipilimumab is approved for 4 administrations every three weeks this protocol is exploring a three-week cycle of SGI-110. Recovery of myelosuppression is of substantial interest and will likely determine the safe dose. Ipilimumab has no myelosuppressive qualities and the toxicity of SGI-110 will determine its dose in all likelihood.

1.3.5.2 Combination Therapy

In SGI-110-02 (45 or 30 mg/m^2 SGI-110 in combination with carboplatin IV AUC 5 or 4 in subjects with platinum-resistant recurrent ovarian cancer), the AEs with highest incidence (Stage 1 and 2 combined [N=68]) were neutropenia (60.3%), nausea (60.3%), fatigue (55.9%), injection site AEs (44.1%), vomiting (44.1%), constipation (38.2%), anemia (36.8%), and abdominal pain (35.3%). The related AEs with highest incidence (Stage 1 and 2 combined [N=68]) were neutropenia (55.9%), nausea (44.1%), injection site AEs (36.8%), anemia (33.8%), and fatigue (29.4%). The myelosuppressive effects seen in this study occurred at a rate that would be expected with carboplatin AUC 5 alone or AUC 4 alone.

De-escalation to 30 mg/m^2 was necessary in this trial.

1.3.5.3 Overdose

No event associated with overdose has been reported in any SGI-110 study to date. Standard supportive measures should be taken in the event of an overdose.

1.3.6 Ipilimumab in Malignant Melanoma

1.3.6.1 Clinical Pharmacology Summary: ipilimumab monotherapy

Ipilimumab has a terminal half-life of approximately 15.4 days. The expected in vivo degradation of monoclonal antibodies is to small peptides and amino acids via biochemical pathways that are independent of cytochrome P450 enzymes. The population PK of ipilimumab was studied with 785 subjects and demonstrated that PK of ipilimumab is linear and exposures are dose proportional across the tested dose range of 0.3 to 10 mg/kg, and the model parameters are time invariant. Upon repeated dosing of ipilimumab, administered every three weeks, minimal systemic accumulation was observed by an accumulation index of 1.5-fold or less and ipilimumab steady-state concentrations were achieved by the third dose. The ipilimumab clearance of 16.8 mL/h from population PK analysis is consistent with that determined by PK analysis. The terminal half-life (T-HALF) and Vss of ipilimumab calculated from the model were 15.4 days, and 7.47 L, which are consistent with that determined by non-compartmental analysis (NCA). Volume of central (Vc) and peripheral compartment were found to be 4.35 L and 3.28 L, respectively, suggesting that ipilimumab first distributes into plasma volume and subsequently into extracellular fluid space. Clearance of ipilimumab and Vc were found to increase with increase in body weight. Nevertheless, there was no significant increase in exposure with increase in body weight when dosed on a mg/kg basis, supporting dosing of ipilimumab based on a weight normalized regimen. Additional details are provided in investigator brochure.[18]

1.3.6.2 Clinical Efficacy to Date

Ipilimumab Monotherapy

Overall survival and other efficacy endpoints were assessed in ipilimumab studies.

- OS: Ipilimumab (at 3 and 10 mg/kg) prolongs survival in subjects with advanced melanoma (previously treated with 3 mg/kg monotherapy and previously untreated with 10 mg/kg ipilimumab + DTIC)[1,19]
- Best Objective Response Rate (BORR): By the conventional mWHO criteria confirmed objective responses have been observed in subjects receiving ipilimumab. These responses tend to be

durable with the median duration of responses of 11.47 months (3 mg/kg ipilimumab + gp100) and 19.3 months.

- Disease control rate (DCR): Disease stabilization in subjects receiving ipilimumab monotherapy is a key characteristic of anti-tumor activity. Durable stable disease (SD) or a slow steady decline of tumor lesion size over time has been observed. Consequently, SD, as well as objective responses, (both captured in DCR) are important for completely characterizing anti-tumor activity of ipilimumab.
- PFS: Statistically significant results in favor of ipilimumab-treated groups were observed for PFS indicating consistency of efficacy results in 2 Phase 3 trials.
- Exploratory efficacy endpoints: Four distinct response patterns have been observed following ipilimumab monotherapy.
- Shrinkage in baseline lesions, without new lesions.
- Durable SD (in some patients followed by a slow, steady decline in total tumor burden).
- Response after an increase in total tumor burden.
- Response in the presence of new lesions.

Based on these observations, subjects should receive the entire induction regimen (4 doses) as tolerated, regardless of the appearance of new lesions or growth of existing lesions during induction. Assessments of tumor response should be conducted only after completion of induction therapy and, if possible, confirmed.

In melanoma, ipilimumab prolonged survival in subjects with pre-treated and previously untreated, advanced melanoma, based on results from 2 large, multinational, double-blind, Phase 3 studies (MDX010-20 and CA184024), supported by data from key Phase 2 studies.

The Phase 3 study MDX010-20 has demonstrated a clinically meaningful and statistically significant survival benefit in pre-treated advanced melanoma. The study compared the overall survival (OS) of ipilimumab plus a melanoma-specific vaccine (gp100) to that of gp100 alone. A second comparison defined the OS of ipilimumab alone vs. gp100 alone. Both comparisons demonstrated statistically significant improvements in OS ($p = 0.0004$ and 0.0026, respectively). The 1-year survival for the two ipilimumab-containing groups, respectively, was 44% and 46% respectively, compared to 25% for the gp100 control group. The 2-year survival was 22%, 24% and 14% respectively. The median survival was

10, 10.1, and 6.4 months, for ipilimumab plus gp100, ipilimumab monotherapy, and gp100 monotherapy, respectively.

In MDX010-20, all response-related endpoints (BORR, DCR, and PFS) showed consistent, positive results for the ipilimumab-containing groups relative to the gp100 group. Response duration was longer than 2 years in 60.0% (9/15) of responders in the ipilimumab monotherapy group (range: 26.5+ to 44.2+ months) and 17.4% (4/23) of responders in the ipilimumab + gp100 group (range: 27.9+ to 44.4+ months). None of the subjects treated with gp100 remained in response at the 2-year time point. The development or maintenance of objective response or disease control following ipilimumab treatment in MDX010-20 was similar with or without the use of systemic corticosteroids.

The primary objective of CA184-024 study was the comparison of OS in subjects administered ipilimumab (10 mg/kg) + DTIC vs DTIC monotherapy. The HR for comparison of OS between the groups was 0.72 (95% CI: 0.59, 0.87; P = 0.0009), indicating a 28% risk reduction in OS for the ipilimumab + DTIC group compared with the DTIC monotherapy group. The median OS was 11.2 months (95% CI: 9.4, 13.6) in the ipilimumab + DTIC group and 9.1 months (95% CI: 7.8, 10.5) in the DTIC monotherapy group. The Kaplan-Meier survival curves are similar for the groups through approximately the first 4 months of treatment, after which a separation in the curves suggests a favorable OS advantage for the ipilimumab + DTIC group. The long-term survival effect of ipilimumab is reflected in the estimated survival rates, which were consistently higher for the ipilimumab + DTIC group relative to the DTIC monotherapy group. In the ipilimumab + DTIC group, the median duration of response (complete response/partial response [CR/PR]) using mWHO criteria was 19.3 months, and the median duration of SD was 4.7 months, as determined by the Independent Review Committee (IRC). In the DTIC monotherapy group, the median duration of response was 8.1 months and the median duration of stable disease was 4.6 months. The overall median durations of response were the same as for those subjects who received maintenance dosing.

1.3.6.3 Summary of Safety

Ipilimumab Monotherapy [17]

Advanced Melanoma - ipilimumab 3 mg/kg

The safety profile of ipilimumab at 3 mg/kg in the Phase 3 study MDX010-20 was consistent with that observed at 3 mg/kg in the Phase 2 studies. The most common treatment-related AEs in subjects who received ipilimumab 3 mg/kg were irAEs affecting the skin and GI tract (eg, pruritus, rash, and diarrhea).

The majority of these events were Grade 1 to 2 in severity. The most common Grade 3 to 4 treatment-related AEs were GI irAEs of colitis and diarrhea (reported in 3% to 5% of subjects).

Diarrhea and colitis were consistently among the most common treatment-related AEs during the induction phase leading to discontinuation of ipilimumab. In MDX010-20, treatment-related AEs leading to discontinuation were reported in 9.9%, 6.8%, and 3.0% of the subjects in the ipilimumab monotherapy, ipilimumab plus gp100, and gp100 monotherapy groups, respectively. The most common (> 1% of subjects in either the ipilimumab monotherapy group or ipilimumab plus gp100 groups) treatment-related AEs leading to discontinuation in the ipilimumab groups were colitis (2.3% and 2.4%), diarrhea (1.5% and 2.6%), and uveitis (1.5% and 0%). In the pooled 3 mg/kg group from the Phase 2 studies, 8.1% of subjects reported treatment related AEs leading to discontinuation, the most common were hypopituitarism (2.7%), colitis (1.8%), and decreased appetite (1.8%).

Diarrhea and colitis were consistently the most common treatment-related SAEs reported for subjects receiving ipilimumab across studies. In MDX010-20, treatment-related SAEs were reported in 16.8%, 12.6%, and 3.8% of the subjects in the ipilimumab monotherapy, ipilimumab plus gp100, and gp100 monotherapy groups, respectively. The most common (> 1% of subjects in either the ipilimumab monotherapy group or ipilimumab plus gp100 groups) treatment-related SAEs in the ipilimumab groups were colitis (5.3% and 3.4%), diarrhea (3.8% and 3.4%), hypophysitis (1.5% and 0.3%), hypopituitarism (1.5% and 0.8%), renal failure (1.5% and 0%), hypotension (1.5% and 0%), and pyrexia (0% and 1.1%). In the pooled 3 mg/kg group from the Phase 2 studies, 17.1% of subjects reported treatment related SAEs; the most common were colitis (4.5%), diarrhea (4.5%), hypopituitarism (2.7%), and pyrexia (2.7%).

Across the 3 groups in MDX010-20 and the pooled 3 mg/kg group from the Phase 2 studies, approximately 20% to 30% of subjects died during the induction phase.70 Most (> 90%) of the deaths were due to disease progression or other causes considered unrelated to study drug. In MDX010-20, treatment-related deaths (defined as a treatment-related AE with an outcome of death, reported at any time during the study) were reported in 4 subjects (3.1%), 8 subjects (2.1%), and 2 (1.5%) subjects in the ipilimumab monotherapy, ipilimumab plus gp100, and gp100 monotherapy groups, respectively.70 Of the 12 treatment-related deaths in the 2 ipilimumab-containing groups, 7 were associated with an irAE (GI perforation [4], colitis [1], liver failure [1], and Guillain-Barré Syndrome [1]). In addition, 1 subject in the ipilimumab plus gp100 group of MDX010-20 had a Grade 4 skin irAE (Stevens Johnson syndrome/toxic epidermal necrolysis/Lyell's syndrome), but died due to a treatment-related acute respiratory distress syndrome, which is not considered to be an irAE. In the pooled group from the Phase 2 studies, 2 subjects (1.8%) experienced treatment-related deaths, 1 of which was associated with an irAE (GI perforation).

1.4 Study Rationale

Metastatic melanoma is an aggressive tumor associated with very poor prognosis. The median survival of patients with advanced or metastatic disease is in the range of 6 months, mainly depending on location and number of metastases and patient performance status.

Until recently, medical treatment for metastatic melanoma has been highly unsuccessful with no impact on patients' survival. However, the increasing knowledge of melanoma immune-biology has prompted studies to identify new therapeutic strategies able to generate more effective host's immune responses against neoplastic cells. Among these, targeting of cytotoxic T-lymphocyte antigen-4 (CTLA-4), a key immune checkpoint molecule, by the monoclonal antibody (mAb) designated ipilimumab has emerged as the therapeutic prototype of immunomodulating mAb.

Ipilimumab is a fully human mAb (IgG1k) with high affinity for CTLA-4 molecule that blocks the binding of CTLA-4 to its receptor B7, thereby preventing down-regulation of the immune response. Ipilimumab increases the proliferation of activated T cells and potentiates the interaction between T cells and cancer cells. Ipilimumab has been approved by FDA and EMA for the treatment of metastatic melanoma since in two pivotal trials it significantly improved the survival of subjects with advanced melanoma (unresectable Stage III or Stage IV).[1,19,20]

In spite of the limited number of objective responses (~15%) observed across a spectrum of doses and schedules of administration, in treatment-naïve and pre-treated patients, about 20% of subjects experienced a long-term survival. The latter was observed in phase II/III studies, and in the daily practice as demonstrated by two large Expanded Access Programs; noteworthy, survival rates plateaued at 3 years.[21,22]

These highly encouraging results have provided the basis to identify new therapeutic strategies to further improve the clinical efficacy of ipilimumab in melanoma patients, and more broadly in subjects with solid and hematologic malignancies. Therefore, various combinations of ipilimumab with other immune-modulating, antiangiogenic, or chemotherapeutic or targeted agents are being tested, and are beginning to demonstrate the potential for synergy.[2,23] In this setting, the identification of effective combination(s) of ipilimumab with novel immunomodulatory agents is particularly intriguing.

Extensive in vitro and in vivo studies focused on human melanoma, as well as several other tumor histotypes, allowed us to demonstrate a striking immunomodulatory activity of the DNA hypomethylating agent (DHA) 5-aza-2'-deoxycitidine (5-AZA-CdR), (for review see Sigalotti et al.).[21] This bulk of experimental evidences led us to hypothesize that 5-AZA-CdR could represent a useful drug to combine with immunotherapeutic agents[25], including ipilimumab[26], to develop novel chemo-immunotherapeutic

approaches for the treatment of melanoma patients and, more broadly, of subjects with solid and hemopoietic malignancies.

Most recently, we have demonstrated that the second-generation DNA hypomethylating agent SGI-110 also holds a strong immunomodulatory activity. SGI-110 induced/up-regulated the expression of different cancer/testis antigens (CTA) (i.e., MAGE-A1, MAGE-A2, MAGE-A3, MAGE-A4, MAGE-A10, GAGE 1-2, GAGE 1-6, NY-ESO-1, and SSX 1-5) in cancer cell lines of different histotype, both at mRNA and at protein levels. Moreover, quantitative methylation-specific PCR (qMSP) analyses identified a hypomethylation of MAGE-A1 and NY-ESO-1 promoters in SGI-110-treated melanoma cells, supporting the direct role of pharmacologic DNA demethylation in CTA induction. SGI-110 also up-regulated the expression of HLA class I antigens and of the accessory molecule ICAM-1, resulting in an improved recognition of cancer cells by gp100-specific cytotoxic T lymphocytes.[27]

Consistent with these in vitro observations, subsequent in vivo studies with human melanoma xenografts excised from SGI-110-treated mice allowed us to demonstrate a direct correlation between promoter demethylation and induction/up-regulation of the expression of different CTA in tumor tissues.[15]

Additional data supporting the immunomodulatory activity of SGI-110 derived from the analysis of MDS or AML patients enrolled in a randomized phase 1-2 study in which the hypomethylating activity of SGI-110 on promoters of specific CTA, and the induction/up-regulation of their expression was tested. qMSP analyses showed that SGI-110 was able to reduce the constitutive methylation levels of NY-ESO-1 and MAGE-A1 promoters and to induce/up-regulate the expression of NY-ESO-1, MAGE-A1, and MAGE-A3 in treated patients.[13]

Consistent with our previous observations with 5-AZA-CdR10, SGI-110 significantly improved the therapeutic efficacy of an anti-CTLA-4 mAb in a syngeneic murine mammary carcinoma model. The best antitumor effect was achieved in mice treated with SGI-110 followed by the anti-CTLA-4 mAb. Noteworthy, no body weight loss was observed in investigated mice suggesting for a good tolerability of the therapeutic combination.[28]

Altogether, the evidence above provide a strong pre-clinical rationale to design novel trials combining DHA and immunotherapeutic agents; among these, the combination between DHA and anti-CTLA4 blocking mAb appears of particular interest to be explored.

1.4.1 Rationale for Combination Therapy with SGI-110 and Ipilimumab

The trial will determine the MTD, safety and the additional benefit achieved from adding SGI-110 to ipilimumab therapy in metastatic melanoma patients. Preclinical evidence generated with SGI-110 in vivo

demonstrated that besides having a direct activity on tumor growth as a single agent, SGI-110 was able to "sensitize" neoplastic cells to the anti-tumor activity of CTLA-4 blockade, providing a sound scientific rationale to develop new immunotherapeutic approaches combining SGI-110 with therapeutic mAb to immune check-points.[14]

1.5 Overall Risk/Benefit Assessment

In the clinical trials conducted to date, SGI-110 has shown antitumor activity with a manageable safety profile. The two main drug-related adverse reactions of SGI-110 are myelosuppression (neutropenia, febrile neutropenia, thrombocytopenia, and anemia) and injection-site events such as pain, irritation, and inflammation. At the SGI-110 doses and schedule proposed in this study (15-60 mg/m^2 day on Days 1-5), there have been no reports of drug-related myelosuppression in MDS and AML subjects in study SGI-110-01. The starting dose of 30 mg/m^2 day on Days 1-5 is 33% of the MTD for single-agent treatment with SGI-110. However, SGI-110 is generally administered in 28 day cycles and in this study will be administered in 21 day cycles. Despite the lack of overlapping toxicity with ipilimumab, the conservative starting dose and opportunity for de-escalation are in place for that reason.

Pain and burning at the injection site has been reported that are related to dose and volume of injection. These AE are Grade 1 and easily managed with icing of the site before and after injection. Other than these events described above, SGI-110 has been well tolerated up to 90 mg/m^2 day x5 and 125 mg/m^2 weekly x3 in 28 day cycles. For more detailed information, please refer to the IB for SGI-110.

The anti-CTLA-4 mAb ipilimumab, improves survival of metastatic melanoma with a well-known safety profile; the most common treatment-related AEs in subjects who received ipilimumab 3 mg/kg were irAEs affecting the skin and GI tract (eg, pruritus, rash, and diarrhea). Established treatment guidelines are actually available to safely manage the immune-related Adverse Events related to ipilimumab. For more detailed information, please refer to the IB for ipilimumab. The safety profile of SGI-110 in combination with ipilimumab will be tested for the first time in this trial, however due to the different nature of two compounds no overlapping toxicity will be expected. Evidences generated with SGI-110 in vivo demonstrated that besides having a direct activity on tumor growth as a single agent, SGI-110 was able to "sensitize" neoplastic cells to the anti-tumor activity of CTLA-4 blockade, improving its efficacy.

2 STUDY OBJECTIVES

2.1 Primary Objective

To assess Maximum Tolerated Dose (MTD) and safety of SGI-110 in combination with ipilimumab in 21 day cycles in melanoma patients.

2.2 Secondary Objectives

- To evaluate the immune-related (ir) -Disease Control Rate (ir-DCR), immune-related (ir) -Objective Response Rate (ir-ORR), immune-related (ir) -Time to Response (ir-TTR) and immune-related (ir) -Duration of Response (ir-DOR)
- To evaluate median immune-related (ir) Progression Free Survival (ir-PFS), median Overall Survival (OS), and survival rate at 1 and 2-years

2.3 Exploratory Objectives

- To investigate immune-biologic correlates to treatment with SGI-110 in combination with ipilimumab (**see Appendix A**)
- Characterize pharmacokinetic profile of SGI-110 and decitabine. For a detailed workplan of pharmacokinetic analyses (**see Appendix B**)

3 ETHICAL CONSIDERATIONS

3.1 Good Clinical Practice

This study will be conducted in accordance with Good Clinical Practice (GCP), as defined by the International Conference on Harmonization (ICH) and in accordance with the ethical principles underlying European Union Directive 2001/20/EC and the United States Code of Federal Regulations, Title 21, Part 50 (21CFR50).

The study will be conducted in compliance with the protocol. The protocol and any amendments and the subject informed consent will receive Institutional Review Board/Independent Ethics Committee (IRB/IEC) approval/favourable opinion prior to initiation of the study.

3.2 Institutional Review Board/Independent Ethics Committee

Before study initiation, the investigator must have written and dated approval/favourable opinion from the IRB/IEC for the protocol, consent form, subject recruitment materials/process (e.g., advertisements), and any other written information to be provided to subjects. The investigator or sponsor should also provide the IRB/IEC with a copy of the Investigator Brochure or product labelling, information to be provided to subjects and any updates.

The investigator or sponsor should provide the IRB/IEC with reports, updates and other information (e.g., expedited safety reports, amendments, and administrative letters) according to regulatory requirements or institution procedures.

3.3 Informed Consent

Investigators must ensure that subjects, or, in those situations where consent cannot be given by subjects, their legally acceptable representatives, are clearly and fully informed about the purpose, potential risks, and other critical issues regarding clinical studies in which they volunteer to participate. Freely given written informed consent must be obtained from every subject or, in those situations where consent cannot be given by subjects, their legally acceptable representative, prior to clinical study participation, including informed consent for any screening procedures conducted to establish subject eligibility for the study. The rights, safety, and well-being of the study subjects are the most important considerations and should prevail over interests of science and society.

Appendix 1 contains Fondazione NIBIT/CRO procedures on obtaining informed consent from subjects, or, in those situations where consent cannot be given by subjects, their legally acceptable representative prior to participating in a clinical study. Procedures are described for all subjects, including those who are unable to give informed consent. The relevant procedures must be used whenever they are applicable (see subject selection criteria in Sections 4.2.1 and 4.2.2)

4 INVESTIGATIONAL PLAN

4.1 Study Design and Duration

This is a phase Ib, dose-escalation study aims to evaluate the safety, tolerability, and Maximum Tolerated Dose (MTD) of SGI-110 in combination with ipilimumab a 21 day cycle in melanoma patients

4.1.1 Study Phases

This study is divided into 3 phases: the Screening Phase, the Treatment Phase, and the Follow-up Phase. After informed consent is obtained, patients will enter the Screening Phase to assess eligibility criteria. Upon meeting criteria, eligible patients will receive either the combination of SGI-110 and ipilimumab.

Eligible subjects will enter a Treatment Phase within 3 days to receive:

SGI-110: starts at 30 mg/m^2 day s.c. on W0, 3, 6, 9 Day 1 - 5 q21 days. Dose Level -1: 15 mg/m^2 day; Dose Level +1: 45 mg/m^2 day, Dose Level +2: 60 mg/m^2 day and ipilimumab: 3 mg/Kg i.v. over 90 minutes on W1, 4, 7 and 10 for a total of 4 cycles.

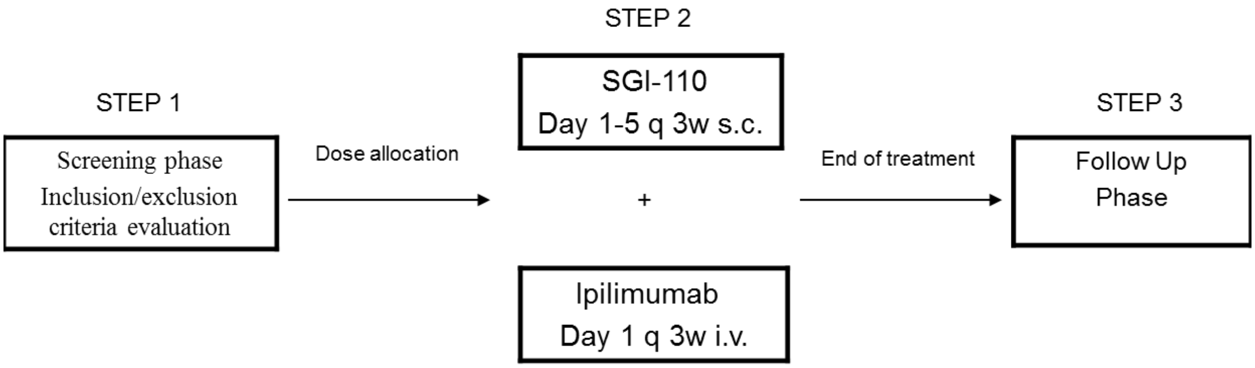

Figure 1

The dose escalation of SGI-110 will follow a traditional 3+3 design: Cohorts of 3-6 patients will receive a combination therapy of SGI-110 and Ipilimumab 3mg/kg. Dose escalation will be dependent on the available toxicity information (including adverse events that are not DLTs), PK, and PD information, as well as the recommendations from the scientific committee. During the dose escalation, subjects will be allocated to 1 of 4 dose levels of SGI-110 in combination with the standard schedule of ipilimumab (see Figure 1). Treatment within the 4 SGI-110 dose levels will proceed in sequence based upon the incidence of DLTs (see Section 5.2.2). Details of this evaluation and the thresholds for toxicity and dose escalation are described fully in Section 5.3. The total number of subjects enrolled will depend on the observed DLTs, and is expected to be up to 19 subjects.

Study Phases are further detailed below:

Screening Phase

- Begins after the patient signs the Informed Consent Form;
- Ends before Treatment Phase;
- Establishes patient eligibility;

- Provides characteristics, including the baseline disease status for the response assessment.
- After informed consent has been obtained, the subject must be enrolled into the study by registration on the eClinical platform to obtain the subject number (STEP1).

Treatment Phase

Following completion of all Screening Phase evaluation, and checking inclusion criteria site personnel will make patient registration to obtain a treatment assignment (STEP2). The e-Clinical system assigns subjects to the SGI-110 dose level.

Follow-up Phase

Begins for the patients when they:

- Withdraw consent from study procedures but not from Follow-up;
- Experience irPD.
- 30 days after final treatment dosing;
- No TAs for patient that experienced irPD;
- Telephone contact every 12 weeks to evaluate overall survival (OS), and inquire about AE resolution, if applicable and subsequent anti-cancer treatments.

Refer to the Flow Chart/Time and Events Schedule in Section 6.0 and Sections 6.3 for a complete and detailed list of all procedures associated with the study phases.

End of Treatment

Subjects should be encouraged to complete as many of the 4 cycles of therapy as possible. The median time to response for both single agents is more than 100 days, thus maximal benefit will only be achieved from the full course. If a subject is removed from treatment for any reason, the subject must undergo end of treatment procedures. The end of treatment procedures include clinical laboratory investigations (chemistry and hematology), urine pregnancy testing, and adverse event assessment.

If a subject is removed from treatment prior to completing 4 cycles due to excessive toxicity the subject remains on TA assessments as per section 4.1.2.

If a subject elects to come off treatment by withdrawing consent before completing 4 cycles, the reason for withdrawal must be entered on the appropriate case report form (CRF) page.

4.1.2 Tumor Assessments (TA)

Tumor assessment with radiographic imaging (e.g. MRI-CT of brain, CT of chest, abdomen, pelvis, other soft tissues) and digital photographs of skin lesions will be performed for all subjects at Screening, week 12, then every 6 weeks at weeks 18, 24, and every 12 weeks from week 24 onwards, for all non-progressing subjects. At each evaluation investigators will assess the overall response (complete response (CR), partial response (PR), stable disease (SD) or Progressive Disease (PD) (as categorized by irRC).

4.1.2.1 Confirmed ir-Disease Progression

Unless they are rapidly deteriorating, those patients who experience ir-disease progression, as determined by the investigator, should have confirmation of irPD at the next scheduled TA (see Tab.6.1). Increases in lesion size detected radiologically or upon gross examination could be misinterpreted as progressive tumor growth since ipilimumab can produce an influx or expansion of tumor infiltrating lymphocytes. Therefore no subsequent anti-cancer treatment should be given prior to confirmed irPD. See Section 6.4.

4.1.2.2 Tumor Assessment Schedule Rationale

Ipilimumab is expected to trigger immune-mediated responses, which require activation of the immune system prior to the observation of clinical responses. Such immune activation may take weeks to months. Some patients with advanced melanoma may have objective volume increase of tumor lesions within 12 weeks after inclusion in the study. Such patients may have not had sufficient time to develop the required immune activation or, in some patients, tumor volume increases may represent infiltration of lymphocytes into the original tumor. In conventional studies, such tumor volume increases during the first 12 weeks of the study would constitute PD and lead to discontinuation of imaging to detect response, thus disregarding the potential for subsequent immune-mediated clinical response. Therefore, in this study, patients with tumor volume increase detected at Week 12 but without rapid clinical deterioration will continue to be clinically observed with a stringent imaging schedule to allow detection of a subsequent tumor response. This will improve the overall assessment of the clinical activity of ipilimumab and more likely capture its true potential to induce clinical responses. Tumor assessments will be made using the immune-related (ir) tumor response criteria. See Sections 6.4.3.5.

4.1.3 Safety Assessment

Safety Monitoring Commitee

A Safety Monitoring Committee (SMC) will be established for the trial.

The SMC will monitor subjects' safety and will review all reported adverse events (AEs) and serious adverse events (SAEs) on an ongoing basis throughout the trial. Information on all SAEs and potential dose-limiting toxicities (DLTs) will be sent to the SMC by the end of the business day on which documented information on SAEs or DLTs was received. Enrollment of subjects into a respective cohort will be paused when the first 3 subjects have entered the first treatment cycle. A full safety data set (all AEs including SAEs, laboratory safety data, vital signs and available pharmacokinetic and pharmacodynamic data) from at least the first 3 weeks (i.e., up to Day 22, until the second cycle of SGI-110 dosing) will be submitted to the SMC.

The SMC will decide, by consensus, on DLTs relevant for the respective combination treatment, and will decide, by consensus, on dose and schedule escalation, dose and schedule de-escalation, dose and schedule expansion, suspension of enrolment, and declaration of the maximum tolerated dose (MTD) for the respective combination treatment.

DLTs are defined in Section 5.3. The process by which the MTD will be defined is described in Section 5.2.2

4.1.4 Study Schema

COHORT	***SGI-110***<br>***Day 1-5 q 3w***	***Ipilimumab***<br>***Day 1 q 3w***
Level -1	***15mg/kg s.c.***	***3 mg/kg i.v.***
Level 0	***30mg/kg s.c.***	***3 mg/kg i.v.***
Level +1	***45mg/kg s.c.***	***3 mg/kg i.v.***
Level +2	***60 mg/kg s.c.***	***3 mg/kg i.v.***

q3w= every 3 weeks;

Figure 2

4.1.5 Rationale for starting dose and for dose escalation schema

At an SGI-110 daily dose range of 60-90 mg/m^2, the observed 5-AZA-CdR AUCs reached or exceeded the therapeutic range seen with IV 5-AZA-CdR (20 mg/m^2) with a lower C_{max}, but a longer effective half-life and exposure window. Dose-related increases in LINE-1 demethylation after SGI-110 dosing was observed in the majority of subjects treated with the Daily x5 regimen between 18 and 60 mg/m^2 day

doses and significant reductions were seen at 36 mg/m^2. Maximum average demethylation (~25%) plateaued after 60 mg/m^2, with daily doses (90-125 mg/m^2) showing no further increase. Therefore the BED for the Daily x5 regimen was established at 60 mg/m^2.

The rationale for selection of the starting dose of SGI-110 for this study is based on previous experience with decitabine in ovarian cancer [29] and emerging data from the Phase 1/2 study of SGI-110 (Study SGI-110-01). The single agent experience in myeloid malignancies was in 28 day cycles. In the Phase 1/2 study of SGI-110, the daily regimen up to 90 mg/m^2 daily x5 was well tolerated with no DLTs. Based on this evidence, the starting dose of the proposed study here is 30 mg/m^2 daily x5, also, due to the 1 week shorter recovery period between dosing investigated in this trial; though no overlapping myelosuppression with ipilimumab is expected. Dose de-escalation to 15 mg/m^2 daily x5 or escalation to 45 -60 mg/m^2 day x5 will be based on the tolerability of the each cohort of 3-6 subjects.

4.1.6 Duration of Study

We assume a planned uniform accrual period of 24 months and a follow-up period of 24 months. Patients will be treated until the prescribed 4 cycles of therapy or until ir Progressive Disease (PD), excessive toxicity, or patient refusal. Upon completion of treatment, all subjects will be followed with efficacy assessments until PD and for survival until 2 years post Last Patient First Visit. The total study duration will be approximately 48 months.

4.2 Study Population

For entry into the study, the following criteria MUST be met within 4 weeks prior to first dose. Any exemptions from the protocol-specific section criteria must be approved by the Principal Investigators and/or Institutional Review Board (IRB) before enrollment.

4.2.1 Inclusion Criteria

Signed Written Informed Consent

Willing and able to give written informed consent.

Target Population

1) Subjects must fulfill all of the following inclusion criteria:
2) Unresectable Stage III or Stage IV melanoma with measurable lesions by CT or MRI per mWHO/irRC criteria, that can be amenable to biopsy

3) Previously treated or untreated; prior therapy may include chemotherapy or targeted therapy for metastatic disease (i.e., BRAF and/or MEK inhibitor). Prior adjuvant interferon is permitted.
4) Subjects with ECOG performance status of 0 to 1
5) 4 weeks or greater since last treatment
6) Must have recovered from any acute toxicity associated with prior therapy
7) Life expectancy greater than 16 weeks
8) Subjects with adequate organ function defined as:
9) WBC ≥ 3500/uL
10) ANC ≥ 2000/uL
11) Platelets ≥ 100 x 10^3/uL
12) Hemoglobin ≥ 9 g/dL
13) Creatinine ≤ 2.5 x ULN
14) AST ≤ 2.5 x ULN for patients without liver metastasis

≤ 5 x ULN for patients with liver metastasis

15) Bilirubin ≤ 1.5 x ULN for patients without liver metastasis

≤ 3 x ULN for patients with liver metastasis

<3.0 mg/mL for patients with Gilbert's Syndrome

16) Negative screening tests for HIV, HepB, and HepC. If positive results are not indicative of true active or chronic infection, the patient can enter the study after discussion and agreement between the Investigator and the Medical Monitor.
17) Women of child-bearing potential must not be pregnant or breastfeeding, must have a negative pregnancy test at Screening and all men must be practicing two medically acceptable methods of birth control. Men should not father a child while receiving treatment with SGI-110 + ipilimumab, and for 2 months following completion of treatment. Men with female partners of childbearing potential should use effective contraception during this time.

Age and Sex

Men and women of and over 18 years old.

Women of childbearing potential (WOCBP) must use appropriate method(s) of contraception. WOCBP should use an adequate method to avoid pregnancy for 23 weeks (30 days plus the time required for ipilimumab to undergo five half-lives) after the last dose of investigational drug

WOCBP include any female who has experienced menarche and who has not undergone successful surgical sterilization (hysterectomy, bilateral tubal ligation, or bilateral oophorectomy) or is not postmenopausal. Post menopause is defined as:

1) Amenorrhea ≥ 12 consecutive months without another cause or
2) For women with irregular menstrual periods and on hormone replacement therapy (HRT), a documented serum follicle stimulating hormone (FSH) level > 35 mIU/mL.

Women who are using oral contraceptives, other hormonal contraceptives (vaginal products, skin patches, or implanted or injectable products), or mechanical products such as an intrauterine device or barrier methods (diaphragm, condoms, spermicides) to prevent pregnancy, or are practicing abstinence or where their partner is sterile (eg, vasectomy) should be considered to be of childbearing potential.

WOCBP must have a negative serum or urine pregnancy test (minimum sensitivity 25 IU/L or equivalent units of HCG) within 24 hours prior to the start of investigational product.

Men who are sexually active with WOCBP must use any contraceptive method with a failure rate of less than 1% per year Men receiving ipilimumab and who are sexually active with WOCBP will be instructed to adhere to contraception for a period of 23 weeks after the last dose of investigational product Women who are not of childbearing potential (i.e., who are postmenopausal or surgically sterile as well as azoospermic men do not require contraception).

4.2.2 Exclusion Criteria

Subjects meeting any of the following exclusion criteria will be excluded from the study:

1) Subjects with any contraindications for ipilimumab
2) Subjects with active brain metastases or leptomeningeal metastases
3) Subjects with metastatic uveal melanoma
4) Subjects with active, known or suspected autoimmune disease
5) Subjects with a condition requiring systemic treatment with either corticosteroids (>10 mg daily prednisone equivalents) or other immunosuppressive medications within 14 days of treatment
6) Subjects with symptomatic effusions on account of pleural, pericardial metastases of melanoma

7) Prior treatment with an anti-Programmed Death receptor-1 (PD-1), anti-Programmed Death-1 ligand-1 (PD-L1), anti-PD-L2, or anti-CTLA-4 antibody
8) Subjects who had major surgery or radiation therapy within 21 days of starting treatment
9) Subjects who are unable to return for follow-up visits as required by this study.
10) Subjects with a history of second malignant tumor, other than the common skin cancers – basal and squamous carcinomas, within the past 3 years and uncertainty about the histological nature of the metastatic lesions.

Other Exclusion Criteria

a) Prisoners or subjects who are involuntarily incarcerated;
b) Subjects who are compulsorily detained for treatment of either a psychiatric or physical (e.g., infectious disease) illness.

Eligibility criteria for this study have been carefully considered to ensure the safety of the study subjects and to ensure that the results of the study can be used. It is imperative that subjects fully meet all eligibility criteria.

4.2.3 Discontinuation of Subjects from Treatment

Subjects will be treated with combination therapy until the completion of 4 cycles of therapy, irPD, subject refusal or excessive toxicity.

Subjects MUST discontinue investigational product (and non investigational product at the discretion of the investigator) (investigational or non investigational treatment) for any of the following reasons:

- Withdrawal of informed consent (subject’s decision to withdraw for any reason),
- DLT,
- Any clinical adverse event (AE), laboratory abnormality or intercurrent illness that, in the opinion of the investigator, indicates that continued participation in the study is not in the best interest of the subject,

NOTE: In case of excessive toxicity, if the observed AE leading to treatment discontinuation is unequivocally attributable to only 1 of the 2 investigational drugs, patient might still receive the other drug at the investigator discretion

- Pregnancy (see Section 7.7),

- Termination of the study by NIBIT,
- Loss of ability to freely provide consent through imprisonment or involuntarily incarceration for treatment of either a psychiatric or physical (eg, infectious disease) illness.

All subjects who discontinue should comply with protocol specified follow-up procedures as outlined in Section 6. The only exception to this requirement is when a subject withdraws consent for ALL study procedures or loses the ability to consent freely (i.e, is imprisoned or involuntarily incarcerated for the treatment of either a psychiatric or physical illness).

If a subject was withdrawn before completing the study, the reason for withdrawal must be entered on the appropriate case report form (CRF) page.

5 TREATMENTS

5.1 Study Treatment

All protocol-specified investigational and non-investigational products are considered study drug.

5.1.1 Investigational Product

An investigational product, also known as investigational medicinal product in some regions, is defined as follows:

A pharmaceutical form of an active substance or placebo being tested or used as a reference in a clinical study, including products already with a marketing authorization but used or assembled (formulated or packaged) in a way different from the authorized form, or used for an unauthorized indication, or when used to gain further information about the authorized form.

In this protocol, investigational products are SGI-110 and ipilimumab. Astex will supply SGI-110 to Fondazione NIBIT/CRO for this trial. Ipilimumab will be utilized in the study according to the approved dose and schedule of administration for metastatic melanoma.

5.1.2 Identification

Table 5.1.3: Investigational Drug Information

Unit	*Route*	*Appearance*
**Ipilimumab 5 mg/mL**	**IV infusion**	**Clear, colorless solution in 50 mg (10 mL) or 200 mg (40 mL) vial**
**SGI-110 100 mg**	**SC**	**Dry powder lyophilized**
**SGI-110 diluent 3 ml**		**Non- aqueous diluent**

5.1.3 Packaging and Labeling

Study drug will be provided as follows:

Ipilimumab and SGI-110 labels will contain the protocol prefix, batch number, contents, route of administration, storage conditions and appropriate IND caution statement.

5.1.4 Handling and Dispensing

Study drug supplied to the sponsor or sourced by the investigator should be stored in a secure area according to local regulations. It is the responsibility of the investigator to ensure that study drug is only dispensed to study subjects. The study drug must be dispensed only from official study sites by authorized personnel according to local regulations.

The investigator should ensure that the study drug is stored in accordance with the environmental conditions (temperature, light, and humidity) as determined by the sponsor and defined in the Investigator Brochure for SGI-110 and ipilimumab. If concerns regarding the quality or appearance of the study drug arise, do not dispense the study drug and contact the sponsor immediately.

Please refer to Section 9.2.2 for information on study drug record retention and 9.3 for destruction instructions

SGI-110

SGI-110 should be reconstituted under aseptic conditions and kept under refrigerated conditions.

Drug product is available as a two-vial system, referred to as (1) SGI-110 for Injection, 100 mg and (2) SGI-110 Diluent for Reconstitution, 3 mL:

- SGI-110 for Injection, 100 mg contains SGI-110, 100 mg of free acid equivalent, as a dry powder lyophile.
- SGI-110 Diluent for Reconstitution, 3 mL contains 3 mL of a non-aqueous diluent for reconstitution.

SGI-110 for Injection, 100 mg vial is stored at 2°C to 8°C in the original packaging until use. SGI-110 Diluent for Reconstitution, 3 mL can be stored at 2°C to 30°C in an upright position until use. Both vials are preservative free and for single use only.

The diluent is comprised of 3 commonly used excipients, propylene glycol, glycerin, and ethanol, that are generally recognized as safe (GRAS). All 3 excipients are pharmaceutically acceptable solvents previously used in drug products approved for SC administration. SGI-110 solution is reconstituted at a maximum concentration of 100 mg/mL for SC administration. Both product and diluent are contained in clear glass vials stoppered with a latex-free rubber stopper and capped with an aluminum flip-off seal. The reconstituted SGI-110 solution is chemically stable for 8 days under refrigerated conditions; however, there are no preservatives in the custom diluent or lyophilized vials. For study centers using the PhaSeal® system for reconstitution of SGI-110, it is recommended to use the Assembly Fixture provided by the manufacturer to attach the PhaSeal® Protector to the SGI-110 lyophilized vial and custom diluent vial, and to filter the final reconstituted product with a 0.2 micron filter. This is to eliminate the presence of foreign material resulting from stopper coring in the final dispensed product.

Ipilimumab

Ipilimumab (BMS-734016) Injection (5 mg/ml), must be stored refrigerated (2 - 8°C) with protection from light. Ipilimumab injection must not be frozen.

Ipilimumab injection may be stored undiluted (5 mg/mL) or following dilution on 0.9% Sodium Chloride Injection (USP), or 5% Dextrose Injection in PVC, non-PVC/ non-DEHP, or glass containers for up to 24 hours at (2°C - 8°C) or room temperature/room light. This would include any time in transit and the total time for infusion.

5.2 Method of Assigning Subjects to a Treatment

After informed consent has been obtained, the patient will be enrolled into the study by a dedicated software, that will be available to the participant Center, and obtain a patient number. The exact procedures for using the dedicated software will be detailed in a separate document.

Following completion of all Screening Phase evaluation, and checking inclusion criteria site personnel will register the patient to obtain a treatment assignment (STEP2). The e-Clinical system assigns subjects to the SGI-110 dose level.

5.2.1 Dosage and Administration

SGI-110 schedule

SGI-110 will be administered:

Start at 30 mg/m^2 day s.c. on W0, 3, 6, 9 Day 1 - 5 q21 days. Dose Level -1: 15 mg/m^2 day; dose level +1: 45 mg/m^2 day, Dose Level +2: 60 mg/m^2 day for a total of 4 cycles.

SGI-110 is administered by SC injection preferably in the abdominal area; however the thigh and arms may be used. A Luer-Lok disposable syringe should be used when dispensing and administering SGI-110. Due to the slightly viscous nature of the preparation, use of thin-walled subcutaneous needle is highly recommended for ease of subcutaneous administration and subject comfort. SGI-110 should be injected slowly (up to one minute) as some injection site discomfort or pain may be experienced.

If injection site pain is reported upon injection, apply ice packs to the injection site both before and after injection. If the injection site events are reported at subsequent injections despite slow injection and the use of ice packs, pre-treatment with topical or systemic analgesics can be considered. The total amount (in mg) of SGI-110 to be administered will be determined based on the body surface area (BSA). In calculating the BSA, actual heights and weights taken at enrolment should be used. There will be no adjustments to “ideal” body weight or dose modifications related to changes in body weight < +/- 10%. The institutional standard for calculating BSA is acceptable.

The site(s) of SGI-110 SC injections will be captured on the dosing CRF.

Ipilimumab schedule

Ipilimumab will be administered:

3 mg/Kg i.v. over 90 minutes on W1, 4, 7 and 10 for a total of 4 cycles.

Ipilimumab is to be administered as a 90-minute IV infusion, using a volumetric pump with a 0.2/0.22 micron in-line filter at the protocol-specified dose. The drug can be diluted with 0.9% normal saline for delivery but the total drug concentration of the solution cannot be below 0.35 mg/ml. It is not to be administered as an IV push or bolus injection. At the end of the infusion, flush the line with a sufficient quantity of normal saline.

5.2.2 Dose Escalation

Dose escalation will be dependent on the available toxicity information (including adverse events that are not DLTs), PK, and efficacy information, as well as the recommendations from the scientific committee.

The dose escalation of SGI-110 will follow a traditional 3+3 design Cohorts of 3-6 patients will receive a combination therapy of SGI-110 and Ipilimumab 3mg/kg. Each patient will receive SGI-110 s.c. for 5 consecutive days (D1-5) during a 3-week cycle at one of the following doses:

- Dose Level -1: 15 mg/m^2 day
- Dose Level 0: 30 mg/m^2 day
- Dose Level +1: 45 mg/m^2 day
- Dose Level +2: 60 mg/m^2 day

In the absence of dose-limiting toxicities (DLTs), the starting dose of SGI-110 of 30 mg/m^2 day will be escalated to 45 mg/m^2 day on Days 1-5. In the absence of DLT in Dose Level +1, the dose of SG1-110 of 45 mg/m^2 day will be escalated to 60 mg/m^2 day on Days 1-5 (Level +2). In the presence of 1 DLT in 3 subjects of the single dose cohort, the cohort will enroll 3 more subjects at the same dose level. In the event that 2 or more DLTs were encountered at the single dose cohort, the previous lower dose level will be evaluated. The MTD will be the highest SGI-110 Dose Level (15-60 mg/m^2 day on Days 1-5) in combination with ipilimumab at which no more than 1 of 6 subjects experiences a DLT. Once the MTD is established, additional subjects will be treated at that dose up to a total of 19 subjects in the entire study.

5.3 Dose-limiting Toxicity

DLT is defined as any of the following events occuring during the first treatment cycle and clearly related to study treatment: grade 4 neutropenia (absolute granulocyte count < 0.5 x 10^9/L, ≥ 5 days), febrile neutropenia grade ≥ 3 (absolute granulocyte count < 1.0 x 10^9/L and fever ≥ 38.5° C), platelet count < 25.000/L or thrombocytopenic bleeding, AST or ALT grade ≥ 3 for 7 days, any grade 3 or 4 non-hematologic toxicity (excluding alopecia, non pre-medicated nausea and vomiting), grade 3 or 4 clinically

significant nausea, vomiting, or diarrhoea in the presence of maximal supportive care, a required interruption of treatment > 2 weeks due to toxicity. If the toxicity fails to resolve to ≤ grade 2 with 14 days off treatment, the patient will be removed from the study.

Inability to initiate Cycle 2 within 2 weeks of scheduled treatment due to slow recovery from study drug-induced toxicities and failure to meet re-treatment criteria.

The DLT observation period is defined as the first 3 weeks of treatment, i.e., from Day 1 to Day 21 of Cycle 1. Once the MTD is reached in each group, the safety data will be analysed.

Note: All AEs (including those occurring after the end of the DLT observation period) will be considered for decision-making by the SMC.

Tolerability will be defined as the ability to continue treatment for at least 2 cycles in a row without development of DLTs.

5.3.1 Dose Modifications

During dose escalation, the dose of SGI-110 to be administered is determined by the dose level and the subject's body weight at consent. Thereafter, it will be determined by the MTD and the subject's body weight. Once a dose of SGI-110 is established, it should be maintained unless the subject's body weight decreases by 10% or a safety issue requires dose reduction. If a myelosuppressive DLT occurs, the patient may resume at the next lower dose level of SGI-110 after recovery of counts.

Dose Delay Criteria for SGI-110

The Investigator should try to the best of his/her ability to assess whether an adverse event is related to SGI-110 treatment. If the adverse event is judged to be related to SGI-110 dosing delay may be recommended according to the following guidelines:

In case of drug-related Grade 3 or 4 non-hematological toxicity, or Grade 4 hematological toxicity, dosing should be delayed until the subject recovers to levels consistent with the eligibility criteria if specified, or the subject's baseline status otherwise, and dosing should be resumed at the same dose. In case of asymptomatic uncomplicated Grade 4 hematological toxicity that lasts no more than one week, a subject may be redosed at the same dose level following recovery during the week prior to the next dose of ipilimumab as specified above per investigator's judgment.

Dose delay criteria apply for all drug-related adverse events (regardless of whether or not the event is attributed to SGI-110). All study drugs must be delayed until treatment can resume.

Dose Delay Criteria for ipilimumab

Because of the potential for clinically meaningful ipilimumab-related AEs requiring early recognition and prompt intervention, management algorithms have been developed for suspected AEs of selected categories.

Subjects may resume treatment with study drug when the drug-related AE(s) resolve to Grade ≤ 1 or baseline value, with the following exceptions:

- Subjects may resume treatment in the presence of Grade 2 fatigue,
- Subjects who have not experienced a Grade 3 drug-related skin AE may resume treatment in the presence of Grade 2 skin adverse event,
- Subjects with baseline Grade 1 AST/ALT or total bilirubin who require dose delays for reasons other than a 2-grade shift in AST/ALT or total bilirubin may resume treatment in the presence of Grade 2 AST/ALT OR total bilirubin,
- Subjects with combined Grade 2 AST/ALT AND total bilirubin values meeting discontinuation parameters should have treatment permanently discontinued,
- Drug-related pulmonary AEs, diarrhea, or colitis, must have resolved to baseline before treatment is resumed,
- Drug-related endocrinopathies adequately controlled with only physiologic hormone replacement may resume treatment.

Dose delay criteria apply for all drug-related adverse events (regardless of whether or not the event is attributed to ipilimumab). All study drugs must be delayed until treatment can resume.

If the criteria to resume treatment are met, the subject should restart treatment at the next scheduled timepoint per protocol. However, if the treatment is delayed past the next scheduled timepoint per protocol, the next scheduled timepoint will be delayed until dosing resumes. If treatment is delayed > 6 weeks, the subject must be permanently discontinued from study therapy, except as specified below.

5.3.2 Discontinuation Criteria

Ipilimumab Permanent Discontinuation

Treatment should be permanently discontinued for the following:

- Any Grade 2 drug-related uveitis or eye pain or blurred vision that does not respond to topical therapy and does not improve to Grade 1 severity within the re-treatment period OR requires systemic treatment
- Any Grade 3 non-skin, drug-related adverse event lasting > 7 days, including uveitis, pneumonitis, bronchospasm, diarrhea, colitis, neurologic adverse event, hypersensitivity reactions, and infusion reactions. Drug-related laboratory abnormalities are exceptions, as discussed below.

o Grade 3 drug-related uveitis, pneumonitis, bronchospasm, diarrhea, colitis, neurologic adverse event, hypersensitivity reaction, or infusion reaction of any duration requires discontinuation

o Grade 3 drug-related laboratory abnormalities do not require treatment discontinuation except those noted below

a. Grade 3 drug-related thrombocytopenia > 7 days or associated with bleeding requires discontinuation
b. Any drug-related liver function test (LFT) abnormality that meets the following criteria require discontinuation:
   - AST or ALT > 8 x ULN
   - Total bilirubin > 5 x ULN
   - Concurrent AST or ALT > 3 x ULN and total bilirubin > 2 x ULN

- Any Grade 4 drug-related adverse event or laboratory abnormality, except for the following events which do not require discontinuation:

o Isolated Grade 4 amylase or lipase abnormalities that are not associated with symptoms or clinical manifestations of pancreatitis and decrease to < Grade 4 within 1 week of onset.

o Isolated Grade 4 electrolyte imbalances/abnormalities that are not associated with clinical sequelae and are corrected with supplementation/appropriate management within 72 hours of their onset

- Any dosing interruption lasting > 6 weeks with the following exceptions:

o Dosing interruptions to allow for prolonged steroid tapers to manage drug-related adverse events are allowed. Prior to re-initiating treatment in a subject with a dosing interruption lasting > 6 weeks, the Investigator must be consulted. Tumor assessments should continue as per protocol even if dosing is interrupted

- o Dosing interruptions > 6 weeks that occur for non-drug-related reasons may be allowed if approved by the Investigator. Prior to re-initiating treatment in a subject with a dosing interruption lasting > 6 weeks, the Investigator must be consulted. Tumor assessments should continue as per protocol even if dosing is interrupted

- Any adverse event, laboratory abnormality, or intercurrent illness which, in the judgment of the Investigator, presents a substantial clinical risk to the subject with continued ipilimumab dosing

Liver Function Tests (LFT) Assessments Required Prior to Administration of ipilimumab

Liver function tests (AST, ALT, T. bilirubin) will be evaluated for every subject prior to administration of ipilimumab. Blood samples must be collected and analyzed at local or central labs within 3 days prior to dosing. LFT results must be reviewed by the principal investigator (or designee) to meet dosing criteria specifications: ≤ 3 x ULN for AST, ALT and ≤ 1.5 x ULN for T. bilirubin unless liver metastases are present in which case LFT ≤ 5 x ULN for AST, ALT and T. bilirubin ≤ 3.0 x ULN) prior to dosing.

Although infrequent, liver toxicity may be associated to the administration of SGI-110. It could possibly occur around Week 4 or 5. Special attention should thus be paid to LFT results prior to the ipilimumab dosing on Weeks 4 and 7.

If, during the course of treatment abnormal LFT values are detected, the subject must be managed using the hepatotoxicity algorithm section of the ipilimumab Investigators Brochure.

Treatment of ipilimumab Related Infusion Reactions

Since ipilimumab contains only human immunoglobulin protein sequences, it is unlikely to be immunogenic and induce infusion or hypersensitivity reactions. However, if such a reaction were to occur, it might manifest with fever, chills, rigors, headache, rash, pruritis, arthralgias, hypo- or hypertension, bronchospasm, or other symptoms.

All Grade 3 or 4 infusion reactions should be reported as an SAE if criteria are met. Infusion reactions should be graded according to NCI CTCAE 4.0 guidelines.

Treatment recommendations are provided below and may be modified based on local treatment standards and guidelines as appropriate:

For Grade 1 symptoms: (Mild reaction; infusion interruption not indicated; intervention not indicated)

Remain at bedside and monitor subject until recovery from symptoms. The following prophylactic premedications are recommended for future infusions: diphenhydramine 50 mg (or equivalent) and/or

paracetamol 325 to 1000 mg (acetaminophen) at least 30 minutes before additional ipilimumab administrations.

For Grade 2 symptoms: (Moderate reaction requires therapy or infusion interruption but responds promptly to symptomatic treatment [eg, antihistamines, non-steroidal anti-inflammatory drugs, narcotics, corticosteroids, bronchodilators, IV fluids]; prophylactic medications indicated for ~ 24 hours).

Stop the ipilimumab infusion, begin an IV infusion of normal saline, and treat the subject with diphenhydramine 50 mg IV (or equivalent) and/or paracetamol 325 to 1000 mg (acetaminophen); remain at bedside and monitor subject until resolution of symptoms. Corticosteroid or bronchodilator therapy may also be administered as appropriate. If the infusion is interrupted, then restart the infusion at 50% of the original infusion rate when symptoms resolve; if no further complications ensue after 30 minutes, the rate may be increased to 100% of the original infusion rate. Monitor subject closely. If symptoms recur then no further ipilimumab will be administered at that visit. Administer diphenhydramine 50 mg IV, and remain at bedside and monitor the subject until resolution of symptoms. The amount of study drug infused must be recorded on the electronic case report form (eCRF). The following prophylactic premedications are recommended for future infusions: diphenhydramine 50 mg (or equivalent) and/or paracetamol 325 to 1000 mg (acetaminophen) should be administered at least 30 minutes before additional ipilimumab administrations. If necessary, corticosteroids (recommended dose: up to 25 mg of IV hydrocortisone or equivalent) may be used.

For Grade 3 or Grade 4 symptoms: (Severe reaction, Grade 3: prolonged [i.e., not rapidly responsive to symptomatic medication and/or brief interruption of infusion]; recurrence of symptoms following initial improvement; hospitalization indicated for other clinical sequelae [e.g., renal impairment, pulmonary infiltrates]). Grade 4: (life threatening; pressor or ventilatory support indicated).

Immediately discontinue infusion of ipilimumab. Begin an IV infusion of normal saline, and treat the subject as follows. Recommend bronchodilators, epinephrine 0.2 to 1 mg of a 1:1,000 solution for subcutaneous administration or 0.1 to 0.25 mg of a 1:10,000 solution injected slowly for IV administration, and/or diphenhydramine 50 mg IV with methylprednisolone 100 mg IV (or equivalent), as needed. Subject should be monitored until the investigator is comfortable that the symptoms will not recur. Ipilimumab will be permanently discontinued. Investigators should follow their institutional guidelines for the treatment of anaphylaxis. Remain at bedside and monitor subject until recovery from symptoms. In the case of late-occurring hypersensitivity symptoms (e.g., appearance of a localized or generalized pruritis within 1 week after treatment), symptomatic treatment may be given (e.g., oral antihistamine, or corticosteroids).

Re-treatment with Ipilimumab Following Infusion Reactions

Once the ipilimumab infusion rate has been decreased due to an infusion reaction, it will remain decreased for all subsequent infusions. If the subject has a second allergic/infusion reaction with the slower infusion rate, the infusion should be stopped and the subject should be discontinued from treatment. If a subject experiences a Grade 3 or 4 allergic infusion reaction at any time, the subject should be discontinued from treatment. If there is any question as to whether an observed reaction is an allergic/infusion reaction of Grades 1 - 4, the principal investigator should be contacted immediately to discuss and grade the reaction.

Management of irAEs

Immune-mediated toxicities have been described as drug-related side effects following ipilimumab treatment including increase in liver function tests, endocrinopathies and diarrhea. Such events could be severe and life-threatening. Therefore, surveillance for induction of such events, close monitoring of subjects and prompt initiation of medical management is highly recommended.

Please refer to the most current version of the ipilimumab Investigator Brochure for management of immune related adverse events.

5.4 Concomitant Treatments

In general, concomitant medications and therapies deemed necessary for the supportive care and safety of the subject are allowed, provided their use is documented in the subject records and on the appropriate case report form. If toxicity occurs, the appropriate treatment will be used to ameliorate signs and symptoms (including growth factors for severe hematological toxicity, antiemetics for nausea and vomiting, anti-diarrheals for diarrhea, anti-pyretics and anti- histamines for drug fever, and broad spectrum antibiotics for febrile neutropenia or infection). All supportive measures for optimal medical care will be given during the period of study.

5.4.1 Antibiotics

Antibiotics may be utilized to prevent or manage febrile neutropenia based on institutional standard practice. Febrile neutropenia is defined as temperature at least 38.5C° when the ANC is < 1000 µL. Febrile subjects should be evaluated by physical examination, complete blood count (CBC) with differential, and blood culture. Subjects with febrile neutropenia or suspected sepsis on the basis of the physical examination are to be hospitalized for appropriate broad spectrum antibiotic coverage, consistent with local pathogen sensitivities.

5.4.2 Hematopoietic Growth Factors

Granulocyte-colony stimulating factor (GCSF) and other white blood cell stimulating factors may be administered during Cycle 1 and onwards according to ASCO/ESMO guidelines[30], accepted practice or institutional guidelines, at the discretion of the treating physician. RBC transfusions can be administered at the discretion of the treating physician.

5.4.3 Prohibited and/or Restricted Treatments

Patients may not use any of the following therapies during the course of the study:

- IL-2, interferon or other non-study anti-melanoma immunotherapy regimens;
- Cytotoxic chemotherapy;
- Immunosuppressive agents;
- CD137 agonist;
- CTLA-4 agonists or antagonists;
- Investigational therapies;
- Vaccines (including those for common medical conditions, for up to 1 month pre and post dosing with ipilimumab);
- Chronic systemic steroids;
- Any anti-cancer therapy prior to PD;

Once a patient has entered into the Follow-up Phase, there will be no prohibited therapies.

5.4.4 Other Restrictions and Precautions

Caution is advised when considering treatment with high-dose IL-2 in patients who have previously been administered ipilimumab, particularly in patients who experienced ipilimumab-related diarrhea/colitis. Colonoscopy or sigmoidoscopy with biopsy may be advisable prior to IL-2 administration once the patient is no longer receiving ipilimumab (i.e., is in Follow-up or is no longer participating in the study).

5.5 Treatment Compliance

Trained medical personnel will administer the investigational treatments to patients. Treatment compliance will be monitored by drug accountability, as well as by recording drugs administration in the patient's medical record and CRF. Drug accountability forms in the pharmacy will also be reviewed.

6 STUDY ASSESSMENTS AND PROCEDURES

6.1 Flow Chart/Time and Events Schedule

Procedure	*Screening Phase*		*Treatment Phase (± 3 days)*								*Follow-up Phase*				*End of Treatment*	*Protocol Section*
Timepoint	*Day -28 to Day -1*	*Wk 0*	*Wk 1*	*Wk 2*	*Wk 3*	*Wk4*	*Wk 6*	*Wk7*	*Wk9*	*Wk10*	*Wk12*	*Wk 18*	*Wk 24*	*Wks36+*		
Visit	*0*	*1*	*2*	*3*	*4*	*5*	*5*	*7*	*8*	*9*	*10*	*11*	*12*	*13*		
Eligibility Assessments																
Informed Consent	X															
Inclusion/Exclusion Criteria	X															
Demographics/Medical History	X															
Hepatitis B, C, and HIV test	X															6.3.7.4
Pregnancy test	X	X	X	X	X	X	X	X	X	X	X	X	X	X	X	
Physical Examination	X	X[a]														
Safety Assessments		X[b]	X	X	X	X	X	X	X	X	X	X	X	X	X	
Adverse Event Assessment	X	X	X	X	X	X	X	X	X	X	X	X	X	X	X	
ECOG Performance Status (prior to dosing)	X	X	X	X	X	X	X	X	X	X	X	X	X	X	X	
Concomitant Medications	X	X	X	X	X	X	X	X	X	X	X	X	X	X	X	
Weight/BSA (prior to dosing)	X	X	X	X	X	X	X	X	X	X	X	X	X	X		

Procedure	***Screenig Phase***		***Treatment Phase (± 3 days)***								***Follow-up Phase***				***End of Treatment***	***Protocol Section***
Timepoint	***Day -28 to Day-1***	***Wk 0***	***Wk 1***	***Wk 2***	***Wk 3***	***Wk4***	***Wk 6***	***Wk7***	***Wk 9***	***Wk10***	***Wk12***	***Wk 18***	***Wk 24***	***Wks36+***		
Visit	***0***	***1***	***2***	***3***	***4***	***5***	***6***	***7***	***8***	***9***	***10***	***11***	***12***	***13***		
Laboratory Tests																
Chemistry & Hematology (prior to dosing)	***X***	***X***	***X***	***X***	***X***	***X***	***X***	***X***	***X***	***X***	***X***	***X***	***X***	***X***	***X***	6.3.7.16 .3.7.2
Urine analysisb	***X***															
Paraffin-embedded tumor tissue for B-Raf status assessment (if available)	***X***															
Translational Research	***X***	***X***	***X***		***X***	***X***					***X***		***X***			6.4.3
Pharmacokinetic		***X***														6.4.3
Imaging (brain, neck, chest, abdomen, pelvis ± soft tissue) and Skin Lesion Assessmentc,e	***X***										***X***	***X***	***X***	***X***		6.4.3.3
Brain MRI	***X***															
Tumor biopsy	***X***					***X***					***X***					6.3.8

Procedure	***Screenig Phase***		***Treatment Phase (± 3 days)***								***Follow-up Phase***				***End of Treatment***	***Protocol Section***
Timepoint	***Day -28 to Day-1***	***Wk0***	***Wk1***	***Wk2***	***Wk3***	***Wk4***	***Wk6***	***Wk7***	***Wk9***	***Wk10***	***Wk12***	***Wk18***	***Wk24***	***Wks36+***		
Visit	***0***	***1***	***2***	***3***	***4***	***5***	***6***	***7***	***8***	***9***	***10***	***11***	***12***	***13***		
Treatment Administration																
SGI-110 Administration		***X***			***X***		***X***		***X***							
Ipilimumab Infusion			***X***			***X***		***X***		***X***						

A. *If Screening physical is conducted within 24 hours of study drug dosing then a single physical may count as both the Screening and Week 1 examination. Additional Physical Examinations should be performed as clinically indicated. Vital signs to be performed as described in Section 6.3.2.*

B. *The visits will occur on every treatment day and also on Day 8, for all cycles, on Day 8,15 for the first cycle. There is a 30-day (+ 5 day) safety visit after the last study treatment in this study.*

C. *Urine analysis to be performed at Screening and then as clinically indicated during the study.*

D. *CT Scans with IV contrast and oral contrast of neck, chest, abdomen, pelvis, and ± soft tissue (or MRI, if iodine contrast media is medically contra-indicated) have to be acquired for ALL patients at all TA timepoints. TA will be performed for all subjects at Screening and 12, then every 8 weeks at Week 20, 28, 36, then every 12 weeks from Week 36 onwards for all non-progressing patients.*

E. *Patients who discontinue dosing when being progression free will be followed with Tumor Assessments until PD, unless patient withdraws consent.*

6.2 Study Materials

Fondazione NIBIT will provide Case Report Forms.

6.3 Safety Assessments

Data for the procedures and assessments specified in this protocol should be submitted to Fondazione NIBIT/CRO on a case report form. Additional procedures and assessments may be performed as part of standard of care; however, data for these assessments should remain in the subject's medical record and should not be provided to Fondazione NIBIT/CRO, unless specifically requested.

All subjects who receive at least 1 dose of study drug will be evaluable for safety parameters. Additionally, any occurrence of non-SAE or SAE from time of consent forward, up to and including follow-up visits, will be reported. See Section 7: Adverse Event Reporting.

The visits will occur on every treatment day and also on Day 8, for all cycles, on Day 8,15 for the first cycle. There is a 30-day (+ 5 day) safety visit after the last study treatment in this study.

Safety will be evaluated for all treated subjects using the NCI CTCAE version 4.0. Safety assessments will be based on medical review of AE reports and the results of vital sign measurements, physical examinations and clinical laboratory tests. The incidence of AEs will be tabulated and reviewed for potential significance and clinical importance.

Safety monitoring will be performed on an on-going basis as described in Section 4.1.3.

6.3.1 Medical History, Physical Exam, Physical Measurements

A detailed Medical History will be obtained at Screening. Medical history must include date of diagnosis, including histological or cytological documentation of malignancy. Any toxicity related to previous treatments should be mentioned, if applicable.

A complete physical examination including subject's height and weight will be performed at Screening. Prior to dosing (within 3 days), the subject's weight must be obtained to calculate the ipilimumab dose and the subject's body surface area on which SGI-110 dose is calculated.

If the Screening physical exam is conducted within 24 hours of dosing on Week 0, then a single examination may count as both Screening and Pre-dose examination. Subsequent physical exams will be performed if clinically indicated. Any physical examination finding that qualifies as an AE or SAE must be documented on the appropriate CRF pages. Pre-treatment events present within 2 weeks of starting therapy, whether or not related to current disease, will be captured prior to the study start. Any worsening (≥1 Grade from Baseline) will be documented as an AE in the CRF.

6.3.2 Vital Signs

Vital signs consist of blood pressure, heart rate, and temperature.

During ipilimumab infusions, vital sign measurements except body temperature must be performed prior to dosing, every 30 minutes for the duration of the infusion and 1 hour following completion of the infusion or until the patient is stable. Orthostatic (supine and standing) BP and heart rate are to be measured when clinically indicated (e.g., experiencing light-headedness, dizziness, syncope).

On-treatment clinically significant findings will be reported on the AE CRF.

6.3.3 Pregnancy Testing

WOCBP are required to have several pregnancy tests performed. A negative serum pregnancy test must be documented at the Screening. Additionally WOCBP must exhibit a negative serum or urine pregnancy test (minimum sensitivity 25 IU/L or equivalent units of HCG) within 24 hours prior to the start of study drug, therefore the Screening pregnancy test may need to be repeated prior to the start of study drug dosing.

A pre-dosing urine pregnancy test must be performed prior to each ipilimumab dosing as outlined in Table 6.1.

6.3.4 ECOG Status

ECOG performance status will be evaluated at the screening evaluation and at each visit as outlined in Table 6.1. If patient discontinues SGI-110 dosing but remains on ipilimumab, performance status will be reported prior to ipilimumab dosing only.

6.3.5 Adverse Events Monitoring

Adverse Events (AEs) will be evaluated according to the NCI CTCAE Version 4.0 on a continuous basis starting from when the subject takes the first dose of study treatment, up to and including Follow-up visits (at minimum, for 70 days following last dosing).

Serious Adverse Events (SAEs) must be collected from the time period following written consent to participate in the study up to and including Follow-up visits (at minimum, for 70 days following last dosing).

6.3.6 Concomitant Medications

Concomitant medications will be recorded in the concomitant medication log. Any changes to concomitant medications will be recorded at each visit.

Any systemic or local corticosteroid that is prescribed for treatment emergent or disease related medical conditions will be recorded on the concomitant medication log. The information on corticosteroids must include the name of the drug, dose, dosing frequency and any information relevant to the use of

corticosteroids used to treat irAEs or emergent neurological complications. Investigators should add any clinical notes about the response to corticosteroid therapy as it relates to clinical or radiologic status changes.

6.3.7 Laboratory Test Assessments

6.3.7.1 Serum Chemistry

Serum Chemistry is to be obtained as outlined in Table 6.1. Serum chemistry tests are to include: albumin, amylase, lipase, BUN, creatinine, ALT, AST, LDH, serum alkaline phosphatase, direct and total bilirubin, glucose, total protein, sodium, potassium, chloride, HCO3, calcium, uric acid and TSH. Additional draws must be incorporated when monitoring recovery from any non-hematologic AE (e.g., elevations in ALT, AST).

Note: ALT, AST, and total bilirubin must be performed within 3 days of ipilimumab and SGI-110 dosing. The results of these tests must be reviewed by the principal investigator (or designee) prior to dose administration.

6.3.7.2 Hematology

A CBC with differential is to be obtained as outlined in Table 6.1 The CBC with differential is to include: hemoglobin, hematocrit, white blood cells, platelets (direct platelet count), WBC differential enumeration of total and percentage of neutrophils, lymphocytes, eosinophils, basophils and monocytes. Additional draws must be incorporated when monitoring recovery from any hematologic AE.

CBC with differential must be performed within 3 days prior to ipilimumab and SGI-110 dosing. Results must be reviewed by principal investigator (or designee) prior to dose administration.

6.3.7.3 Urinalysis

A urinalysis will be obtained prior to first dosing and will include a gross examination including: specific gravity, protein, glucose and blood. A microscopic evaluation will also be performed, as needed, to include WBC/HPF, RBC/HPF and any additional findings. This test could be repeated later during the course of the study if clinically indicated.

6.3.7.4 HIV and Hepatitis Panel

At screening as outlined in Table 6.1, testing should be performed for HIV antibody, hepatitis C antibody and HBsAg utilizing local standard informed consent procedures prior to this laboratory collection. These tests could be repeated later during the course of the study if clinically indicated.

6.3.8 Tumor Tissue biopsy

Tumor tissue biopsy of cutaneous/subcutaneous lesions of at least 5 mm diameter will be performed for each subject at baseline, W4 and W12 as outlined in Table 6.1.

6.4 Efficacy Assessments

Data for the procedures and assessments specified in this protocol should be submitted to Fondazione NIBIT/CRO on a case report form. Additional procedures and assessments may be performed as part of standard of care; however, data for these assessments should remain in the subject's medical record and should not be provided to Fondazione NIBIT/CRO, unless specifically requested from the sponsor.

6.4.1 Primary Efficacy Assessment

The primary endpoint of this study is safety, tolerability, and Maximum Tolerated Dose (MTD) of SGI-110 in combination with ipilimumab in 21 day cycles in melanoma patients. Every effort should be made to collect, all safety data.

6.4.2 Secondary Efficacy Assessment

- To evaluate the immune-related (ir) -Disease Control Rate (ir-DCR), immune-related (ir) -Objective Response Rate (ir-ORR), immune-related (ir) -Time to Response (ir-TTR) and immune-related (ir) -Duration of Response (ir-DOR),
- To evaluate median immune-related (ir) Progression Free Survival (ir-PFS), median Overall Survival (OS), and survival rate at 1 and 2-years.

6.4.3 Exploratory Assessment

- To investigate immune-biologic correlates to treatment with SGI-110 in combination with ipilimumab **(see Appendix A**),
- Characterize pharmacokinetic profile of SGI-110 and decitabine. For a detailed workplan of pharmacokinetic analyses see Appendix B.

6.4.3.1 Radiologic Assessment of Tumor Lesions

CT scans with IV contrast and oral contrast of brain, neck, chest, abdomen, pelvis and ± soft tissue (or MRI, if iodine contrast media is medically contra-indicated) have to be performed for ALL patients at all Screening and at each tumor assessment visit. CT/MRI scans must be obtained of anatomic regions not covered by the chest, abdomen and pelvic scans, in subjects where there is clinical suspicion of deep soft tissues metastases (e.g., lesions in the thigh). Such additional CT/MRI scans will be required at Screening

only when deep soft tissue disease is known or suspected and must be consistently repeated at all tumor assessment visits if a deep soft tissue lesion is identified.

Similar methods of tumor assessment and similar techniques must be used to characterize each identified and reported lesion at Screening during the Treatment and Follow-up Phases. Response or progression of disease must be documented by a CT or an MRI similar to the methods used at Screening.

A reference measurement ruler must be printed on every image for scale determination. Sections should be contiguous, similarly sized and consistent from visit-to-visit. Section thickness must be based on institutional standards (e.g., from 5 to 8 mm, 10 mm cuts are not recommended). Chest x-rays and ultrasound are not acceptable methods to measure disease. Response or progression of disease must be documented by a CT or an MRI similar to the methods used at Screening.

1.1.1.1 Skin Lesions

Visible cutaneous lesions must be measured clinically. Assessment of skin lesions must be performed within close proximity (± 7 days) of any protocol-specified radiographic assessments for Treatment and Follow-up Phases.

6.4.3.2 Definition of Measurable/Non-measurable Lesions

Measurable Lesions:

Measurable lesions are lesions with clear borders that can be accurately measured in 2 dimensions with the following criteria:

- All measurements should be taken and recorded in centimeters,
- At Baseline the longest diameter and the longest perpendicular diameter must both be greater than or equal to 1.0 cm,
- For CT evaluations, the above definitions assume 5 mm contiguous slices. If slice thickness is greater than 5 mm, the above dimensions double,
- Cutaneous lesions should be photographed using a standard technique at each assessment point to document the clinical response.

Non-Measurable Lesions:

- Non-measurable lesions at baseline are all other lesions. These include small lesions (either or both diameter(s) <1.0 cm), bone lesions, leptomeningeal disease, ascites, pleural or pericardial effusions, inflammatory breast disease, lymphangitis cutis/pulmonis, or cystic lesions.

- Ideally, the same method of assessment and the same technique should be used to characterize each identified and reported lesion at baseline and during follow-up. The reader will determine the technical adequacy should the technique/modality change.
- Superficial and palpable-only lesions will be non-measurable for the radiologic portion of the independent review. These lesions will be considered as non-measurable during the IRC assessment unless there are no suitable radiographically measurable lesions. For this clinical assessment, cutaneous lesions should be photographed by a standard technique at each assessment point to document the clinical response. Copies of other imaging assessments, such as chest x-ray or ultrasound, which are not considered measurable for the radiologic assessment but measurable for the clinical assessment, will be kept centrally to document evidence of clinical response.

All measurable and non-measurable lesions should be assessed at Screening and at the defined TA time points (see Table 6.1). Extra assessments may be performed, as clinically indicated, if there is a suspicion of progression.

1.1.1.2 Definition of Index/Non-index Lesions

Measurable lesions, up to a maximum of 5 lesions per organ and 10 lesions in total, should be identified as index lesions to be measured and recorded on the CRF at Screening. The index lesions should be representative of all involved organs. In addition, index lesions must be selected based on their size (e.g., lesions with the longest diameters), their suitability for accurate repeat assessment by imaging techniques, and how representative they are of the subject's tumor burden. At Screening, a Sum of the Products of Diameters (SPD) for all index lesions will be calculated and considered the baseline SPD. The baseline sum will be used as the reference point to determine the objective tumor response of the index lesions at TA.

Measurable lesions, other than index lesions, and all sites of non-measurable disease, will be identified as non-index lesions. Non-index lesions will be recorded on the CRF and will be evaluated at the same assessment time points as the index lesions. In subsequent assessments, non-index lesions will be recorded as complete response, stable or progression.

Calculation of Sum of Product of Diameters (SPD)

SPD is an estimation of tumor burden. The 2 greatest perpendicular diameters are used to estimate the size of each tumor lesion. The SPD is calculated as the sum of the product of the diameters for index tumor lesions. Several variations of the SPD are identified for the purpose of classification of tumor responses.

SPD at Baseline: The sum of the product of the diameters for all index lesions identified at baseline prior to treatment on Day 1.

SPD at TA: For every on-study TA collected per protocol Section 6.1 or as clinically indicated, the SPD at TA will be calculated using tumor imaging scans.

SPD at Nadir: For tumors that are assessed more than one time after baseline, the lowest value of the SPD (SPD Baseline or SPD at TA) is used to classify subsequent TAs for each subject.

6.4.3.3 Definition of Tumor Response Using irRC

As stated in Section 4.1.2.1, when using the ir-response criteria, subjects are allowed to continue to receive study therapy despite radiologic evidence of progressive disease in index lesions, provided this is not accompanied by rapid clinical deterioration.

The SPD at TA using the irRC for progressive disease incorporates the contribution of new measurable lesions. Each net percentage change in tumor burden per assessment using irRC accounts for the size and growth kinetics of both old and new lesions as they appear. In this study the irRC as defined by the investigator will serve for basis of key endpoints and guide clinical care.

Definition of Index Lesions Response using irRC

- irComplete Response (irCR): Complete disappearance of all index lesions. This category encompasses exactly the same subjects as “CR” by the mWHO criteria.
- irPartial Response (irPR): Decrease, relative to baseline, of 50% or greater in the sum of the products of the 2 largest perpendicular diameters of all index and all new measurable lesions (ie, Percentage Change in Tumor Burden). Note: the appearance of new measurable lesions is factored into the overall tumor burden, but does not automatically qualify as progressive disease until the SPD increases by ≥ 25% when compared to SPD at nadir.
- irStable Disease (irSD): Does not meet criteria for irCR or irPR, in the absence of progressive disease.
- irProgressive Disease (irPD): At least 25% increase Percentage Change in Tumor Burden (i.e., taking sum of the products of all index lesions and any new lesions) when compared to SPD at nadir.

Definition of Non-Index Lesions Response using irRC

- irComplete Response (irCR): Complete disappearance of all non-index lesions. This category encompasses exactly the same subjects as “CR” by the mWHO criteria.
- irPartial Response (irPR) or irStable Disease (irSD): non-index lesion(s) are not considered in the definition of PR, these terms do not apply.
- irProgressive Disease (irPD): Increases in number or size of non-index lesion(s) does not constitute progressive disease unless/until the Percentage Change in Tumor Burden increases by 25% (ie, the SPD at nadir of the index lesions increases by the required amount).

Impact of New Lesions on irRC

New lesions alone do not qualify as progressive disease. However their contribution to total tumor burden is included in the SPD which in turn feeds into the irRC for tumor response. Therefore, new non-measurable lesions will not discontinue any subject from the study.

Definition of Overall Response Using irRC Will Be Based on the Following Criteria:

- Immune-related Complete Response (irCR): Complete disappearance of all tumor lesions (index and non-index together with no new measurable/unmeasurable lesions) for at least 4 weeks from the date of documentation of irCR.
- Immune-related Partial Response (irPR): The sum of the products of the 2 largest perpendicular diameters of all index lesions is measured and captured as the SPD baseline. At each subsequent TA, the sum of the products of the 2 largest perpendicular diameters of all index lesions and of new measurable lesions are added together to provide the Immune Response Sum of the Product of the Diameters (irSPD). A decrease, relative to baseline of the irSPD of 50% or greater is considered an irPR. It must be confirmed no less than 4 weeks from the first irPR.
- Immune-related Stable Disease (irSD): irSD is defined as the failure to meet criteria for immune complete response or immune partial response, in the absence of progressive disease.
- Immune-related Progressive Disease (irPD): Unless the subject is rapidly deteriorating, a confirmatory scan not less than 4 weeks since the prior scan should be performed to confirm PD. Any of the following will constitute progressive disease:
  - At least 25% increase in the SPD of all index lesions over nadir SPD calculated for these lesions.
  - At least a 25% increase in the SPD of all index lesions and new measurable lesions (irSPD) over the nadir SPD calculated for the index lesions.

Immune-related clinical activity (irCR, irPR or irSD) relative to baseline can be recorded at any time, including after confirmed irPD, provided that the patient has no received alternative non-ipilimumab anticancer therapy.

Table 6.4.1.6: ***irRC Definitions***

Index Lesion Definition	*Non-Index Lesion Definition*	*New Measurable Lesions*	*New Unmeasurable Lesion*	*% Change in Tumor Burden (including measurable new lesions when present)*	*Overall irRC Response*
Complete Response	***Complete Response***	***No***	***No***	***-100%***	***irCR***
Partial	***Any***	***Any***	***Any***	***≥-50%***	***irPR***
Response				***< -50% to < +25%***	***irSD***

Table 6.4.1.6: irRC Definitions

Index Lesion Definition	*Non-Index Lesion Definition*	*New Measurable Lesions*	*New Unmeasurable Lesion*	*% Change in Tumor Burden (including measurable new lesions when present)*	*Overall irRC Response*
				≥ + 25%	irPD
Stable Disease	Any	Any	Any	< -50% to < +25%	irSD
Stable Disease	Any	Any	Any	< -50% to < +25%	irSD
				≥ +25%	irPD
Progressive Disease	Any	Any	Any	≥ +25%	irPD

6.4.3.4 Derived Parameters When Using irRC Criteria

Immune Related Best Overall Response Using irRC (irBOR)

irBOR is the best irRC overall response over the study as a whole, recorded between the date of first dose until the last TA prior to subsequent therapy (including tumor resection surgery but excluding local palliative radiotherapy for painful bone lesions) for the individual subject in the study. For the assessment of irBOR, all available assessments per subject are considered. irCR or irPR determinations included in the irBOR assessment must be confirmed by a second (confirmatory) evaluation meeting the criteria for response and performed no less than 4 weeks after the criteria for response are first met.

irBOR assessment of irSD requires an overall response of irSD (or unconfirmed irPR or irCR), in the absence of irBOR of irCR, irPR or irPD.

6.5 Pharmacokinetic Assessments

Cycle 1, Day 1 with the following time-points: pre-dose, 15 min, 30 min, 60 min, 90 min, 2 hr, 4 hr, 6 hr and 8 hr post-dose. For a detailed workplan of pharmacokinetic analyses see **Appendix B.**

6.6 Translational Research

For a detailed work plan of translational studies see Appendix A

7 ADVERSE EVENTS

7.1 Definitions

An Adverse Event (AE) is defined as any new untoward medical occurrence or worsening of a pre-existing medical condition in a patient or clinical investigation subject administered an investigational (medicinal) product and that does not necessarily have a causal relationship with this treatment. An AE can therefore be any unfavorable and unintended sign (including an abnormal laboratory finding, for example), symptom, or disease temporally associated with the use of investigational product, whether or not considered related to the investigational product.

7.1.1 Serious Adverse Events

A serious AE (SAE) is any untoward medical occurrence that at any dose:

- results in death,
- is life-threatening (defined as an event in which the subject was at risk of death at the time of the event; it does not refer to an event which hypothetically might have caused death if it were more severe),
- requires inpatient hospitalization or causes prolongation of existing hospitalization (see note below for exceptions),
- results in persistent or significant disability/incapacity,
- is a congenital anomaly/birth defect ,
- is an important medical event (defined as a medical event(s) that may not be immediately life-threatening or result in death or hospitalization but, based upon appropriate medical and scientific judgment, may jeopardize the subject or may require intervention [e.g., medical, surgical] to prevent one of the other serious outcomes listed in the definition above.) Examples of such events include, but are not limited to, intensive treatment in an emergency room or at home for allergic bronchospasm; blood dyscrasias or convulsions that do not result in hospitalization).

Suspected transmission of an infectious agent (e.g., any organism, virus or infectious particle, pathogenic or non-pathogenic) via the study drug is an SAE and must be reported accordingly.

Although overdose and cancer are not always serious by regulatory definition, these events should be reported on an SAE form and sent to Fondazione NIBIT/CRO that will forward to BMS in an expedited manner.

All pregnancies, regardless of outcome, must be reported to the sponsor on a Pregnancy Surveillance Form, not an SAE form (see Section 7.7).

NOTE:

The following hospitalizations are not considered SAEs in Fondazione NIBIT clinical studies:

- a visit to the emergency room or other hospital department < 24 hours, that does not result in admission (unless considered "important medical event" or event life threatening),
- elective surgery, planned prior to signing consent,
- admissions as per protocol for a planned medical/surgical procedure,
- routine health assessment requiring admission for baseline/trending of health status (e.g., routine colonoscopy),
- medical/surgical admission for purpose other than remedying ill health state and was planned prior to entry into the study. Appropriate documentation is required in these cases,
- admission encountered for another life circumstance that carries no bearing on health status and requires no medical/surgical intervention (e.g., lack of housing, economic inadequacy, care-giver respite, family circumstances, administrative),
- Death due to disease progression.

7.1.2 Non serious Adverse Events

All AEs that are not classified as serious.

7.2 Dose-limiting Toxicities

Dose-limiting toxicities are defined in Section 5.3.

All presumed DLTs (as defined in Section 5.3) must be recorded immediately, i.e., within a maximum of 24 hours after becoming aware of the event, in the eCRF irrespective of seriousness and the event must be specified as a DLT. In addition, serious DLTs must be reported in an expedited manner according to the procedure for SAEs, as outlined above. Unless an NCI-CTCAE Grade 4 hematologic or a Grade 3/4 non-hematologic toxicity that occurs during the DLT observation period is clearly due to a non-drug cause, the Investigator should consider all AEs seen during the DLT observation period (from Day 1 to Day 21 of Cycle 1) to be at least possibly related to the trial treatment. In addition, the methods described in Section 6.3.5 and 7.4 should be used to assess all AEs throughout the trial until after completion of the SFU Visit(s).

Section 4.1.3 outlines the responsibilities of the SMC with respect to DLTs.

7.3 Assignment of Adverse Event Intensity and Relationship to Study Drug

All adverse events, including those that are serious, will be graded according to the National Cancer CTCAE version 4.

The following categories and definitions of causal relationship to study drug as determined by a physician should be used for all NIBIT clinical study AEs:

- Related: There is a reasonable causal relationship to study drug administration and the AE
- Not related: There is not a reasonable causal relationship to study drug administration and the AE.

The expression "reasonable causal relationship" is meant to convey in general that there are facts (e.g., evidence such as de-challenge/re-challenge) or other arguments to suggest a positive causal relationship.

7.4 Collection and Reporting

Adverse events can be spontaneously reported or elicited during open-ended questioning, examination, or evaluation of a subject. (In order to prevent reporting bias, subjects should not be questioned regarding the specific occurrence of one or more AEs.)

If known, the diagnosis of the underlying illness or disorder should be recorded, rather than its individual symptoms. The following information should be captured for all AEs: onset, duration, intensity, seriousness, relationship to study drug, action taken, and treatment required. If treatment for the AE was administered, it should be recorded on the appropriate CRF page. The investigator shall supply the sponsor and Ethics Committee with any additional requested information, notably for reported deaths of subjects.

Completion of supplemental CRFs may be requested for AEs and/or laboratory abnormalities that are reported/identified during the course of the study.

7.4.1 Serious Adverse Events

Following the subject’s written consent to participate in the study, all SAEs must be collected, including those thought to be associated with protocol-specified procedures. All SAEs must be collected that occur within 70 days of discontinuation of dosing of the investigational products. If applicable, SAEs must be collected that relate to any later protocol-specified procedure (e.g., a follow-up skin biopsy).

The investigator should notify Fondazione NIBIT/CRO of any SAE occurring after these time periods that is believed to be related to study drug or protocol-specified procedure.

Serious adverse events, whether related or unrelated to study drug, must be recorded on the SAE page of the CRF and reported within 24 hours to Fondazione NIBIT (or designee) to comply with regulatory

requirements. An SAE report should be completed for any event where doubt exists regarding its status of seriousness.

All SAEs must be reported within 24 hours by confirmed facsimile transmission (fax) and mailing of the completed SAE page (top, white, original). In some instances where a facsimile machine is not available, overnight express mail may be used. If only limited information is initially available, follow-up reports are required. (Note: Follow-up SAE reports should include the same investigator term(s) initially reported.) In selected circumstances, the protocol may specify conditions that require additional telephone reporting. The SAE electronic CRF in the electronic data capture tool should not be used.

If the investigator believes that an SAE is not related to study drug, but is potentially related to the conditions of the study (such as withdrawal of previous therapy, or a complication of a study procedure), the relationship should be specified in the narrative section of the SAE page of the CRF.

If an ongoing SAE changes in its intensity or relationship to study drug, a follow-up SAE report should be sent within 24 hours to the sponsor. As follow-up information becomes available it should be sent within 24 hours using the same procedure used for transmitting the initial SAE report. All SAEs should be followed to resolution or stabilization.

Fondazione NIBIT/CRO should notify all SAE to Astex Farmacovigilance

SAE FACSIMILE TRANSMISSION:

Contact information for SAE Facsimile Transmission will be provided to each site at time of study initiation.

SAE MAILING ADDRESS:

Contact information for SAE mailing will be provided to each site at time of study initiation.

7.4.2 Handling of Expedited Safety Reports

In accordance with local regulations, Fondazione NIBIT/CRO will notify investigators of all SAEs that are suspected (related to the investigational product) and unexpected (i.e., not previously described in the Investigator Brochure). In the European Union (EU), an event meeting these criteria is termed a Suspected, Unexpected Serious Adverse Reaction (SUSAR). Investigator notification of these events will be in the form of an expedited safety report (ESR).

Other important findings which may be reported by the sponsor as an ESR include: increased frequency of a clinically significant expected SAE, an SAE considered associated with study procedures that could modify the conduct of the study, lack of efficacy that poses significant hazard to study subjects, clinically significant safety finding from a nonclinical (e.g., animal) study, important safety recommendations from a study data monitoring committee, or sponsor decision to end or temporarily halt a clinical study for safety reasons.

Upon receiving an ESR from Fondazione NIBIT/CRO, the investigator must review and retain the ESR with the Investigator Brochure. Where required by local regulations or when there is a central IRB/IEC for the study, the sponsor will submit the ESR to the appropriate IRB/IEC. The investigator and IRB/IEC will determine if the informed consent requires revision. The investigator should also comply with the IRB/IEC procedures for reporting any other safety information.

In addition, suspected serious adverse reactions (whether expected or unexpected) shall be reported by Fondazione NIBIT/CRO to the relevant competent health authorities in all concerned countries according to local regulations (either as expedited and/or in aggregate reports). The Fondazione NIBIT/CRO will also report all suspected serious adverse reactions to Astex Farmacovigilance, within 24 hours from being informed by study Investigators.

7.4.3 Non serious Adverse Events

The collection of non-serious AE information should begin after patient's signed informed consent has been obtained . Non-serious AE information should also be collected from the start of a placebo lead-in period or other observational period intended to establish a baseline status for the subjects.

If an ongoing non-serious AE worsens in its intensity or its relationship to the study drug changes, a new non-serious AE entry for the event should be completed. Non-serious AEs should be followed to resolution or stabilization, or reported as SAEs if they become serious (see Section 7.1.1). Follow-up is also required for non-serious AEs that cause interruption or discontinuation of study drug, or those that are present at the end of study treatment as appropriate.

All identified non-serious AEs must be recorded and described on the appropriate non serious AE page of the CRF (paper or electronic).

7.5 Laboratory Test Abnormalities

All laboratory test values captured as part of the study should be recorded on the appropriate laboratory test results pages of the CRF, or be submitted electronically from a central laboratory. In addition, the following laboratory abnormalities should also be captured on the non-serious AE CRF page (paper or electronic) or SAE paper CRF page as appropriate:

- Any laboratory test result that is clinically significant or meets the definition of an SAE
- Any laboratory abnormality that required the subject to have study drug discontinued or interrupted
- Any laboratory abnormality that required the subject to receive specific corrective therapy

It is expected that wherever possible, the clinical, rather than the laboratory term would be used by the reporting investigator (e.g., anemia versus low hemoglobin value).

7.6 Overdose

An overdose is defined as the accidental or intentional ingestion or infusion of any dose of a product that is considered both excessive and medically important. All occurrences of overdose must be reported as an SAE (see Section 7.4.1 for reporting details.)

7.7 Pregnancy

Sexually active WOCBP must use an effective method of birth control during the course of the study, in a manner such that risk of failure is minimized (See Section 4.2.1 for the definition of WOCBP).

Before enrolling WOCBP in this clinical study, investigators must review the sponsor-provided information about study participation for WOCBP. The topics include the following:

- General Information
- Informed Consent Form
- Pregnancy Prevention Information Sheet
- Drug Interactions with Hormonal Contraceptives
- Contraceptives in Current Use
- Guidelines for the Follow-up of a Reported Pregnancy

Prior to study enrollment, WOCBP must be advised of the importance of avoiding pregnancy during study participation and the potential risk factors for an unintentional pregnancy. The subject must sign an informed consent form documenting this discussion.

7.7.1 Requirements for Pregnancy Testing

All WOCBP MUST have a negative pregnancy test within 72 hours as specified in Section 6.1 prior to receiving the investigational product. The minimum sensitivity of the pregnancy test must be 25 IU/L or equivalent units of HCG. If the pregnancy test is positive, the subject must not receive the investigational product and must not continue in the study.

Pregnancy testing must also be performed throughout the study as specified in Section 6.1 (see flow chart/time and events schedule) and the results of all pregnancy tests (positive or negative) recorded on the CRF or transferred electronically.

In addition, all WOCBP should be instructed to contact the investigator immediately if they suspect they might be pregnant (e.g., missed or late menstrual period) at any time during study participation.

7.7.2 Reporting of Pregnancy

If, following initiation of the investigational product, it is subsequently discovered that a study subject is pregnant or may have been pregnant at the time of investigational product exposure, including during at least

6 half-lives after product administration, the investigational product will be permanently discontinued in an appropriate manner (eg, dose tapering if necessary for subject safety). The investigator must immediately notify the Fondazione NIBIT/CRO of this event, record the pregnancy on the Pregnancy Surveillance Form (not an SAE form). Initial information on a pregnancy must be reported immediately to Fondazione NIBIT/CRO and the outcome information provided once the outcome is known. Completed Pregnancy Surveillance Forms must be forwarded to Fondazione NIBIT/CRO according to SAE reporting procedures described in Section 7.4.1.

Protocol-required procedures for study discontinuation and follow-up must be performed on the subject unless contraindicated by pregnancy (e.g., x-ray studies). Other appropriate pregnancy follow-up procedures should be considered if indicated. Follow-up information regarding the course of the pregnancy, including perinatal and neonatal outcome must be reported on the Pregnancy Surveillance Form.

Any pregnancy that occurs in a female partner of a male study participant should be reported to the sponsor. Information on this pregnancy will be collected on the Pregnancy Surveillance Form.

7.8 Other Safety Considerations

Any significant worsening noted during interim or final physical examinations, electrocardiograms, x-rays, and any other potential safety assessments, whether or not these procedures are required by the protocol, should also be recorded on the appropriate non serious AE page of the CRF (paper or electronic) or SAE paper CRF page.

8 STATISTICAL CONSIDERATIONS

8.1 Sample Size Determination

The primary endpoint of the study will be the Maximum Tolerated Dose (MTD) and safety of the combination of SGI-110 plus ipilimumab.

Three patients will be treated at the first dose level for one cycle.

Dose escalation will be dependent on the available toxicity information (including adverse events that are not DLTs), PK, and efficacy information, as well as the recommendations from the scientific committee

The dose escalation of SGI-110 will follow a traditional 3+3 design Cohorts of 3-6 patients will receive a combination therapy of SGI-110 and Ipilimumab 3mg/kg. Each patient will receive SGI-110 sc. for 5 consecutive days (D1-5) during a 3-week cycle at one of the following doses:

- Dose Level -1: 15 mg/m^2 day
- Dose Level 0: 30 mg/m^2 day

- Dose Level +1: 45 mg/m^2 day
- Dose Level +2: 60 mg/m^2 day

In the absence of dose-limiting toxicities (DLTs), the starting dose of SGI-110 of 30 mg/m^2 day will be escalated to 45 mg/m^2 daily on Days 1-5. In the absence of DLT in the Dose Level +1, the dose of SG1-110 of 45 mg/m^2 day will be escalated to 60 mg/m^2 day on Days 1-5 (Level +2). In the presence of 1 DLT in 3 subjects of the single dose cohort, the cohort will enroll 3 more subjects at the same dose level. In the event that 2 or more DLTs were encountered at the single dose cohort, the previous lower dose level will be evaluated. The MTD will be the highest SGI-110 dose (15-60 mg/m^2 day on Days 1-5) level in combination with ipilimumab at which no more than 1 of 6 patients experiences a DLT. Once the MTD is established, additional subjects will be treated at that dose up to a total of 19 subjects in the entire study.

Time to events will be estimated using Kaplan-Meier Product Limit method, survival rates and median survival will be reported with their corresponding two-sided 95% confidence intervals for each arm and compared using a 2-tailed log-rank test.

Cox Proportional hazard model will be used to estimate Hazard Ratio and its 95% confidence interval and to adjust estimation for baseline prognostic factors.

Toxicity will be reported according to National Cancer Institute-Common Toxicity Criteria (NCI CTC) version 4.0 (2004) criteria. The safety analysis population will include all subjects who receive at least one dose of drug. Subjects will be assigned to treatment groups as treated. In tables showing the overall incidence of AEs, subjects who experienced the same event on more than one occasion are counted only once in the calculation of the event frequency.

All safety parameters will be summarized and presented in tables based on this safety population.

Demographic and baseline characteristics will be summarized for all treated subjects using descriptive statistics. Reporting of safety, extent of exposure, concomitant medications and discontinuation of study therapy will be based on all treated subjects. Worst toxicity grades per subject will be tabulated for AEs and on-study laboratory measurements by using the National Cancer Institute (NCI) Common Terminology Criteria for Adverse Events (CTCAE) version 4.0.

- Translational studies
- For a detailed work plan of translational studies see **Appendix A**

8.2 Populations for Analyses

All treated subjects: All subjects who received at least one dose of SGI-110 and ipilimumab. (Full Analysis Population-FAS).

8.3 Endpoint Definitions

8.3.1 Primary Endpoint

The primary objective is to determine the MTD and safety profile of SGI-110 in combination with ipilimumab in 21 day cycles in melanoma patients. Endpoints related to this objective include an evaluation of DLTs, overall safety and parameters related to the MTD. The MTD evaluation will be based on the DLT evaluable population. Endpoints for safety profile include an evaluation of AEs, SAEs, laboratory evaluations, vital signs and physical examination.

8.3.2 Secondary Endpoints

8.3.2.1 Secondary Efficacy Endpoints based on immune-related Response Criteria

Tumor assessment, using the immune-related response criteria (ir-RC), is made by the investigator.

Immune-related Disease Control Rate (irDCR) is the proportion of treated subjects with an ir-BOR of confirmed irCR, confirmed irPR or irSD.

Immune-related Objective Response Rate (irORR) is the proportion of treated subjects with a irBOR of confirmed irCR or confirmed irPR.

Immune-related Time to Response (irTTR) is defined as the time from first dosing date until the measurement criteria are first met for overall response of irPR or irCR (whichever status comes first, and provided it is subsequently confirmed).

Immune-related Duration of Response (irDoR) for the subjects whose irBOR is irCR or irPR will be defined as the time between the date of response of confirmed irCR or confirmed irPR (whichever occurs first) and the date of irPD or death (whichever occurs first). The onset of a confirmed irCR or irPR is determined by the initial assessment of response, not by the confirmatory assessment. Note that if an assessment of irPR occurs before confirmation of irCR, the duration of immune-related response endpoint will not begin at the time that the irBOR of irCR is shown but rather at the earlier time-point showing irPR. For subjects who remain alive and have not progressed following response, irDoR will be censored on the date of last evaluable TA.

8.3.2.2 Immune-related Progression-free Survival (irPFS)

Immune-related progression free survival (irPFS) per irRC will be defined as the time between the date of randomization and the date of progression per irRC or death, whichever occurs first. A subject who dies without reported progression per irRC will be considered to have progressed on the date of death. For those subjects who remain alive and have not progressed, irPFS will be censored on the date of last evaluable TA. For those subjects who remain alive and have no recorded post baseline TA, irPFS will be censored on the day of last clinical evaluation.

8.3.3 Exploratory Endpoints

Translational Research

- For a detailed workplan of translational studies see **Appendix A,**
- The analysis of these data will be performed with a prospective exploratory intent. No formal hypotheses testing is planned,
- Characterize the pharmacokinetic profile of SGI-110 and decitabine. For a detailed workplan of pharmacokinetic analyses see **Appendix B.**

8.4 Analyses

8.4.1 Demographics and Baseline Characteristics

Demographic and baseline characteristics will be summarized for all subjects using descriptive statistics by treatment arms and overall for the FAS set. Categorical data will be presented as frequencies and percentages. For continuous data, mean, standard deviation, median, 25th and 75th percentiles, minimum and maximum will be reported.

8.4.2 Safety Analyses

Reporting of safety, extent of exposure, concomitant medications and discontinuation of study therapy will be based on all treated subjects; for on-study laboratory test results, all treated subjects with at least one on-study laboratory measurement available will be included in the analysis. The reporting period for safety data will be from the date of first dose received on this study to 70 days (5 half-lives) after the last dose is received. Serious adverse events are reported from the time of consent forward for all subjects.

The analysis of safety will be based on the frequency of AEs and their severity for all treated subjects. Worst toxicity grades per subject will be tabulated for AEs and on-study laboratory measurements by using the NCI CTCAE version 4.0. Additionally, immune-related (ir) see Section 1.3.5.3, drug-related and serious AEs will be tabulated separately, and listings by subject will be produced for all deaths, all SAEs and all AEs leading to discontinuation of study drug.

Assessment of extent of exposure will include tabulation of dose omissions, reductions and delays.

8.4.3 Efficacy Analyses

Analyses of efficacy endpoints will be based on all treated subjects (FAS set). The secondary efficacy endpoints will be evaluated with an explorative intent. The efficacy endpoints ir-DOR, ir-TTR will only be estimated for subjects with a confirmed best overall response of CR or PR. Exact two-sided 95% Confidence Intervals (CI) will be calculated using the binomial method for the proportion endpoints ir-DCR, ir-ORR.

OS will be estimated using Kaplan-Meier Product Limit method. A two-sided 95% confidence interval for the median survival will be computed using the Brookmeyer and Crowley method. PFS, ir-DOR, and Brain-PFS will be analyzed similarly.

OS will be further described using the survival rate, defined as the probability that a subject is alive at 6, 12, 18 and 24 months following date of the first treatment dose and estimated via the Kaplan-Meier method. A corresponding two-sided 95% confidence interval will be calculated.

Ir-TTR will be summarized using descriptive statistics.

A Cox regression analysis will be performed to adjust treatment effect for prognostic factors. Prognostic factors will be identified, before analysis, in the Statistical Analysis Plan.

Pharmacokinetic Analyses

Characterize the pharmacokinetic profile of SGI-110 and decitabine. For a detailed workplan of pharmacokinetic analyses see **Appendix B.**

8.4.4 Translational Research

For a detailed workplan of translational studies see **Appendix A.**

8.5 Interim Analyses

No interim analysis for efficacy will be conducted.

9 ADMINISTRATIVE SECTION

9.1 Compliance

9.1.1 Compliance with the Protocol and Protocol Revisions

The study shall be conducted as described in this approved protocol. All revisions to the protocol must be discussed with, and be prepared by, Fondazione NIBIT. The investigator should not implement any deviation or change to the protocol without prior review and documented approval/favorable opinion from the IRB/IEC of an amendment, except where necessary to eliminate an immediate hazard(s) to study subjects. Any significant deviation must be documented in the CRF.

If a deviation or change to a protocol is implemented to eliminate an immediate hazard(s) prior to obtaining IRB/IEC approval/favorable opinion, as soon as possible the deviation or change will be submitted to:

- IRB/IEC for review and approval/favorable opinion,
- Fondazione NIBIT/CRO,

Regulatory Authority(ies), if required by local regulations,

Documentation of approval signed by the chairperson or designee of the IRB(s)/IEC(s) must be sent to Fondazione NIBIT/CRO.

If an amendment substantially alters the study design or increases the potential risk to the subject: (1) the consent form must be revised and submitted to the IRB(s)/IEC(s) for review and approval/favorable opinion; (2) the revised form must be used to obtain consent from subjects currently enrolled in the study if they are affected by the amendment; and (3) the new form must be used to obtain consent from new subjects prior to enrollment.

If the revision is an administrative letter, investigators must inform their IRB(s)/IEC(s).

9.1.2 Monitoring

Representatives of Fondazione NIBIT/CRO must be allowed to visit all study site locations periodically to assess the data quality and study integrity. On site they will review study records and directly compare them with source documents, discuss the conduct of the study with the investigator, and verify that the facilities remain acceptable.

In addition, the study may be evaluated by Fondazione NIBIT and government inspectors who must be allowed access to CRFs, source documents, other study files, and study facilities. NIBIT audit reports will be kept confidential.

THE INVESTIGATOR MUST NOTIFY FONDAZIONE NIBIT/CRO PROMPTLY OF ANY INSPECTIONS SCHEDULED BY REGULATORY AUTHORITIES, AND PROMPTLY FORWARD COPIES OF INSPECTION REPORTS TO FONDAZIONE NIBIT/CRO.

9.1.3 Investigational Site Training

Fondazione NIBIT/CRO will provide quality investigational staff training prior to study initiation. Training topics will include but are not limited to: GCP, AE reporting, study details and procedure, study documentation, informed consent, and enrollment of WOCBP.

For sites using the Fondazione NIBIT/CRO electronic data capture tool, each individual making entries and/or corrections on electronic CRFs must meet Fondazione NIBIT/CRO training requirements and must only access the Fondazione NIBIT/CRO electronic data capture tool using the unique user account provided by the sponsor. User accounts are not to be shared or reassigned to other individuals.

For electronic CRFs, corrections are made through the Fondazione NIBIT/CRO electronic data capture tool that generates an automated audit trail including date and timestamp, full name of the person making the correction and original entry. The system also prompts the user to document reason for change that is also maintained in the audit trail.

Each individual electronically signing electronic CRFs must meet Fondazione NIBIT/CRO training requirements and must only access the Fondazione NIBIT/CRO electronic data capture tool using the unique user account provided by the sponsor. User accounts are not to be shared or reassigned to other individuals.

9.2 Records Retention

The investigator must retain study drug (those supplied by the sponsor or sourced by the investigator) disposition records, copies of CRFs (or electronic files), and source documents for the maximum period required by applicable regulations and guidelines, or institution procedures, or for the period specified by the sponsor, whichever is longer. The investigator must contact Fondazione NIBIT/CRO prior to destroying any records associated with the study.

NIBIT/CRO will notify the investigator when the study records are no longer needed.

If the investigator withdraws from the study (e.g., relocation, retirement), the records shall be transferred to a mutually agreed upon designee (e.g., another investigator, IRB). Notice of such transfer will be given in writing to Fondazione NIBIT/CRO.

9.2.1 Case Report Forms

An investigator is required to prepare and maintain adequate and accurate case histories designed to record all observations and other data pertinent to the investigation on each individual treated or entered as a control in the investigation. Data reported on the CRF that are derived from source documents must be consistent with the source documents or the discrepancies must be explained.

For sites using the Fondazione NIBIT/CRO electronic data capture tool, electronic CRFs will be prepared for all data collection fields except for fields specific to SAEs and pregnancy, which will be reported on the Pregnancy Surveillance Form.

Paper CRFs must be completed legibly in ink. Subjects are to be identified by birth date and subject number, if applicable. All requested information must be entered on the CRF in the spaces provided. If an item is not available or is not applicable, it must be documented as such; do not leave a space blank.

The confidentiality of records that could identify subjects must be protected, respecting the privacy and confidentiality rules in accordance with the applicable regulatory requirement(s).

The investigator will maintain a signature sheet to document signatures and initials of all persons authorized to make entries and/or corrections on CRFs.

For paper CRFs, a correction must be made by striking through the incorrect entry with a single line and entering the correct information adjacent to the incorrect entry. The correction must be dated, initialled and explained (if necessary) by the person making the correction and must not obscure the original entry.

The completed CRF, including any paper SAE/pregnancy CRFs, must be promptly reviewed, signed, and dated by a qualified physician who is an investigator or sub-investigator. For electronic CRFs, review and approval/signature is completed electronically through the Fondazione NIBIT/CRO electronic data capture tool. The investigator must retain a copy of the CRFs including records of the changes and corrections.

9.2.2 Study Drug Records

It is the responsibility of the investigator to ensure that a current disposition record of investigational product (those supplied by the sponsor) is maintained at each study site where study drug is inventoried and disposed. Records or logs must comply with applicable regulations and guidelines and should include:

- amount received and placed in storage area
- amount currently in storage area
- label ID number or batch number and use date
- dates and initials of person responsible for the inventory/ entry/ movement of each study drug
- amount dispensed to and returned by each subject, including unique subject identifiers
- amount transferred to another area/site for dispensing or storage
- non-study disposition (e.g., lost, wasted, broken)
- amount returned to the sponsor
- amount destroyed at study site, if applicable
- retain samples sent to third party for bioavailability/bioequivalence, if applicable

The sponsor will provide forms to facilitate inventory control if the staff at the investigational site does not have an established system that meets these requirements.

9.3 Destruction of Study Drug

9.3.1 Destruction of Study Drug

If study drugs (those supplied by the sponsor or sourced by the investigator) are to be destroyed on site, it is the investigator's responsibility to ensure that arrangements have been made for the disposal, written authorization has been granted by Fondazione NIBIT/CRO, procedures for proper disposal have been established according to applicable regulation and guidelines and institutional procedures, and appropriate records of the disposal have been documented. The unused study drugs can only be destroyed after being inspected and reconciled by the responsible Fondazione NIBIT/CRO Study Monitor.

9.4 Publications

The data collected during this study are confidential and proprietary to the sponsor. Any publications or abstracts arising from this study require approval by the sponsor prior to publication or presentation and must adhere to the sponsor's publication requirements as set forth in the approved clinical trial agreement (CTA). All draft publications, including abstracts or detailed summaries of any proposed presentations, must be submitted to the sponsor at the earliest practicable time for review, but at any event not less than 30 days before submission or presentation unless otherwise set forth in the CTA. Sponsor shall have the right to delete any confidential or proprietary information contained in any proposed presentation or abstract and may delay publication for up to 60 days for purposes of filing a patent application.

ADDITIONAL ETHICAL CONSIDERATIONS

APPENDIX 1

1 INFORMED CONSENT PROCEDURES

NIBIT/CRO will provide the investigator with an appropriate (i.e., Global or Local) sample informed consent form that will include all elements required by ICH, GCP and applicable regulatory requirements. The sample informed consent form will adhere to the ethical principles that have their origin in the Declaration of Helsinki. If the investigator makes changes to the informed consent form sample, NIBIT/CRO will ensure all required elements and local regulatory and legal requirements are met.

The consent form must also include a statement that NIBIT/CRO and regulatory authorities have direct access to subject records. Prior to the beginning of the study, the investigator must have the IRB/IEC's written approval/favourable opinion of the written informed consent form and any other information to be provided to the subjects.

The investigator must provide the subject, or, in those situations where consent cannot be given by subjects, their legally acceptable representative with a copy of the consent form and written information about the study in the language in which the subject is most proficient. The language must be non-technical and easily understood. The investigator should allow time necessary for subject or subject's legally acceptable representative to inquire about the details of the study, then informed consent must be signed and personally dated by the subject or the subject's legally acceptable representative and by the person who conducted the informed consent discussion. The subject or legally acceptable representative should receive a copy of the signed informed consent and any other written information provided to study subjects prior to subject's participation in the study.

1.1 Subjects Unable to Give Written Informed Consent

1.1.1 Minors (Not Applicable)

For minors, according to local legislation, one or both parents or a legally acceptable representative must be informed of the study procedures and must sign the informed consent form approved for the study prior to clinical study participation. (In the event that the parents or legal guardians are unable to read, then an impartial witness should be present during the entire informed consent discussion). Whenever feasible, minors who are judged to be of an age of reason must also give their written assent by signing and dating the

completed informed consent. All local laws, rules and regulations regarding informed consent of minors must be followed.

1.1.2 Subjects Experiencing Acute Events or Emergencies

A legally acceptable representative or legal guardian must provide informed consent when consent of the subject is not possible prior to clinical study participation, e.g., for subjects experiencing an acute medical event such as myocardial infarction or stroke. Informed consent of the subject must additionally be obtained if they become capable of making and communicating their informed consent during the clinical study. All local laws, rules and regulations regarding informed consent of adult subjects incapable of giving informed consent must be followed.

1.1.3 Mentally Impaired or Incapacitated Subjects

Investigators (or whoever required by local regulations) should determine whether or not a mentally impaired or incapacitated subject is capable of giving informed consent and should sign a statement to that effect. If the subject is deemed mentally competent to give informed consent, the investigator should follow standard procedures. If the subject is deemed not to be mentally competent to give informed consent, a fully informed legal guardian or legally acceptable representative can be asked to give consent for, or on behalf of, the subject. All local laws, rules and regulations regarding informed consent of mentally impaired or incapacitated subjects must be followed.

Patients who are involuntarily hospitalized because of mental illness must not be enrolled in clinical studies.

1.1.4 Other Circumstances

Subjects who are imprisoned or involuntarily detained for treatment of either a psychiatric or physical (e.g., infectious disease) illness must not be enrolled in clinical studies. In circumstances where a subject's only access to treatment is through enrollment in a clinical study, e.g., for subjects in developing countries with limited resources or for subjects with no marketed treatment options, the investigator must take special care to explain the potential risks and benefits associated with the study and ensure that the subject is giving informed consent. When a subject may be in a dependent relationship with the investigator, a well-informed physician who is not engaged in the clinical study and is completely independent of the relationship between the subject and investigator should obtain the subject's informed consent.

1.1.5 Illiterate Subjects

If the subject, or, in those situations where consent cannot be given by the subject, their legally acceptable representative is unable to read, a reliable and independent witness should be present during the entire informed consent discussion. The choice of the witness must not breach the subject's rights to

confidentiality. A reliable independent witness is defined as one not affiliated with the institution or engaged in the investigation. A family member or acquaintance is an appropriate independent witness. After the subject or legally acceptable representative orally consents and has signed, if capable, the witness should sign and personally date the consent form attesting that the information is accurate and that the subject, or, in those situations where consent cannot be given by subjects, their legally acceptable representative has fully understood the content of the informed consent agreement and is giving true informed consent.

1.2 Update of Informed Consent

The informed consent and any other information provided to subjects, or, in those situations where consent cannot be given by subjects, the subject's legally acceptable representative, should be revised whenever important new information becomes available that is relevant to the subject's consent, and should receive IRB/IEC approval/favourable opinion prior to use. The investigator, or a person designated by the investigator should fully inform the subject or the subject's legally acceptable representative of all pertinent aspects of the study and of any new information relevant to the subject's willingness to continue participation in the study. This communication should be documented. During a subject's participation in the study, any updates to the consent form and any updates to the written information will be provided to the subject.

GLOSSARY OF TERMS

Term	*Definition*
**Adverse Reaction**	An adverse event that is considered by either the investigator or the sponsor as related to the investigational product
**Expedited Safety Report**	Rapid notification to investigators of all SAEs that are suspected (related to the investigational product) and unexpected (ie, not previously described in the Investigator Brochure), or that could be associated with the study procedures.
**SUSAR**	Suspected, Unexpected, Serious Adverse Reaction as termed by the European Clinical Trial Directive (2001/20/EC).
**Unexpected Adverse Reaction**	An adverse reaction, the nature or severity of which is not consistent with the applicable product information (e.g., Investigator Brochure for an unapproved investigational product)

List of Abbreviations

Term	*Definition*
Aes	Adverse Events
ALT	Serum Glutamate Pyruvate Transaminase (SGPT)
ANC	Absolute Neutrophil Count
AST	Aspartate Transaminase (SGOT)
BA/BE	Bioavailability / Bioequivalence
BMS	Bristol-Myers Squibb
BOR	Best Overall Response
BP	Blood Pressure
BSA	Body Surface Area
BUN	Urea Nitrogen
C	Celsius
CBC	Complete Blood Count
CHO cell	Chinese Hamster Ovary
CI	Confidence Interval
CNS	Central Nervous System
CR	Complete Response
CRF	Case Report Form, paper or electronic
CRO	Contract Research Organization
CT	Computed Tomography
CTCAE	Common Terminology Criteria for Adverse Events
CTLA-4	Cytotoxic T-Lymphocyte Associated Protein 4
CV	Coefficient of Variation
D	Day
DCR	Disease Control Rate
DOR	Duration of Response
DTIC	Dacarbazine
ECG	Electrocardiogram
ECOG	Eastern Cooperative Oncology Group
Eg	For example
ESR	Expedited Safety Report
FDA	Food and Drug Administration
FSH	Follicle Stimulating Hormone
G	Gram
g/dL	Grams per Deciliter
GI	Gastrointestinal
GM	Granulocyte-macrophage
GM-CSF	Granulocyte-macrophage colony stimulating factor
H	Hour
HAHA	Human Anti-Human Antibody
HbsAg	Hepatitis B Surface Antigen
HCG	Human Chorionic Gonadotropin

Term	*Definition*
HCO3	Bicarbonate
HepB	Hepatitis B
HepC	Hepatitis C
HIV	Human Immunodeficiency Virus
HLA-A*0201	Human Leukocyte Antigen or Human Lymphocyte Antigen
HRT	Hormone Replacement Therapy
IB	Investigator Brochure
ICH GCP	International Conference on Harmonization Good Clinical Practice
ICU	Intensive Care Unit
Ie	That is
IgG1	Immunoglobulin G
IL-1	Interleukin-1
IL-2	Interleukin-2
IP	Investigational Product
ir	Immune-related
IRB/IEC	Institutional Review Board / Independent Ethics Committee
IRC	Independent Review Committee
IV	Intravenous
K3EDTA	Potassium ethylene-diamine tetra-acetic acid
Kg	Kilogram
LDH	Lactate dehydrogenase
LFT	Liver Function Test
m	Minute
MDDCR	Major Durable Disease Control Rate
mg	Milligram
ml	Milliliter
mL	Monocyte to lymphocyte (ratio)
mm	Millimeter
mmHg	Millimeters of Mercury
MRI	Magnetic Resonance Imaging
mRNA	Messenger Ribonucleic Acid
mWHO	Modified World Health Organization
NC	No Change
NCI	National Cancer Institute
NK	Natural Killer
ORR	Objective Response Rate
OS	Overall Survival
PD	Progressive Disease
PE	Physical Examination
PFS	Progression Free Survival
PHA-	Phytohemagglutinin
PK	Pharmacokinetics

Term	*Definition*
PR	Partial Response
PR/CR	Partial Response/Complete Response
Pt	Patient
Q	Every
irRC	Immune related Response Criteria
RBC	Red Blood Count
RBC/HPF	Red Blood Cells / High Powered Field
RF	Rheumatoid Factor
RR	Response Rate
QLQ-C30	Quality of Life Questionnaire C30
SAEs	Serious Adverse Events
SD	Stable Disease
SGOT	Aspartate Transaminase (AST)
SGPT	Serum Glutamate Pyruvate Transaminase (ALT)
SmPC	Summary of Product Characteristics
SPD	Sum of Products of the Diameters
TA	Tumor Assessment
TNF	Tumor Necrosis Factor
TNM	Melanoma Tumor Staging (i.e., Thickness, # of Metastatic Nodes and Site)
TTR	Time to Response
ULN	Upper Limit of Normal
US	United States
Vss	Apparent Volume of Distribution
WBC	White Blood Cells
WBC/HPF	White Blood Cells / High Powered Field
Wk	Week
WOCBP	Women of Childbearing Potential

REFERENCES

1. Hodi FS, O'Day SJ, McDermott DF, Weber RW, Sosman JA, Haanen JB et al. Improved survival with ipilimumab in patients with metastatic melanoma. *N Engl J Med* 2010;363(8):711-723.
2. Maio M, Di Giacomo AM, Robert C, Eggermont AM. Update on the role of ipilimumab in melanoma and first data on new combination therapies. *Curr Opin Oncol* 2013;25(2):166-172.
3. Gore SD, Hermes-DeSantis ER. Enhancing survival outcomes in the management of patients with higher-risk myelodysplastic syndromes. Cancer Control. 2009 Oct;16 Suppl:2-10. Review
4. Steensma DP, Porcher JC, Litzow MR, Hogan WJ, Arora S, Van Laar ES.Assessment of ATRX expression in patients with myelodysplastic syndromes treated with decitabine. Leuk Res. 2009 Jul;33(7):e81-2.
5. Yang AS, Estecio MR, Doshi K, Kondo Y, Tajara EH, Issa JP. A simple method for estimating global DNA methylation using bisulfite PCR of repetitive DNA elements. Nucleic Acids Res 2004;32(3):e38.
6. Kantarjian HM, Roboz GJ, Rizzieri DA, Stock W, O'Connell CL, Griffiths EA et al. Results From the Dose Escalation Phase of a Randomized Phase 1–2 First-in-Human (FIH) Study of SGI-110, a Novel Low Volume Stable Subcutaneous (SQ) Second Generation Hypomethylating Agent (HMA) in Patients with Relapsed/Refractory MDS and AML. *Blood* (ASH Annual Meeting Abstracts), Nov 2012; 120: 414.
7. Kantarjian HM, Oki Y, Garcia-Manero G, Huang X, O'Brien S, Cortes J et al. Results of a randomized study of 3 schedules of low-dose decitabine in higher-risk myelodysplastic syndrome and chronic myelomonocytic leukemia. *Blood* 2007;109(1):52-57.
8. Roboz GJ, Issa JP, Rizzieri D, Stock W, O'Connell C, Yee K et al. DNA demethylation activity over time and safety of 3 different dose-escalation regimens of SGI-110, a novel subcutaneous (SQ) hypomethylating agent (HMA), in the treatment of relapsed/refractory patients with MDS and AML. (ASH Annual Meeting Abstracts). *Blood*. 122(21):1548.
9. Kantarjian HM, Jabbour E, Yee K, Kropf P, O’Connell C, Stock W et al. First Clinical Results Of a Randomized Phase 2 Study Of SGI-110, a Novel Subcutaneous (SQ) Hypomethylating Agent (HMA), In Adult Patients With Acute Myeloid Leukemia (AML). *Blood* (ASH Annual Meeting Abstracts), Nov 2013 122:497
10. Yee K, Daver N, Kropf P, Tibes R, O'Connell C, Roboz G et al. Abstract 5647: Results of a randomized multicenter phase 2 study of a 5-day regimen of SGI-110, a novel hypomethylating agent, in treatment-naïve elderly acute myeloid leukemia not eligible for intensive therapy. Oral

presentation at 19th Congress of the European Hematology Association (EHA); Jun 12-15; Milan, Italy. EHA

11. Griffiths E, Kantarjian H, Roboz G, Kropf P, O'Connell C, Yee K et al. Abstract 3074: First results of a Phase 2 study using a 10-day subcutaneous (SC) regimen of the novel hypomethylating agent (HMA) SGI-110 for the treatment of relapsed/refractory acute myeloid leukemia (r/r AML). Proceedings of the 50th Annual Meeting of the American Society of Clinical Oncology (ASCO); May 30-June 3; Chicago, IL.
12. Maio M, Choy G, Covre A, Parisi G, Nicolay H, Fratta E et al. Immunomodulatory activity of SGI-110, a second generation hypomethylating agent. Proceedings of the 11th International Congress on Targeted Anticancer Therapies (TAT 2013), 4–6 March 2013, Paris, France. *Ann Oncol* 2013; 24 (suppl 1): i8-i9.
13. Covre A, Parisi G, Nicolay HJ, Fonsatti E, Fratta E, Sigalotti L et al. In vivo immunomodulatory activity of SGI-110, a second generation hypomethylating agent, in hematologic malignancies [Abstract]. Proceedings of the 104th Annual Meeting of the American Association for Cancer Research; 2013 Apr 6-10; Washington, DC. Philadelphia (PA): AACR; 2013.
14. Covre A, Coral S, Di Giacomo AM, Taverna P, Azab M, Maio M. Epigenetics meets immune checkpoints *Seminars inOncology*, Vol 42,No3,June2015,pp506-513 (in press)
15. Covre A, Parisi G, Nicolay H.J, Fazio C, Fonsatti E, Fratta E, Sigalotti L, Taverna P, Choy G, Azab M, Coral S, and Maio M. "Immunomodulatory activity of SGI-110: a basis for novel chemoimmuno therapeutic combinations in cancer treatment. SITC 2013 Annual Meeting and Associated Programs. Washington, November 7-10, 2013. *Journal for Immunotherapy of Cancer 2013* 1 (Suppl 1):P71
16. Covre A, Fazio C, H Nicolay H.J, Natali P.G, Taverna P, Azab M, Coral S, and Maio M. Epigenetic immunomodulation by SGI-110 combined with immune checkpoint blockade for new therapeutic strategies. 26th EORTC – NCI – AACR Symposium on Molecular Targets and Cancer Therapeutics. *European Journal of cancer* November 2014 Volume 50, Supplement 6, p3-239.
17. SGI-110 Investigator Brochure v.4 (22-Sep-2014)
18. Ipilimumab Investigator Brochure v.16 (13-Mar-2013)
19. Robert C, Thomas L, Bondarenko I, et al. Ipilimumab plus dacarbazine for previously untreated metastatic melanoma. N Engl J Med. 2011 Jun 30;364(26):2517-26.
20. Maio M, Immunologic checkpoints for cancer treatment: from scientific rationale to clinical application. Semin Oncol. 2010 Oct; Vol. 37, No 5
21. Wolchok JD, Weber JS, Maio M et al, Four-year survival rates for patients with metastatic melanoma who received ipilimumab in phase II clinical trials. Ann Oncol, 2013; 24:2174-2180)
22. Di Giacomo AM, Calabrò L, Danielli R, et al. Long-term survival and immunological parameters in metastatic melanoma patients who responded to ipilimumab 10 mg/kg within an expanded access programme. Cancer Immunol Immunother. 2013 Jun;62(6):1021-8.

23. Di Giacomo AM, Ascierto PA, Pilla L, et al. A phase 2, open-label, single-arm study to investigate the combination of ipilimumab and fotemustine in patients with advanced melanoma: the NIBIT-M1 trial. Lancet Oncol. 2012 Sep;13(9):879-86.

24. Sigalotti L, Fratta E, Coral S, Maio M. Epigenetic drugs as immunomodulators for combination therapies in solid tumors. Pharmacol Ther. 2013 Dec 30. pii: S0163-7258(13)00257-X. doi: 10.1016/j.pharmthera.2013.12.015. [Epub ahead of print]

25. Coral S, Sigalotti L., Gasparollo A., Cattarossi I., Visintin A., Altomonte M., and Maio M. Prolonged up-regulation of the expression of HLA class I antigens and co-stimulatory molecules on melanoma cells treated with 5-aza-2'-deoxycytidine (5-AZA-CdR). J Immunotherapy 1999, 22(1): 16-24.

26. Maio M. Immunologic effects of targeted therapies and chemotherapy. Second international Michelangelo conference: promises and challenges of developing new drugs in oncology. 4-5 July, 2013, Milan, Italy.

27. Coral S, Parisi G, Nicolay HJ, Colizzi F, Danielli R, Fratta E, Covre A, Taverna P, Sigalotti L, Maio M. Immunomodulatory activity of SGI-110, a 5-aza-2'-deoxycytidine-containing demethylating dinucleotide. Cancer Immunol Immunother 2013, Mar;62(3):605-614.

28. Covre A, Fazio C, Nicolay HJMG, Parisi G, Taverna P, Azab M, Coral S, and Maio M. “Epigenetic priming with novel hypomethylating agent SGI-110 improved antitumor activity of CTLA-4 blockade in a syngeneic mouse model”. AACR Molecular Targets and Cancer Therapeutics. October 19-24, 2013.

29. Matei D, Fang, F., Shen, C., *et al*., Epigenetic Resensitization to Platinum in Ovarian Cancer. Cancer Res, 2012. 72(9):2197-2205

30. Smith TJ, Khatcheressian J, Lyman GH, et al. 2006 update of recommendations for the use of white blood cell growth factors: an evidence-based clinical practice guideline. J Clin Oncol 2006;24:3187-3205.

APPENDIX A

CORRELATIVE STUDIES

Rationale: The main objective of the proposal is to evaluate and to elucidate immune-mediated mechanisms of clinical activities of CTLA-blockade combined with a DNA hypomethylating agent. To this aim, changes in the immune phenotype and epigenetic profile of neoplastic cells and of immune cells, as well as changes in the humoral and cellular immune responses induced by treatment will be investigated utilizing standardized and validated techniques. The results obtained from the characterization of tumor and host immunomodulation achieved by the treatment will be correlated with clinical parameters in order to identify correlates of disease outcome and/or new insights to improve the effectiveness of combined therapies with DNA hypomethylating agents and anti-CTLA-4 antibodies.

Biological samples required: 70 ml pf peripheral blood

Timepoint: blood samples will be collected at baseline, week 0,1,3,4,12 and 24, as outlined in Table 6.1.

Time required for the analysis: 1 year after the end of patients' enrolment.

1) Phenotypic and epigenetic profile of tumor samples

1a. When tumor biopsies will be available, the immune phenotype profile (tumor antigens, MHC molecules, immunomodulating and costimulatory molecules) of neoplastic lesions will be investigated by immunofluorescence and cytofuorimetric analysis, by immochemistry (IHC) and/or by RT-PCR assays.

1b. When tumor biopsies will be available, the hypomethylation status of Cancer Testis Antigens (CTA) promoters will be investigated on neoplastic lesions by quantitative methylation-specific polymerase chain reaction (MSP).

1c. When tumor biopsies will be available, the overall hypomethylation status of the genomic DNA of neoplastic lesions will be investigated by MSP.

1d. When tumor biopsies will be available, the immune infiltrates in neoplastic lesions will be characterized with specific monoclonal antibodies by IHC assays.

2) Phenotypic, functional and epigenetic profile of peripheral blood mononuclear cells (PBMC)

2a. Changes in the immune-phenotypic profile of PBMC from treated patients will be analyzed by multiparametric immune florescence analysis using specific monoclonal antibodies in order to characterize different T cell populations (TH1, TH2 and TH17) and in order to analyse different co-stimulatory molecules (CD28, CD27, NKG2D, OX40, 4-1BB, ICOS, LAG-3) and immune regulatory molecules (CTLA-4, PD-1, BTLA).

2b. The modulation of poly-functional activity of T-cells from treated patients will be evaluated by intracellular cytokines staining and/or multiparametric ELISPOT assay.

2c. The induction/modulation of cellular immune responses against a panel of CTA and/or well characterized melanoma cell lines induced by the treatment will be analyzed by cytokines release (ELISPOT assay) and/ or standard cytotoxicity assays.

2d. The hypomethylation of the overall genomic DNA of PBMC from treated patients will be investigated by pyrosequencing for LINE-1 methylation.

3) Humoral immune response

3a. The induction/up-regulation of circulating antibodies directed to anti-tumor-associated antigens (TAA) will be evaluated by ELISA in sera of treated patients utilizing a panel of recombinant proteins (HMW-MAA, CTA, Survivin, Melan-A/MART-1, Tyrosinase) commercially available or in house-produced.

3b. The induction/up-regulation of anti-CTA B-cell responses will be evaluated by testing the ability of sera collected from treated patients to mediate antibody-dependent cell-mediated cytotoxicity by ELISPOT assay or standard cytotoxicity assays.

3c. The modulation of circulating tumor DNA in treated patients will be evaluated in plasma of treated patients by Droplet Digital PCR or Beaming Digital PCR.

The studies at points 1a, 1b, 1c, 2b, 2c, 2d, 3b and 3c will be carried out at NIBIT Laboratories c/o Toscana Life Science Siena, Italy.

The studies at points 2a and 3a will be carried out at the U.O.C. Immunoterapia Oncologica of the Azienda Ospedaliera Universitaria Senese, Siena, Italy, (PI: Dr. E. Fonsatti) upon signing an ad hoc contract between NIBIT and the Azienda Ospedaliera Universitaria Senese.

APPENDIX B

SECTION 1. PHARMACOKINETIC STUDIES FOR SGI-110

Pharmacokinetic evaluation in study subjects will include to evaluation of exposures of SGI-110 and active metabolite decitabine in the disease population proposed.

Pharmacokinetics of SGI-110 and decitabine will be assessed from plasma concentrations.

Plasma samples will be prepared from blood drawn at the following time-points:

Cycle 1, Day 1: pre-dose, 15 min, 30 min, 60 min, 90 min, 2 hr, 4 hr, 6 hr and 8 hr post-dose.

Sampling windows are up to 10% of protocol specified time point but no more than 1 hour.

Important note: due to presence of cytidine deaminase in blood/plasma, addition of THU (tetrahydrouridine, an inhibitor of CDA) is required to prevent degradation of active metabolite decitabine after sample collection. Details for procedures for PK sample collection are included below. Plasma samples should be appropriately labeled and shipped for analysis.

Astex can provide kits for PK sample collection and will also arrange for analysis of PK samples.

Please contact Aram Oganesian (aram.oganesian@astx.com) for details on coordinating this work.

Detailed Plasma Collection Procedures:

A. Preparation of THU (tetrahydrouridine) for spiking EDTA blood collection tubes - Materials

THU: Calbiochem 584222, EMD Chemicals, Inc.

Notes and Preparation of THU:

1) The 10 mg vial of THU must be stored frozen and protected from light at –20°C. The material is hygroscopic and difficult to weigh, and reconstitution of the entire contents at the intended concentration is strongly suggested.

2) Following reconstitution in water (do not use methanol), aliquots can be purged with nitrogen and frozen at –20°C. The datasheets indicate that these aliquots are stable for up to 3 months at –20°C.

3) Prepare a 0.4 mg/mL solution of THU. Since the datasheet purity of THU is listed as >80% (by TLC), assume that there are 8 mg of THU per vial of THU. Diluting this to a volume of 20 mL in a graduated cylinder or graduated centrifuge tube gives a

0.4 mg/mL (400 ug/mL) solution of THU. Keep this solution on ice. Prepare the THU stock solution according to following instructions.

- Measure a volume of 10 mL of sterile water into a graduated cylinder or a 50 mL graduated centrifuge tube
- Use a serological pipette to add approximately 1 mL of water to the vial of THU.
- Mix/shake the THU vial as best as possible, until the powder seems to be dissolved in the water.
  - •Transfer the liquid from the THU vial into the graduated cylinder or graduated centrifuge tube
- Bring the total volume of the container to 20 ml. Transfer into 1 mL aliquots for freezing at -20C. Note: 1mL of THU stock solution (0.4 mg/mL) should be enough for 3 kits (27 Vacutainer Tubes).

B. Preparation of K2EDTA Vacutainer tubes containing THU (tetrahydrouridine)

1) Pop the top of the vacutainer tube to break the vacuum seal.
2) Using a pipettor, immediately deliver at least 20 uL (20-30 uL) of the THU stock solution (thawed the same day) into each of the labeled K2EDTA 6 mL Vacutainer tubes.
3) When all K2EDTA Vacutainer tubes have been prepared with the THU and labeled, store the tubes refrigerated for up to 30 days.
4) Toss the remainder of THU stock solution aliquot when done.

C. Details for Plasma Sample Collection:

Note: Blood samples for PK studies need to be processed immediately. It is imperative that the following procedures are followed after collection of the sample to stabilize the drug and its metabolite(s).

1) 1. Using a syringe, collect a 4 mL peripheral blood sample and transfer to the 6 mL K2EDTA tube preloaded with THU and stored at 4°C (see B above).
2) 2. Immediately upon collection, mix the blood sample by gently inverting the tube 8-10 times and then immediately place the tube in a container of wet ice. Samples must be kept on the wet ice until centrifuged. Use wet ice to avoid sample hemolysis.
3) 3. Centrifuge the EDTA tube, **pre-loaded with THU**, at either 1800 g x 10 min at 4°C or 1500 g x15 min at 4°C. **The tube should be centrifuged within 1 hour of collection.**

4) 4. Immediately after centrifugation, use the disposable pipette provided in the kit to transfer ~ 1.0 mL of plasma into each of 2 labeled screw top cryo tubes and store in a –70°C freezer until shipment.

5) 5. Store the samples in the freezer until packaging for shipment to the bioanalytical lab (Frontage Labs in Exton, PA). Samples should be shipped to Frontage for analysis within 90 days of collection, if possible (Or shipped once samples from all subjects are batched).

6) 6. Samples should be packaged for shipment in triple packaging, sealed and cushioned and containing sufficient amount of crushed dry ice to last the duration of transport. See Packaging Specimens for Shipment for details.

PK Specimen Collection guide

**PK Decitabine/SGI-110**	PK Deci/SGI (Per Timepoint)	**PK Deci/SGI A (Per Timepoint)**	6mL Lavender K2 EDTA Vacutainer Tube 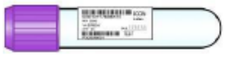	1.8mL NUNC Cryovial w/ White Insert 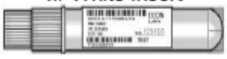	Frozen
		**PK Deci/SGI B (Per Timepoint)**		1.8mL NUNC Cryovial w/ White Insert 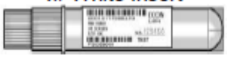	Frozen

D. Instructions for Completing the Sample Inventory Form:

1. Using a black or blue ball point pen, complete the following sections of the sample Inventory form:

   a) Subject ID Number
   b) Subject Initials (if allowed per ICF)
   c) Sample Collection Date and Time
   d) Number of plasma aliquots collected
   e) Visit Type

<u>Important Note</u>

Please note additional required information on comment field (e.g. low sample volume, sample not collected, apparent hemolyzed sample, etc.)

2. Store the Original copy at site. Include one copy of the sample inventory form when shipping samples to Frontage laboratory and send one copy to Astex to the attention of:

Mary Rosa

Astex Pharmaceuticals, Inc.

4420 Rosewood Drive, Suite 200

Pleasanton, CA 94588 USA

All required information must be accurate and complete to avoid a delay in analysis of samples.

SECTION 2. LABORATORY SUPPLIES

Laboratory supplies

Important Reminders for Sites

- DO NOT use an expired kit (The kit expiration date is located on the kit box label).
- Destroy expired and unused kits according to your site's hazardous waste disposal policy.
- Please note that Visit kits can be expensive, and sites should be mindful of wastage.

Preparing and Affixing Labels to Specimen Containers

Please utilize labels to ensure proper specimen identification and appropriately process each specimen. **Properly affixing the label is critical to expediting each specimen for testing.**

To properly affix the label:

- Match the appropriate label to the corresponding collection/transport tube.
- Affix the label oriented LENGTHWISE on the tube.
- Do not cover the stopper with the label.

A subject identification number is required for proper specimen container identification. Legibly print the subject identification number on EACH label provided BEFORE collecting specimens.

SECTION3. PROPERLY PACKAGING SPECIMENS FOR SHIPMENT

Proper specimen packaging is critical to specimen integrity and accurate laboratory test results.

This section contains comprehensive packaging instructions for each type of shipper provided

for this protocol.

Some important general reminders for properly preserving and packaging specimens are

provided below:

Maintain collected specimens at the appropriate temperature prior to shipment.

- Make certain that you have a sufficient supply of dry ice available for proper packaging of specimens. (See detailed instructions for Frozen Shippers in this section.)

- Verify that the requisition (Sample Inventory form) has been thoroughly and accurately completed.
- Prior to packaging specimens in shippers, make certain that each specimen container is properly labeled and identified.
- Ensure that the tube caps and screw tops are well secured.
- Batch ship (if possible) minimum 3-5 subjects' samples to Frontage Laboratories, Inc.

  Sample Coordinator Frontage Laboratories, Inc.
  700 Pennsylvania Drive
  Exton, PA 19341 P: 484-348-4790
  Email: SampleManagement@frontagelab.com
  Frozen Shipper- Cryobox Shipments

- Place dry ice in the bottom of the compartment. Note: Dry Ice pellets are recommended for optimal performance.
- Place cryovials into the sections of the cryobox.
- Place the lid securely on the cryobox.
- Place the cryobox into the large biohazard bag. Note: Do not place sample inventory form in bag.
- Place the sealed biohazard bags containing frozen specimens on top of the dry ice.
- Place dry ice on top of the biohazard bags.
- Please the lid securely on top of the insulated container. Place the completed sample inventory forms on top of the lid of the insulated container. Note: Please make two copies (send one copy with the samples and one to Astex)
- Close the slide flaps of the shipper box and then the main flap. Seal the box with the enclosed sealing strips.
- Affix the appropriately completed Air Waybill to the shipper.
