# Supplementary material for: Guadecitabine plus ipilimumab in unresectable melanoma: five-year follow-up and integrated multi-omic analysis in the phase 1b NIBIT-M4 trial
Source: Nat Commun. 2023 Sep 22;14:5914. doi: 10.1038/s41467-023-40994-4 (PMC10516894; doi:10.1038/s41467-023-40994-4)
Supplement: Supplementary file 3 — Description of Additional Supplementary Files [file 41467_2023_40994_MOESM3_ESM.pdf]

Description of Additional Supplementary Files

Supplementary Data 1. Somatic mutations of NIBIT-M4 cohort.

Supplementary Data 2. Differentially expressed genes between R vs. NR at baseline, week4 and week12 after treatment

Supplementary Data 3. Gene Set Enrichment analysis results from R vs. NR comparison at baseline, week4 and week12 after treatment

Supplementary Data 4. Predictive scores of response to ICI for NIBIT-M4 samples

Supplementary Data 5. GIE scores for NIBITM4 samples
