# Supplementary material for: Guadecitabine plus ipilimumab in unresectable melanoma: five-year follow-up and integrated multi-omic analysis in the phase 1b NIBIT-M4 trial
Source: Nat Commun. 2023 Sep 22;14:5914. doi: 10.1038/s41467-023-40994-4 (PMC10516894; doi:10.1038/s41467-023-40994-4)
Supplement: Supplementary file 9 — Reporting Summary [file 41467_2023_40994_MOESM9_ESM.pdf]

nature portfolio

Corresponding author(s): Michele Maio
Last updated by author(s): Aug 14, 2023

Reporting Summary

Nature Portfolio wishes to improve the reproducibility of the work that we publish. This form provides structure for consistency and transparency in reporting. For further information on Nature Portfolio policies, see our Editorial Policies and the Editorial Policy Checklist.

Statistics

For all statistical analyses, confirm that the following items are present in the figure legend, table legend, main text, or Methods section.

n/a	Confirmed	
[ ]	[x]	The exact sample size (*n*) for each experimental group/condition, given as a discrete number and unit of measurement
[ ]	[x]	A statement on whether measurements were taken from distinct samples or whether the same sample was measured repeatedly
[ ]	[x]	The statistical test(s) used AND whether they are one- or two-sided<br>*Only common tests should be described solely by name; describe more complex techniques in the Methods section.*
[x]	[ ]	A description of all covariates tested
[ ]	[x]	A description of any assumptions or corrections, such as tests of normality and adjustment for multiple comparisons
[ ]	[x]	A full description of the statistical parameters including central tendency (e.g. means) or other basic estimates (e.g. regression coefficient) AND variation (e.g. standard deviation) or associated estimates of uncertainty (e.g. confidence intervals)
[ ]	[x]	For null hypothesis testing, the test statistic (e.g. *F*, *t*, *r*) with confidence intervals, effect sizes, degrees of freedom and *P* value noted<br>*Give P values as exact values whenever suitable.*
[x]	[ ]	For Bayesian analysis, information on the choice of priors and Markov chain Monte Carlo settings
[x]	[ ]	For hierarchical and complex designs, identification of the appropriate level for tests and full reporting of outcomes
[ ]	[x]	Estimates of effect sizes (e.g. Cohen's *d*, Pearson's *r*), indicating how they were calculated

Our web collection on statistics for biologists contains articles on many of the points above.

Software and code

Policy information about availability of computer code

Data collection	No software or code was used to collect data.
Data analysis	The open source software used and described in Methods section are:<br>fastQC (v. 0.11.8), bwa (v. 0.7.17-r1188), GATK (v. 4.1.0.0), AnnoVar (v. 2017), snpEff (v. SnpEff 4.3i), CNVkit (v. 0.9.9), GISTIC (v. 2.0.23), deconstructSigsR package (v. 1.8.0), STAR (v. 2.7.0b),featureCounts (v. 1.6.3), EDAseq R package (v. 2.22.0), EdgeR R package (v. 3.30.3), clusterProfiler R package (v. 3.3.6), Rsubread R package (v. 2.10.5), trim galore (v. 0.6.5), Bismark (v. 0.22.3), RnBeads 2.0 R package (v. 2.6.0), limma R package (v. 3.44.3), GenomicRanges R package (v. 1.48.0), AnnotationHub R package (v. 3.4.0), liftOver R package (v. 1.20.0), MASS R package (v. 7.3-58.3), MiXCR software (v. 3.0.13), SMITE R package (v. 1.16.0), Polysolver (v. 4), pVACtools (v. 1.5.11).<br>Commercial software Sentieon Genomic Tool v. 201911 was used for somatic single-nucleotide variants (SNVs) and indels calling.<br>Commercial software nSolver software v. 4.0 was used for quality control, data normalization and analysis for NanoString data.

For manuscripts utilizing custom algorithms or software that are central to the research but not yet described in published literature, software must be made available to editors and reviewers. We strongly encourage code deposition in a community repository (e.g. GitHub). See the Nature Portfolio guidelines for submitting code & software for further information.

Data

Policy information about availability of data

All manuscripts must include a data availability statement. This statement should provide the following information, where applicable:

- Accession codes, unique identifiers, or web links for publicly available datasets
- A description of any restrictions on data availability
- For clinical datasets or third party data, please ensure that the statement adheres to our policy

The processed NanoString generated in this study have been deposited in the GEO under accession number: GSE211645. Raw data of Whole Exome Sequencing, RNA-Sequencing and Reduced Representation Bisulfite Sequencing are available under restricted access, for privacy of the patients, on the European Genome-phenome Archive under accession number EGAS00001006736. The genomic sequencing data are available under restricted access due to patient confidentiality. Access for research purposes can be obtained by applying to the data access committee EGAC00001002947 via EGA. It is expected that data will be available within 3 months of the request and there are no restrictions on the duration of access.
The complete de-identified clinical data are available under restricted access. Data access can be obtained by request from segreteria@fondazionenibit.org. The data that will be shared include individual participant data that underlie the results reported in this paper after de-identification (text, table, figures, and appendices). The time frame for response to requests will be within four weeks. Data will be shared for non-commercial purposes after approval of a proposal by the Board of the NIBIT Foundation and with a signed data access agreement. The availability of such data will begin 3 months and end 24 months after article publication.
The study protocol is available as Supplementary Note in the Supplementary Information file.
The remaining data are available within the Article, Supplementary Information or Source Data file.
Previously published genomic data of independent cohorts used to support the findings of this study were obtained from the CBioPortal for Cancer Genomic (https://www.cbioportal.org/study/summary?id=mel_ucla_2016,https://www.cbioportal.org/study/summary?id=mel_dfci_2019,https://www.cbioportal.org/study/summary?id=skcm_mskcc_2014) and from GEO under Series accession number: GSE188250.

Human research participants

Policy information about studies involving human research participants and Sex and Gender in Research.

Reporting on sex and gender	Gender has been reported for all enrolled patients: 17 (89%) male patients and 2 (11%) female patients were treated in the study. Demographic and baseline characteristics were summarized for all enrolled subjects using descriptive statistics.
Population characteristics	Adult patients with unresectable stage III or IV cutaneous melanoma, measurable lesions by CT or MRI per WHO criteria that were amenable to biopsy, life expectancy 16 weeks, Eastern Cooperative Oncology Group performance status 1, and who had received no more than one line of therapy for advanced disease were eligible for inclusion.<br>Median age was 58 (range, 27-86); 16 (84%) patients have an ECOG performance status of 1. Two (11%) patients were unresectable stage III and 11(58%) stage IV M1a.<br>Demographic and baseline characteristics were summarized for all enrolled subjects using descriptive statistics.
Recruitment	Patients were recruited from internal database, referring physicians. No potential self-selection bias have been identified.
Ethics oversight	The study was conducted in accordance with the ethical principles of the Declaration of Helsinki and the International Conference on Harmonization of Good Clinical Practice. The protocol was approved by the independent ethics committee of the University Hospital of Siena (Siena, Italy). All participating patients (or their legal representatives) provided signed-informed consent before enrollment.

Note that full information on the approval of the study protocol must also be provided in the manuscript.

Field-specific reporting

Please select the one below that is the best fit for your research. If you are not sure, read the appropriate sections before making your selection.

☒ Life sciences ☐ Behavioural & social sciences ☐ Ecological, evolutionary & environmental sciences

For a reference copy of the document with all sections, see nature.com/documents/nr-reporting-summary-flat.pdf

Life sciences study design

All studies must disclose on these points even when the disclosure is negative.

Sample size	To confirm the statistical power to detect a significant difference between responder (R) and non responder (NR) patients, we conducted a post-hoc power analysis using sample size and a hypothesis of effect size to detect. We used in all our comparisons a significance level of 0.05 and assumed a two-tailed test. The reported comparison includes the differential analyses at each time between R and NR using both gene

	expression and methylation profiling. With an estimated guess of effect size (Cohen's d) of 1.5 and a sample size of 14 (8 in NR and 6 in R), the post-hoc power analysis revealed that our study had a statistical power of 72% to detect a significant difference between the two groups.
Data exclusions	No data were excluded from the analyses
Replication	All attempts to replicate the analysis were successful
Randomization	Patients allocation was not random; this is a phase Ib , dose-escalation study, with a standard 3+3 study design.
Blinding	The investigators were blinded to group allocation during data collection and analysis

Reporting for specific materials, systems and methods

We require information from authors about some types of materials, experimental systems and methods used in many studies. Here, indicate whether each material, system or method listed is relevant to your study. If you are not sure if a list item applies to your research, read the appropriate section before selecting a response.

Materials & experimental systems

n/a	Involved in the study	
☐	☒	Antibodies
☒	☐	Eukaryotic cell lines
☒	☐	Palaeontology and archaeology
☒	☐	Animals and other organisms
☐	☒	Clinical data
☒	☐	Dual use research of concern

Methods

n/a	Involved in the study	
☒	☐	ChIP-seq
☒	☐	Flow cytometry
☒	☐	MRI-based neuroimaging

Antibodies

Antibodies used	The antibodies used for Immunohistochemistry assay are: anti-CD8 clone 524 C8/144B (M7103, Dako) at a final concentration of 3.14 µg/mL and anti-HLA Class I, clone EMR8-5 (ab70328, abcam) at a final concentration of 0.3 µg /mL. The HRP labeled polymer 525 conjugated EnVision+ Single Reagent (Dako, K4001) was used as a secondary antibody.
Validation	Each antibody was validated according to manufacturer's instruction.<br><br>Additional information:<br>1. Anti-CD8, Clone: C8/144B, Source: M7103, Dako<br>Species: Mouse Anti-Human; (https://www.agilent.com/en/product/immunohistochemistry/antibodies-controls/primary antibodies/cd8-(dako-omnis)-76236#literature)<br>Specificity: SDS-PAGE analysis of immunoprecipitates formed between lysates of 125I-labeled human T lymphoblasts and the antibody shows reaction primarily with a 32 kDa polypeptide corresponding to CD8α; (https://www.agilent.com/en/product/immunohistochemistry/antibodies-controls/primary-antibodies/cd8-(dako-omnis)-76236#literature)<br><br>2. Anti-HLA class I, Clone: EMR8-5, Source: ab70328, abcam<br>Species: Mouse Anti-Human<br>Specificity: ab70328 reacts with the heavy chains of human HLA class 1 A, -B, and -C.<br>Positive control: IHC-P: Human tonsil tissue sections<br>ab70328 has been referenced in 141 publications (https://www.abcam.com/products/primary-antibodies/hla-class-1-abc-antibody-emr8-5-ab70328.html#description_references)

Clinical data

Policy information about clinical studies
All manuscripts should comply with the ICMJEguidelines for publication of clinical research and a completedCONSORT checklist must be included with all submissions.

Clinical trial registration	NCT02608437
Study protocol	The study protocol will be provided as supplementary note (attached)
Data collection	The Italian Network for Tumor Biotherapy (NIBIT) Foundation undertook the NIBIT-M4 phase Ib, dose-escalation, single-center study between October 2015 and August 2018 at the Center for Immuno-Oncology of Siena, Italy.
Outcomes	Primary Objective was Maximum Tolerated Dose (MTD) and safety of SGI-110 in combination with ipilimumab in 21 day cycles in melanoma patients.<br>Secondary Objectives were immune-related (ir) -Disease Control Rate (ir-DCR), immune-related (ir) -Objective Response Rate (ir-ORR), immune-related (ir) -Time to Response (ir-TTR) and immune-related (ir) -Duration of Response (ir-DOR), median immune-related (ir) Progression Free Survival (ir-PFS), median Overall Survival (OS), and survival rate at 1 and 2-years.<br>Exploratory Objectives were immune-biologic correlates to treatment with SGI-110 in combination with ipilimumab and the pharmacokinetic profile of SGI-110 and decitabine.
